# A pilot study to assess the feasibility of a remotely monitored high-intensity interval training program prior to allogeneic hematopoietic stem cell transplantation

**DOI:** 10.1371/journal.pone.0293171

**Published:** 2023-11-30

**Authors:** Ashley L. Artese, Hilary M. Winthrop, Lauren Bohannon, Meagan V. Lew, Ernaya Johnson, Grace MacDonald, Yi Ren, Amy M. Pastva, Katherine S. Hall, Paul E. Wischmeyer, David Macleod, Jeroen Molinger, Stratton Barth, Sin-Ho Jung, Harvey Jay Cohen, David B. Bartlett, Anthony D. Sung

**Affiliations:** 1 Center for the Study of Aging and Human Development, Duke University Medical Center, Durham, North Carolina, United States of America; 2 Department of Exercise Science and Health Promotion, Florida Atlantic University, Boca Raton, Florida, United States of America; 3 Division of Hematologic Malignancies and Cellular Therapy, Department of Medicine, Duke University Medical Center, Durham, North Carolina, United States of America; 4 Duke Molecular Physiology Institute, Duke University School of Medicine, Durham, North Carolina, United States of America; 5 Division of Medical Oncology, Duke University School of Medicine, Durham, North Carolina, United States of America; 6 Duke Cancer Institute Biostatistics Shared Resources, Duke University School of Medicine, Durham, North Carolina, United States of America; 7 Department of Orthopaedic Surgery, Physical Therapy Division, Duke University School of Medicine, Durham, North Carolina, United States of America; 8 Geriatric Research, Education and Clinical Center, Durham Veterans Affairs Medical Center, Durham, North Carolina, United States of America; 9 Department of Medicine, Division of Geriatrics, Duke University, Durham, North Carolina, United States of America; 10 Duke University Hospital, Department of Anesthesiology and Surgery, Durham North Carolina, United States of America; 11 Human Pharmacology and Physiology Lab, Department of Anesthesiology, Duke University Medical Center, Durham, North Carolina, United States of America; 12 Department of Biostatistics and Bioinformatics, Duke University School of Medicine, Durham, North Carolina, United States of America; 13 Department of Medicine, Duke University Medical Center, Durham, North Carolina, United States of America; 14 School of Biosciences and Medicine, University of Surrey, Guildford, United Kingdom; IRCCS Medea: Istituto di Ricovero e Cura a Carattere Scientifico Eugenio Medea, ITALY

## Abstract

**Introduction:**

Although allogeneic hematopoietic stem cell transplantation (HCT) can be a curative therapy for hematologic disorders, it is associated with treatment-related complications and losses in cardiorespiratory fitness and physical function. High-intensity interval training (HIIT) may be a practical way to rapidly improve cardiorespiratory fitness and physical function in the weeks prior to HCT. The primary aim of this study was to assess the feasibility of implementing a pre-HCT home-based HIIT intervention. The secondary aim was to evaluate pre to post changes in cardiorespiratory fitness and physical function following the intervention.

**Methods:**

This was a single-arm pilot study with patients who were scheduled to undergo allogeneic HCT within six months. Patients were instructed to complete three 30-minute home-based HIIT sessions/week between the time of study enrollment and sign-off for HCT. Sessions consisted of a 5-minute warm-up, 10 high and low intervals performed for one minute each, and a 5-minute cool-down. Prescribed target heart rates (HR) for the high- and low-intensity intervals were 80–90% and 50–60% of HR reserve, respectively. Heart rates during HIIT were captured via an Apple Watch and were remotely monitored. Feasibility was assessed via retention, session adherence, and adherence to prescribed interval number and intensities. Paired t-tests were used to compare changes in fitness (VO_2peak_) and physical function [Short Physical Performance Battery (SPPB), 30-second sit to stand, and six-minute walk test (6MWT)] between baseline and sign-off. Pearson correlations were used to determine the relationship between intervention length and changes in cardiorespiratory fitness or functional measures.

**Results:**

Thirteen patients (58.8±11.6 years) participated in the study, and nine (69.2%) recorded their training sessions throughout the study. Median session adherence for those nine participants was 100% (IQR: 87–107). Adherence to intervals was 92% and participants met or exceeded prescribed high-intensity HR on 68.8±34.8% of intervals. VO_2peak_ improved from baseline to sign-off (14.6±3.1 mL/kg/min to 17.9±3.3 mL/kg/min; p<0.001). 30-second sit to stand and SPPB chair stand scores significantly improved in adherent participants. Improvements in 30-second sit to stand (13.8±1.5 to 18.3±3.3 seconds) and 6MWT (514.4±43.2 to 564.6±19.3) exceeded minimal clinically important improvements established in other chronic disease populations, representing the minimum improvement considered meaningful to patients.

**Conclusions:**

Findings demonstrate that implementing a pre-HCT home-based remotely monitored HIIT program is feasible and may provide benefits to cardiorespiratory fitness and physical function.

## Introduction

Allogeneic hematopoietic stem cell transplantation (HCT) is a treatment approach for a variety of malignant and non-malignant hematologic disorders [1]. The intensive treatment regimen consists of conditioning (chemotherapy and/or radiation) followed by infusion of donor hematopoietic stem cells [1]. Although it can be a potential curative therapy, HCT is associated with treatment-related complications [1, 2] and accelerated losses in cardiorespiratory fitness, physical function, and strength, which can negatively impact performance of activities of daily living, independence, and quality of life (QOL) [3–5]. These negative changes are especially concerning as patients are already susceptible to functional impairments prior to undergoing HCT [6, 7]. Pre-HCT cardiorespiratory fitness and physical function have been found to be significantly lower than age-predicted normative values [6–8], and the prevalence of frailty among young and older patients prior to HCT is higher than that of community-dwelling older adults [7, 9–12].

Poor cardiorespiratory fitness has been associated with a longer hospital stay during HCT, greater number of hospitalized days before Day 100, and higher risk for mortality [13]. Lower functional performance is also predictive of disease relapse and worse overall survival [11, 13–16]. Jones et al. [15] reported a 9% relative risk reduction for non-relapse and overall mortality for every 50-meter increase in pre-HCT six-minute walk test (6MWT) distance. Furthermore, risk for non-relapse mortality was highest among patients with poor pre-HCT 6MWT performance (<400 meters) who experienced further declines after HCT [15]. Since these studies suggest that pre-HCT functional capacity can influence post HCT outcomes and survival, more research is needed to identify strategies to improve fitness and physical function prior to HCT. In addition, given that patients experience further losses in functional capacity following HCT, which may limit their ability to engage in more vigorous physical activity, the pre-HCT period may be an opportune time to prescribe higher intensity activities that can rapidly improve fitness and function. Therefore, implementing high-intensity exercise interventions for patients prior to HCT may be a useful strategy to increase physiological reserve, potentially mitigate losses following HCT, and improve post-HCT health and survival outcomes.

High-intensity interval training (HIIT) is a type of training that consists of repeated short bouts of exercise performed at or close to maximal levels of exertion interspersed by a low-intensity recovery phase [17]. In addition to being safe and feasible for individuals with chronic diseases including cancer [18–20], it can rapidly improve exercise capacity to the same extent as moderate-intensity aerobic exercise, but with less training time and volume [21]. Therefore, it may be a practical exercise mode for HCT patients given the limited window of time to implement an exercise intervention prior to HCT. Despite the benefits of HIIT, traveling to an exercise facility may pose safety risks for patients who are at increased risk for infections and a center-based program may not be feasible for patients who travel several hours to receive quaternary care at a transplant center. Thus, a home-based HIIT program may be a more practical and safe option. While unsupervised HIIT programs are beneficial [22], monitoring adherence to components specific to HIIT protocols (i.e., training time, interval number, and prescribed intensity) for home-based interventions is challenging, which may limit interpretation of findings. Two previous studies have implemented a home-based prehabilitative interval training intervention in HCT recipients [23, 24]; however, heart rate (HR) was not continuously monitored during the training sessions, thus limiting analysis of adherence to the prescribed intervals and intensities. This warrants more research to establish strategies for monitoring intervention adherence for a home-based HIIT program to determine its feasibility in this population. Therefore, the primary aim of this study was to assess the feasibility of implementing a home-based HIIT program in patients planning to undergo HCT and evaluate the use of mobile health (mHealth) technology to assess adherence to the training program. Based on previous adherence results from a pre-HCT interval training trial that reported a median adherence of 47%, with only 35% of patients completing ≥ 75% of sessions [24], we hypothesized that at least 50% of patients would complete ≥50% of their HIIT sessions and prescribed intervals. In addition, a secondary aim was to evaluate the pre- and post-intervention changes in cardiorespiratory and physical function outcomes in patients prior to undergoing HCT.

## Materials and methods

### Overview

This was a single-arm pilot study to evaluate the feasibility of a pre-HCT, mobile-health supported, home-based, HIIT program for patients prior to receiving allogeneic HCT. The trial was registered with clinicaltraials.gov (NCT03823651). Participants provided written informed consent, which was approved by the Duke Health Institutional Review Board (IRB # Pro00092963). Following consent, participants completed a demographics questionnaire and baseline assessments at the Duke Adult Bone Marrow Transplant (ABMT) clinic, followed by an introductory HIIT session. After the introductory session, participants completed three home-based HIIT sessions per week on their own, with support by weekly check-ins and video conferencing as needed. Post assessments were completed in person at sign-off, just prior to transplant. Aside from the introductory session, all study procedures were conducted remotely or coincided with regular clinical visits.

### Participants

Patients were recruited from the ABMT between June 2019 and May 2022. Adults between the ages of 18–80 years who were scheduled to undergo allogeneic HCT for cancer or a non-cancer illness within six months were eligible to participate. Exclusion criteria included an impairment that precluded them from exercise, an absolute contraindication to exercise [25], or inability to read or follow directions in English. In addition, as part of the baseline testing, participants completed a cardiopulmonary exercise test (CPET). Results were reviewed by a cardiologist, and those who did not receive cardiology clearance to exercise were unable to participate in the study and instead referred to appropriate care.

### Intervention

Since this was a single-arm study, all participants received the HIIT intervention. The intervention was developed as a home-based training program due to the following reasons: 1) many patients live far from the ABMT, and thus home-based training is a more realistic option for patients; 2) due to patients’ compromised immune system, attending a fitness and wellness center may not be safe for exercise training; and 3) we have the ability to track exercise adherence to the training intervention using mHealth technology.

The intensity of the intervention was individualized to each participant with prescribed HRs for the high- and low-intensity intervals determined from the maximum HR achieved at VO_2peak_ during the CPET. Interval intensity was calculated as a percentage of HR reserve (HRR) using the following equation: ((maximum HR–HR rate) x training intensity) + resting HR. Heart rates for the high- and low-intensity intervals were prescribed at 80–90% and 50–60% of HRR, respectively. Participants were instructed to complete three HIIT sessions per week for a total of 30 minutes per session, beginning with a 5-minute warm-up at 40–50% HRR, followed by 20 minutes of interval training, and ending with a 5-minute cool-down at 40–50% of HRR. The 20-minute interval portion consisted of 10 total intervals at a work-to-rest ratio of 1:1, which corresponded to one minute of high-intensity (80–90% of HRR) followed by one minute of low-intensity (50–60% of HRR) exercise. Although participants completed the HIIT sessions on the days and times that were convenient to them, they were encouraged to perform the HIIT sessions on non-consecutive days with at least 48 hours between sessions.

Following baseline assessments, participants were introduced to the HIIT protocol by a certified exercise physiologist who was trained on the study protocol and intervention administration procedures. During this in-person introductory session, the exercise physiologist reviewed all aspects of the training program with the participant including prescribed frequency, intensity, duration of the sessions, and timing of intervals. Following the review, the participant performed a practice HIIT session under the supervision of the exercise physiologist using a mode closest to the participant’s self-selected mode for the home-based sessions (i.e. walking, jogging, stair climbing, cycling, etc). The practice session included a warm-up, 3–6 intervals at the prescribed intensities, and a cooldown to familiarize participants with the protocol. Heart rate was monitored continuously throughout the session by the exercise physiologist via the Polar OH1 HR monitor (Polar USA, Lake Success, NY). Rating of perceived exertion was assessed using the 6–20 Borg scale [26]. Following the introductory training session, participants were instructed to complete three sessions per week at their home or a location of their choice (i.e. outdoors trails, medical fitness center, etc). The first home-based session was supervised via video conferencing. After the first session, participants completed the rest of the training sessions unsupervised. They were given a paper log to track exercise session dates and times.

Participants were instructed to maintain the prescribed frequency, intensity, session time, interval ratio, and interval time throughout the duration of the study. While participants were encouraged to start slowly and work up to the 10 intervals over the first 1–2 weeks, no further progressions were implemented since exercise progression for HIIT is inherent to the program: as participants’ cardiovascular fitness improves, the workload required to reach the prescribed HRs will most likely increase. Therefore, participants were encouraged throughout the intervention period to maintain the prescribed HRs for the high- and low-intensity intervals. The length (in weeks) of the intervention varied among participants and was dependent on the length of time between the baseline assessments and sign-off for transplant.

Participants were provided two mobile devices throughout the duration of the study that allowed for tracking and monitoring of HRs: 1) an Apple Watch (Apple Inc., Cupertino, CA) to wear daily and during the training sessions so that participants could monitor their HRs throughout the workout; 2) an iPhone with an mHealth application that transferred HR data recorded by the Apple Watch to a protected server for researchers to access. The mHealth application installed was either Technology Recordings to better Understand BMT (TRU-BMT) [27] or SplendoFit (Splendo Health B.V.). The iPhone also served as a way for participants to communicate with the exercise physiologist via text messaging and allowed them to send pictures or texts of their exercise log on a weekly basis.

With the exception of the introductory sessions, and per request as needed, all of the training sessions were unsupervised; however, the exercise physiologist was available to provide additional training or support through phone calls or video conferencing using the iPhone provided to participants. The exercise physiologist checked in with participants on a weekly basis via phone calls or text messages (based on participant preference) to provide encouragement and support as well as answer participants’ questions. In addition, HR data from the HIIT sessions were downloaded by the exercise physiologist on a weekly basis and graphed to identify adherence to prescribed interval number and HR zones. The exercise physiologist provided participants with feedback and guidance based on whether the exercise intensity was higher or lower than the prescribed HR zones.

### Outcomes

Feasibility was assessed via recruitment, retention, session adherence, exercise prescription adherence, mHealth utility, and adverse events. Recruitment was calculated as the percentage of approached participants who consented to participate in the study. Based on recruitment rates from previous cancer trials and overall healthcare exercise trials, we considered a recruitment rate ≥50% as sufficient [28]. Participant retention was defined as the number of enrolled patients who participated in the HIIT intervention and logged/reported their training sessions throughout the duration of the intervention. Session adherence was calculated as the percentage of prescribed sessions completed. Adherence to the exercise prescription was determined by the percentage of prescribed intervals completed per session and the percentage of high- and low-intensity intervals that met prescribed HR zones. Based on adherence results from a previous pre-HCT interval training trial [24], we determined retention and adherence as sufficient if 50% of enrolled patients completed ≥50% of their HIIT sessions and prescribed intervals. Maximum HR for each session was also calculated by averaging the maximum HR reached for each interval. The maximum HR for each session was averaged across all sessions for each participant to determine the mean maximum HR for the high-intensity intervals. mHealth utility was determined as a percentage of completed sessions where exercise HR data could be retrieved through the database. Adverse events were monitored and recorded in case report forms.

Secondary outcomes included changes in cardiorespiratory fitness and physical function. Cardiorespiratory fitness was assessed just prior to the start of the intervention on a cycle ergometer using a medically supervised CPET, 12-lead electrocardiogram, and breath-by-breath metabolic analysis (COSMED Quark CPET, Rome, Italy). The test utilized a ramp protocol (15–20 Watts/min), with VO_2peak_ recorded as the highest 30-second value elicited during the CPET. Physical function was assessed within three weeks prior to the intervention and coincided with clinical visits. Physical function assessments included the Short Physical Performance Battery (SPPB), 30-second sit to stand, and 6MWT. The SPPB was conducted first and consisted of three tests: balance, gait speed, and five repeated chair stand test [29]. For the balance tests, participants were scored based on their ability to hold three standing positions for a maximum of 10 seconds: side-by-side, semi-tandem, and tandem stand. Gait speed was determined by the time it took to complete a four-meter walk, with the best of two trials recorded. For the five repeated chair stand test, the time it took to complete five chair stands was recorded. Points were assigned to each test domain, with a maximum SPPB score of 12 points. The 30-second sit to stand was completed next, and the number of chair stands completed in 30 seconds was recorded [30]. For the final functional assessment, the 6MWT, participants walked back and forth along a 30-meter hallway to cover as much distance as possible in six minutes [31]. The distance completed was recorded.

### Statistics

The primary hypothesis for safety and feasibility was that at least 50% of enrolled patients would complete ≥50% of their HIIT sessions and prescribed intervals. Therefore, we planned to enroll a sample size of 35 patients, which would be able to estimate a completion rate of 50% to within a 95% confidence interval of ±19%. For higher completion rates, such as 75%, we would be able to estimate to within a 95% confidence interval of ±17% [32]. Participant demographics, feasibility outcomes (retention, session and exercise prescription adherence, mHealth utility, and adverse events), and functional assessments are presented using descriptive statistics (mean ± standard deviation, median (interquartile range (IQR)), or frequencies and percentages). Paired t-tests were used to compare changes in fitness and functional measures between baseline and sign-off assessments. Pearson’s correlations were conducted to determine the relationship between intervention length and changes in cardiorespiratory fitness or functional measures. Significance was accepted at p<0.05. All analyses were conducted using IBM SPSS version 28.0.

## Results

### Recruitment

**Fig 1:**
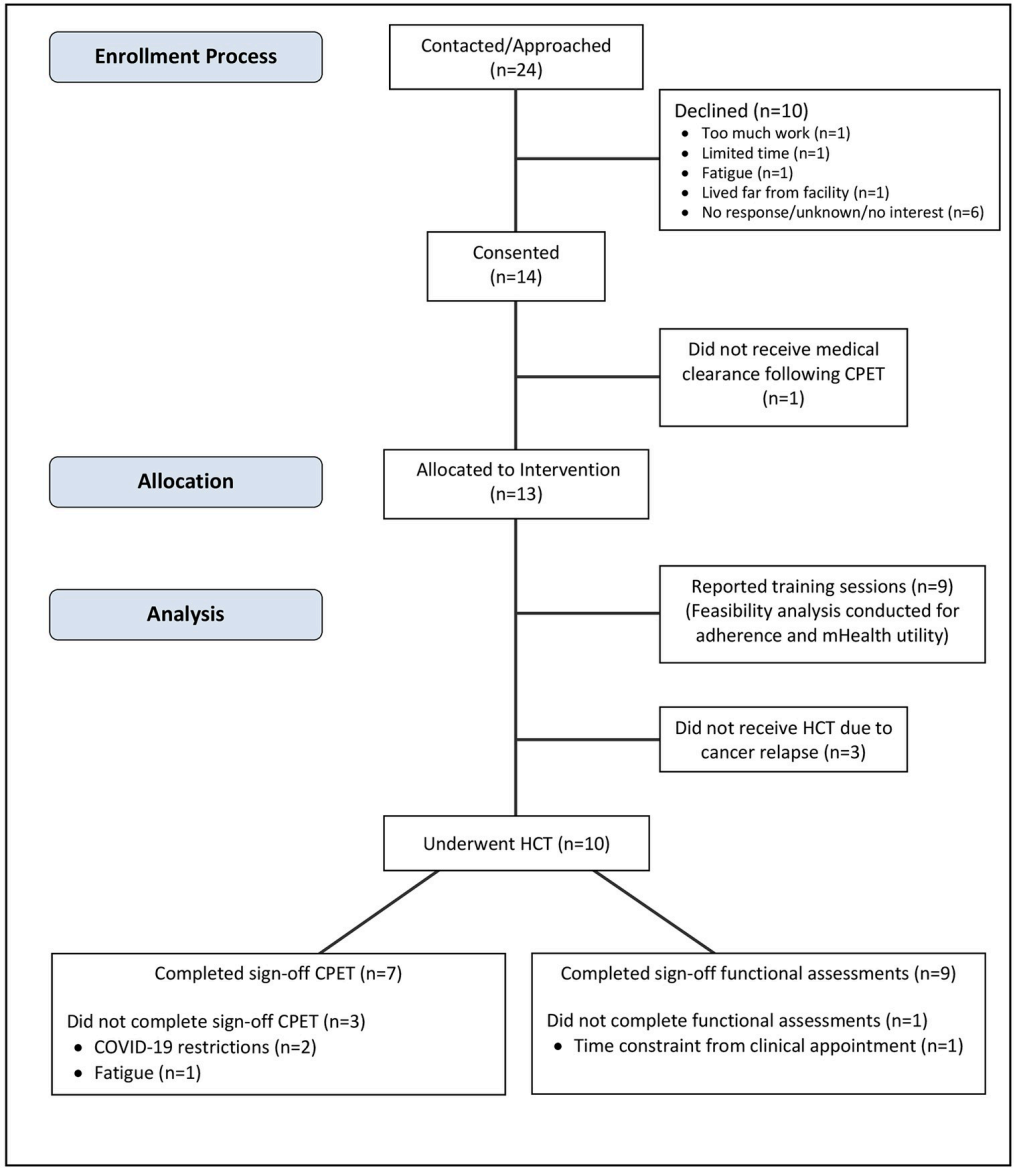
Study flow diagram. CPET: cardiopulmonary exercise test; HCT: Hematopoietic stem cell transplantation.

Of the 24 eligible patients who were contacted or approached by the research team regarding potential participation in the study, fourteen patients (58.3%) consented to participate (Fig 1). Reasons for not consenting to the study included: 1) trial required too much work (n = 1); 2) limited time to participate due to other family priorities (n = 1); 3) fatigue (n = 1); 4) lived far from facility and wanted to mitigate travel and time at facility (n = 1); 5) Not interested/no response/unknown (n = 6). The trial was concluded before reaching the goal sample size as the COVID-19 pandemic interrupted recruitment, and then the award funding the study reached its end date. Of those who consented, one did not gain medical clearance following the CPET and thus was taken off study. Therefore, 13 participants (58.8 ± 11.6 years; male: 10, female: 3) were prescribed HIIT. Baseline participant characteristics are presented in Table 1. Ten participants proceeded to HCT with an average time of 15.3 ± 8.9 weeks between enrollment and sign-off for HCT. Three participants did not undergo transplant due to cancer relapse (n = 3). Even though these three participants did not reach sign-off, their data were included in the feasibility analysis since two of the three participants completed the intervention for a total of 12 and 21 weeks each until it was determined that they would not undergo transplant. The third participant did not log any training sessions or respond to the exercise physiologist about training sessions.

**Table 1:** Participant characteristics (N = 13).

**Variable**	**Mean ± SD**	**Min-Max**
Age (yrs)	58.8 ± 11.6	34.2–76.4
Time between enrollment and sign-off (weeks)	15.3 ± 8.9^a^	3.7–31.9
Weight (kg)	90.8 ± 19.7	62.3–133.4
BMI (kg/m^2^)	29.5 ± 4.8	20.8–37.7
**Variable**	**Frequency**	**Percentage (%)**
*Gender*		
Male	10	76.9
Female	3	23.1
*Ethnicity*		
Hispanic or Latino	1	7.7
Non-Hispanic or Latino	12	92.3
*Race*		
White	10	76.9
Black or African American	3	23.1
*Diagnosis*		
Myelodysplastic syndrome	5	38.5
Acute myeloid leukemia	3	23.1
Diffuse large B-cell lymphoma	2	15.4
Acute lymphoblastic leukemia	1	7.7
Chronic lymphocytic leukemia	1	7.7
Peripheral T-cell lymphoma	1	7.7
*Receiving chemotherapy during intervention*		
Yes	10	76.9
No	3	23.1

Values are means ± standard deviations. BMI: Body mass index

^a^Indicates duration for participants who completed baseline assessments and were scheduled for transplant (n = 10)

### Program retention and adherence

Of the 13 participants who were prescribed the HIIT intervention, ten participants recorded and reported exercise session dates and times to the study team. Three participants did not record or report any exercise session dates despite multiple efforts by researchers to contact them. Uploaded data for those three participants indicated that one participant never wore the Apple Watch, one wore the watch for the first 13 of the 118-day period between enrollment and sign-off, and the third participant wore it for the first 4 of the 84-day intervention period. No HIIT sessions were identified from the data during those wear days, suggesting that the participants did not start the intervention. Therefore, adherence to training sessions and intervals for those three participants could not be determined.

#### Retention

Of the ten participants who reported any training sessions, nine consistently reported their weekly training sessions and participated in the intervention throughout the duration of the study: seven participants participated in the weekly HIIT sessions from the time between baseline assessments and sign-off and two participants who did not undergo transplant completed the weekly training sessions until they were withdrawn from the study (did not complete sign-off assessments). One participant reported exercise sessions for the first eight weeks, but did not report sessions for the next 24 weeks up to sign-off. Therefore, only nine (69.2%) participants participated in HIIT and followed reporting protocols for the intervention while four (30.8%) did not report sessions or participate in the intervention. A comparison of baseline age, body mass index (BMI), cardiorespiratory fitness, and functional measures (SPPB, 6MWT, and 30-second sit to stand) showed that the nine participants who participated/reported sessions had a significantly lower BMI (27.7 kg/m^2^) compared to the four who did not (33.6 kg/m^2^). There were no differences for age, cardiorespiratory fitness, or functional measures.

#### mHealth utility

**Fig 2:**
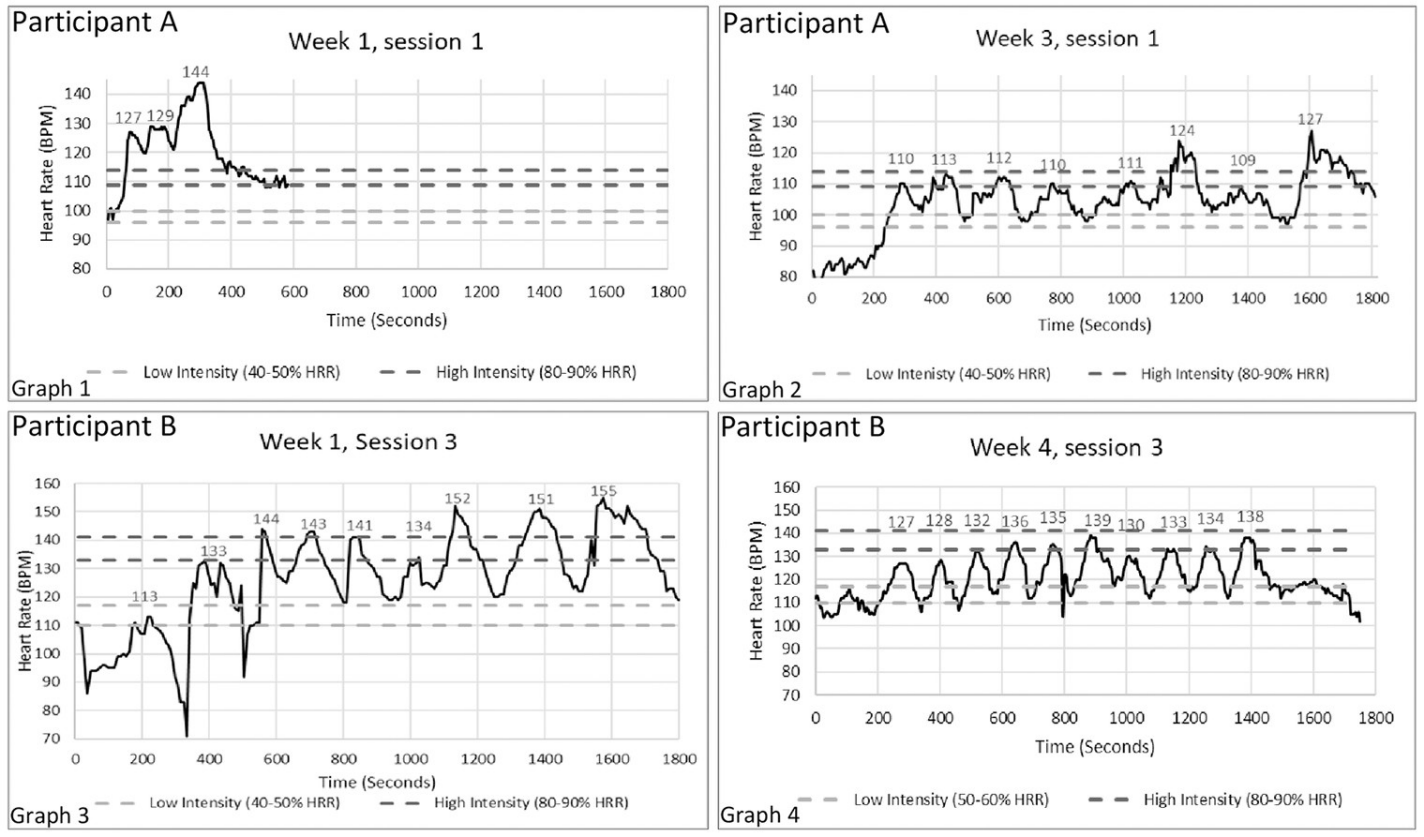
Sample high-intensity interval training (HIIT) session heart rate graph. The graphs above show two HIIT sessions from two participants. The dark gray dotted lines represent the target HR zone for the high-intensity intervals (80–90% of HRR) and the light gray dotted lines represent the target HR zone for the low-intensity intervals (50–60% HRR). Each spike in HR represents an interval that was completed during the 30-minute HIIT session. Numbers above the intervals indicate maximum HR reached during the interval. **Graphs 1** and **2** are from Participant A (age: 66 years; VO_2peak_: 10.6 mL/kg/min) during the first and third weeks of the program. During the first session (**Graph 1**), the participant overexerted himself, exceeding the prescribed target HR zones, and struggled to lower HR to the low-intensity HR prescription. After the first three intervals, the participant was not able to continue with the HIIT session. **Graph 2** demonstrates a HIIT session from the same participant during week 3. With feedback, coaching, and consistent weekly training, adherence to the prescribed session duration, interval number, and intensities improved with the participant completing eight intervals in the session, with most meeting the target HR zones. **Graphs 3** and **4** represent HIIT sessions from week 1 and week 4 from a more fit participant (Participant B; age: 45 years; VO_2peak_: 18.1 mL/kg/min). Although the participant was able to complete the 30-second session and intervals during week 1, the week 4 session shows improved adherence to the prescribed intervals and exercise intensities.

Uploaded HR data for each participant’s HIIT session was downloaded from the TRU-BMT or SplendoFit database on a weekly basis. Fig 2 shows sample HIIT session graphs from two participants and demonstrates how feedback and coaching improved adherence to intervals and intensities. Heart rate data were retrieved from 72.5% of reported sessions. Main reasons for having missing data for 27.5% of sessions included participants forgetting to wear the Apple Watch during the session, improper positioning of the watch on the participants’ wrist (consistent HR data was not recorded if watch was too loose), and issues with HR data transferring from the watch into the databases. Data from the one participant who reported only the first eight weeks of exercise could not be retrieved due to issues with the HR data transferring to the database. Therefore, adherence to the training sessions and prescribed intensity was analyzed for nine participants.

#### Adherence

**Fig 3:**
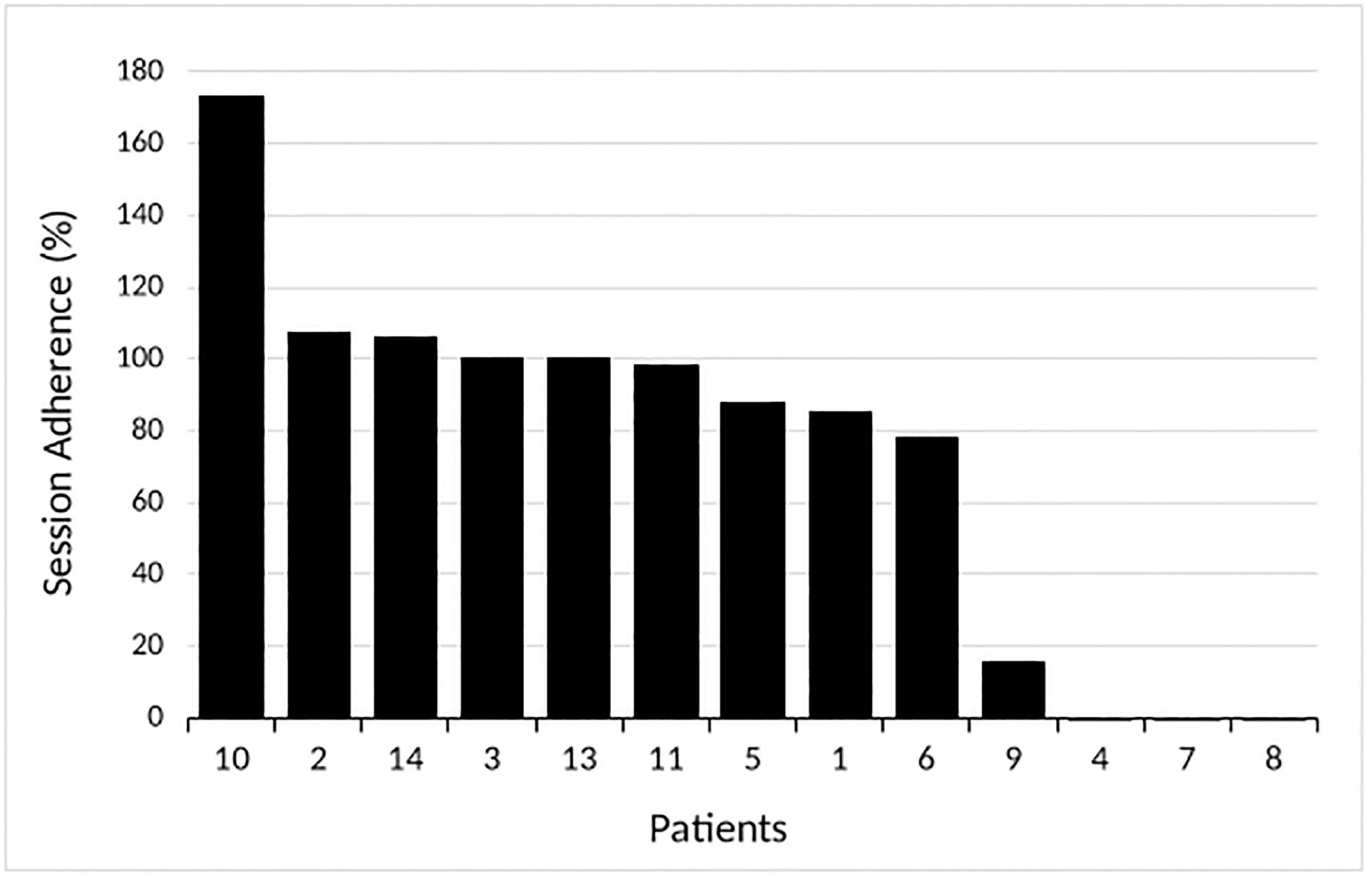
Adherence rate (% of prescribed sessions) for each participant enrolled in the study.

Fig 3 presents adherence for each of the 13 participants. Among the nine participants who reported their sessions for the duration of the study, there was a mean adherence of 104 ± 28%, with a median session adherence of 100% (IQR 87–107). Adherence was high due to two participants adhering to 100% of their prescribed 3 sessions/week and three participants averaging more than the prescribed 3 sessions/week. Session adherence for the other four participants ranged from 78% to 98%. Mean adherence to prescribed sessions and prescribed minutes for the entire sample, including the three participants who did not report any sessions and the one participant who only reported the first 8 of 32 weeks, was 73 ± 53% and 73 ± 58%, respectively.

Table 2 shows results for adherence to the exercise sessions, prescribed minutes, and training intensity for the nine participants. The number of completed intervals per session and percentages of intervals reaching prescribed target HRs were calculated based on the number of sessions where the HR data were available for the duration of the study. Participants completed an average of 9.2 ± 2.3 intervals per session. Participants reached a maximum HR ≥ 80% of HRR on 68.8 ± 34.8% of intervals. The prescribed low intensity HR range (50–60% of HRR) was achieved on 33.0 ± 15.7% of the low intensity intervals with 43.6 ± 23.7% exceeding the recommended intensity. When accounting for 0% adherence to interval number for the four participants who did not complete the intervention, average number of intervals per training session for the entire sample was 6.4 ± 4.8.

**Table 2:** Adherence to training sessions and prescribed intensity (n = 9).

Variable	Value	Min—Max
Weeks of HIIT completed (# of weeks)	13.8 ± 7.5	3.0–26.0
Weekly sessions completed (# of sessions)	3.1 ± 0.8	2.3–5.2
Adherence to prescribed sessions (median (IQR))	100.0 (87–107)	77.8–173.3
Adherence to prescribed minutes (median (IQR))	100.0 (83–116)	66.8–188.3
Completed intervals per session (# of intervals)	9.2 ± 2.3	4.9–12.5
High intensity intervals at 80–90% of HRR (%)	25.5 ± 17.8	8.2–53.2
High intensity intervals >90% of HRR (%)	43.3 ± 30.4	1.2–84.3
High intensity intervals <80% of HRR (%)	30.9 ± 35.0	1.8–90.6
Low intensity intervals at 50–60% of HRR (%)	33.0 ± 15.7	5.3–57.2
Low intensity intervals >60% of HRR (%)	43.6 ± 23.7	20.7–91.2
Low intensity intervals <50% of HRR (%)	23.4 ± 12.9	3.5–43.5
Average high interval maximum heart rate (BPM)	131 ± 11	118–151
Mean intensity of high intervals (% HRR)	90 ± 21	59–124

HIIT: high intensity interval training; HRR: heart rate reserve; BPM: beats per minute

Average maximum HR for the high-intensity intervals was 90 ± 21% of HRR with seven of the nine participants averaging a maximum HR that met or exceeded the prescribed intensity and two participants averaging lower than prescribed intensity. Among the nine participants who adhered to the program, baseline VO_2peak_ for men and women were 15.9 ± 3.0 mL/kg/min and 15.0 ± 3.1 mL/kg/min, respectively, which are lower than previously reported recommendations for independent living for older men (≤ 17.7 mL/kg/min) and women (15.4 mL/kg/min) [33], demonstrating the feasibility of HIIT in patients with very low cardiorespiratory fitness.

### Adverse events and modifications to the program

There were no adverse events related to HIIT. One participant experienced abnormally high resting HRs related to a history of atrial fibrillation during one week of the study, but was cleared to continue exercising by his physician. Five of the nine participants reported missing an average of 4.2 ± 1.8 training sessions throughout the study due to cancer- and/or treatment-related effects; these reasons included fatigue from chemotherapy treatment (n = 4; 2.0 ± 0.8 missed sessions), hospital admission (n = 3; 1.3 ± 0.6 missed sessions), pain (n = 1; 3 missed sessions), and a blood clot (n = 1; 6 missed sessions). These five participants completed 2.4 ± 0.9 make-up sessions in subsequent weeks to account for those missed sessions. The program was well tolerated, with modifications made for only one participant who self-selected longer interval durations due to challenges with HR recovery during the low intensity intervals.

### Secondary outcomes: Changes in cardiorespiratory fitness and physical function

The baseline functional assessments were scheduled during clinical visits within three weeks of the start of the intervention (mean time between assessments and start of intervention: 15 ± 9 days). The CPET and introductory training session were completed on the same day, just prior to the start of the intervention (mean time between CPET/introductory session and start of intervention: 2 ± 1 days). Post assessments were completed on all patients regardless of adherence to the study protocol. Post CPET assessments were completed at the time of the sign-off appointment for HCT (prior to transplant) and functional assessment coincided with the closest clinical visit prior to sign-off. Mean time between the last training session and sign-off CPET and functional assessments were 4 ± 2 days and 0 ± 10 days, respectively. Of the ten participants who underwent HCT, seven completed the post CPET and nine completed functional assessments. Reasons for not completing the post CPET assessment was the start of the COVID-19 pandemic (n = 2) and fatigue (n = 1). One participant did not complete the functional assessments due to time constraints from clinical appointments. Among all participants who completed post assessments, there was a significant improvement in VO_2peak_ from baseline to sign-off (14.6 ± 3.1 to 17.9± 3.3; p<0.001; Table 3).

**Table 3:** Changes in cardiorespiratory fitness and physical function.

**All participants who completed baseline and sign-off assessments (n = 9)**			
**Variable**	**Baseline**	**Sign-off**	**P value**
VO_2peak_ (mL/kg/min)^a^	14.6 ± 3.1	17.9 ± 3.3	<0.001*
SPPB (points)	11.9 ± 0.3	11.8 ± 0.4	0.594
Balance (points)	4.0 ± 0.0	4.0 ± 0.0	-
Gait speed (m/sec)	1.3 ± 0.3	1.2 ± 0.1	0.632
Five repeated chair stand time (sec)	9.6 ± 1.4	8.6 ± 2.9	0.387
30-second sit to stand (reps)	14.8 ± 3.0	17.2 ± 4.7	0.122
Six-minute walk test (m)	493.9 ± 67.2	530.9 ± 55.8	0.089
**Participants who adhered to the intervention and completed sign-off assessments (n = 6)**			
**Variable**	**Baseline**	**Sign-off**	**P value**
VO_2peak_ (mL/kg/min)	14.9 ± 3.3	18.0 ± 3.6	0.002*
SPPB (points)	11.8 ± 0.4	12.0 ± 0.0	0.363
Balance (points)	4.0 ± 0.0	4.0 ± 0.0	-
Gait speed (m/sec)	1.3 ± 0.3	1.2 ± 0.1	0.956
Five repeated chair stand time (sec)	10.0 ± 1.0	7.6 ± 1.6	0.019*
30-second sit to stand (reps)	13.8 ± 1.5	18.3 ± 3.3	0.017*
Six-minute walk test (m)	514.4 ± 43.2	564.6 ± 19.3	0.080

Values are means ± standard deviations. VO_2peak_: Peak oxygen consumption; SPPB: Short Physical Performance Battery; Reps: repetitions

^a^n = 7

*p < 0.05, significant difference from baseline to sign-off assessment

There were no changes in SPPB total score, individual SPPB components (balance, gait speed, or repeated five chair stand time), 30-second sit to stand, or 6MWT distance. In a secondary analysis that included only the baseline and sign-off scores for the patients who participated in the training program for the duration of the study and underwent HCT (n = 6), VO_2peak_, 30-second sit to stand, and five repeated chair stand time significantly improved from baseline to sign-off (Table 3). Among those six participants, VO_2peak_ increased by a mean of 3.1 mL/kg/min, 30-second sit to stand improved by 4.5 repetitions, and time to complete five chair stands decreased by 2.4 seconds. Although not significant, 6MWT distance improved by 50.1 meters. In the participants who did not participate/report sessions (n = 3), 30-second sit to stand decreased by 1.7 repetitions and 6MWT distance increased by 10.9 meters. Of those three participants, only one completed the post CPET assessment, with an increase in VO_2peak_ of 4.1 mL/kg/min.

**Fig 4:**
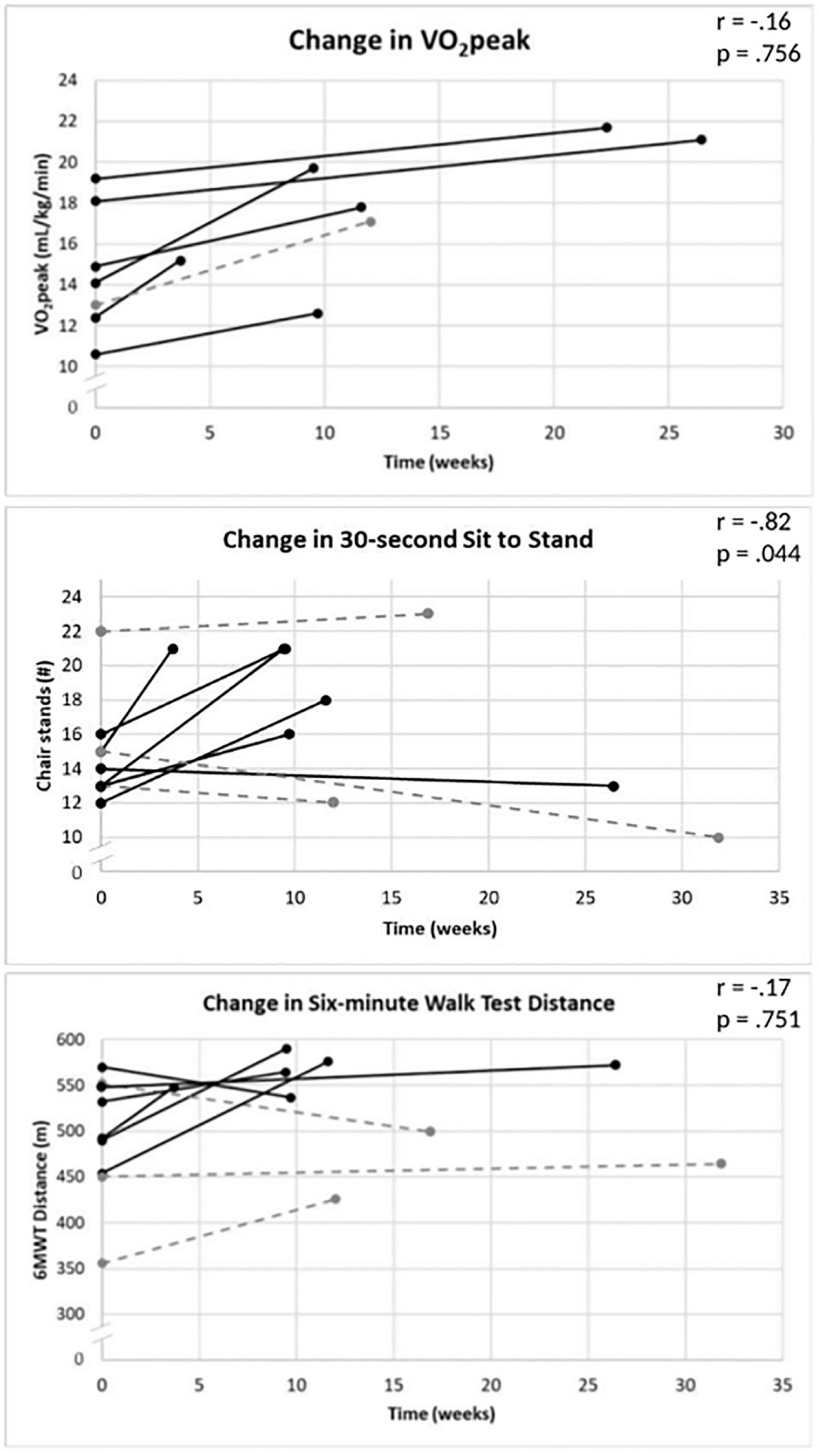
Change in cardiorespiratory fitness and physical function measures for individual participants from baseline to sign-off. Each line represents an individual participant. Participants who adhered to the intervention protocol are presented in solid black lines. Participants who did not adhere to the intervention are presented in dashed gray lines. The r value and p value represent the correlation and its significance, respectively, between intervention length and outcomes for participants who adhered to the intervention.

Fig 4 shows changes in VO_2peak_, 6MWT distance, and 30-second sit to stand measures for each individual participant from baseline to sign-off based on intervention length. There were no significant associations between intervention length (in weeks) and change in cardiorespiratory fitness, SPPB scores or individual components, or 6MWT. Among participants who participated/reported session, there was a significant negative correlation between intervention length and changes in 30-second sit to stand scores (r = -.82, (p = 0.044)) and the relationship between intervention length and time for both the five repeated chair stands and gait speed from the SPPB were approaching significance (five chair stands: r = -.80 (p = 0.057); gait speed: r = -.77, (p = 0.075)).

## Discussion

To our knowledge, this is the first study to demonstrate the feasibility of implementing a remotely monitored HIIT program in patients prior to HCT. Although the feasibility and effectiveness of implementing a pre-HCT home-based interval training program has been previously investigated [23, 24], the research is limited to a small number of published studies. Our findings add to the existing literature by providing remotely monitored HR data that was continuously recorded throughout the training sessions via the Apple Watch to assess adherence to the training sessions and exercise prescription. Among the 24 patients approached by researchers regarding participation in the study, 14 consented to participate. Given that a recent systematic review reported a median recruitment rate of 38% among exercise intervention trials in cancer patients [28], we consider a recruitment rate of 58% as promising, especially in HCT patients who may experience additional barriers to exercise compared to other cancer populations. Therefore, our recruitment rate supports the feasibility of recruiting and implementing a HIIT intervention in this population and its clinical relevance to patients.

Although only nine of the 13 participants who were prescribed the HIIT intervention consistently completed and reported their training sessions, data from those participants demonstrate that a home-based HIIT program is feasible in this population in regard to patients’ ability to complete three HIIT sessions/week, achieve intensities close to maximal levels of exertion during the high-intensity intervals, and sustain multiple intervals over time. Adherence to the number of training sessions and session duration among the nine participants was high, with five participants averaging ≥ 3 sessions/week while completing an average of 9.2 of the prescribed 10 intervals per session (interval adherence of 92%). Session adherence for all participants who were enrolled in the study, including those who did not log any sessions, was 73 ± 53%, which still exceeded previously reported exercise adherence reported in this population [24]. Participants met or exceeded the high-intensity HR prescription on 68.8 ± 34.8% of their intervals with the average maximum HR during the high-intensity intervals reaching 90 ± 21% of HRR. This demonstrates that the nine patients who participated in the intervention were able to sustain repeated intervals at an intensity that was at or above 80% of HRR during the training sessions. While these findings suggest that HIIT is a realistic and achievable mode of training for some patients, the lack of participation by four participants may also suggest that HIIT may not be a viable option for all patients due to factors that may include higher body weight, high fatigue, or functional impairments.

Although adherence was high among the nine participants who took part in the intervention and logged/reported their training sessions, three participants did not report any of their sessions or wear the Apple Watch during the majority of the intervention period and one participant only reported the first eight of 32 weeks. Reasons for the lack of exercise participation in these participants is unknown as participants did not respond to repeated attempts by the exercise physiologist to contact them about their training sessions. Although completion may be related to response to exercise, the data retrieved from two participants who did not report sessions, but wore the watch for a several days at the beginning of the intervention, indicate that no HIIT sessions were attempted. For the participant who reported only the first eight weeks of the intervention, data were not able to be retrieved, which may have impacted continued participation for this patient as the study team was unable to provide feedback and coaching based on the HR data for his HIIT sessions. Given that participation in the intervention was mostly dichotomous with either high adherence among patients (69.2%) or little to no participation at all (30.8%), future research is needed to determine factors that predict exercise participation and strategies to promote exercise adherence during the pre-HCT period. We did identify BMI as a potential factor, which suggests that higher bodyweight may be a barrier to participation in HIIT protocols in this population. In addition, HCT recipients experience health concerns, high infection burden, and barriers to exercise that are unique compared to other cancer and chronic disease populations [34], which may negatively impact response to exercise and exercise participation in this population. Therefore, lower retention rates would be expected in this population. Furthermore, 10 participants who were initially approached about the study declined enrollment, citing reasons that included too much work required by the trial, limited time, fatigue, and distance from the facility. Although the study was designed to address specific barriers including time constraints and travel to the facility, more strategies related to addressing these specific concerns, improving retention, and focusing on barriers specific to HCT recipients may be needed in future trials to improve recruitment and retention. Although we determined that HIIT can be safely performed in this population, it may not be feasible, safe, or appealing for all HCT recipients, and thus warranting more research focused on comparing recruitment, retention, safety, adherence, and efficacy of different types of interventions in this population.

Another challenge with implementation of a pre-HCT intervention is the enrollment of patients who may not eventually proceed to HCT. In this current study, three patients did not undergo transplant due to cancer relapse. While their data were included in the feasibility analysis, they did not complete sign-off assessments since they did not proceed to HCT, thus limiting pre- and post-intervention data and generalizability of results to patients who will ultimately undergo HCT. The expected attrition during the pre-HCT period may pose limitations in future trials, especially for those examining the effect of pre-HCT interventions on post-HCT outcomes, and thus both dropout and exclusion of participants who do not ultimately undergo HCT should be considered when determining sample size.

Heart rate data from the training sessions were successfully retrieved from 72.5% of reported sessions, demonstrating that the Apple Watch along with an mHealth App can be practical tools for remotely monitoring aerobic training and HIIT. Heart rate data during the sessions were retrieved approximately every five seconds, allowing for a comprehensive assessment of HR for the entire HIIT session. Graphs for each session allowed for analysis of HR data to determine the number of intervals completed and how many intervals met the target HR zones. Although we demonstrate that using the Apple Watch with an mHealth App is feasible for remotely monitoring HIIT sessions, we did have issues with missing HR data for 27.5% of reported sessions. This occurred mainly from improper positioning of the watch on participants’ wrist, participants forgetting to wear the Apple Watch, and issues with HR data transferring from the watch into the database. Therefore, to minimize loss of data, strategies including training participants on how to use the Apple Watch, ensuring the wrist band is the appropriate size for the participants’ wrist, frequent check-ins with participants to ensure they are wearing the watch, consistent troubleshooting when data fail to transfer to the database, and making improvements in application and database performance to ensure data are consistently transferred.

Despite some of the technical challenges related to the Apple Watch and the retrieval of the data, patients persevered in working with the platform and the research team with 9 of the 13 participants continuing to exercise until transplant or until it was determined that their disease relapsed and they were no longer eligible for transplant. Furthermore, all patients who participated in the program and were evaluable experienced improvements in cardiorespiratory fitness and performance on the chair stand tests and 6MWT. This suggests that despite technical challenges, patients participated and improved their function, supporting feasibility of a remotely monitored home-based HIIT intervention.

Although our small sample size limits us in regard to statistical analysis and power, our preliminary analysis of changes in cardiorespiratory fitness and physical function suggest that a pre-HCT HIIT program has beneficial effects on cardiorespiratory fitness. In those who participated in the intervention, VO_2peak_ significantly improved from baseline to sign-off, with an average improvement of 3.1 mL/kg/min (21%). This is consistent with findings from Wood et al. [24] who reported a 3.7 mL/kg/min increase in VO_2peak_ following a prehabilitative interval training program in HCT recipients. We also observed an improvement in VO_2peak_ in one participant who did not adhere to the intervention protocol. Since this participant did not report his training sessions or wear the Apple Watch, we do not know if he completed any exercise sessions or increased his overall physical activity since the start of the study, thus limiting our ability to draw conclusions from his data. Considering it has been previously shown that every one MET (3.5 mL/kg/min) increase in exercise capacity is associated with a 12 percent improvement in survival [35], our results may be especially relevant for this population to improve survival following HCT. Furthermore, Wood et al. [13], found that a pre-HCT VO_2peak_ ≤ 16 mL/kg/min was associated with a longer hospital stay during initial transplant, a higher number of hospitalized days before Day 100, and a higher risk for mortality compared to patients with a VO_2peak_ > 16 mL/kg/min. Five of the seven participants in our study who completed the baseline and post CPET started off with a VO_2_peak that was less than 16 mL/kg/min. Following the intervention period, three of the five participants had increased their VO_2peak_ to above 16 mL/kg/min, suggesting that HIIT may result in cardiorespiratory improvements with a potential benefit on clinical outcomes. In addition, there was no relationship between changes in VO_2peak_ and intervention length with comparable VO_2peak_ increases observed among patients with shorter intervention length and longer intervention length. This suggests that even a short HIIT intervention period prior to HCT may be enough to gain cardiorespiratory improvements.

Among adherent participants, scores for the 30-second sit to stand and five repeated chair stand time significantly improved, which suggests that HIIT was beneficial for leg strength and power. While clinically meaningful changes have not been established in HCT recipients, the 4.5 repetition increase in 30-second sit to stand exceeds minimal clinically important improvements established in other chronic disease populations [36, 37]. Likewise, the mean improvement of 50.1 meters in 6MWT distance is greater than the 30.5 meter improvement that has been considered clinically meaningful in other populations [38] and it corresponds to a 9% reduced risk for mortality [15]. Considering the changes in both the 30-second sit to stand and 6MWT coincided with clinically meaningful functional changes for other chronic disease populations, our results are promising in that a remotely monitored HIIT program may be a potential strategy for improving physical function in patients prior to undergoing HCT. We observed a significant negative association between 30-second sit to stand and intervention length. This suggests that improvements in physical function can be achieved in a short period of time, with the greatest improvements occurring in individuals with an intervention period that was less than 10 weeks. In addition, the diminished returns that occurred with a longer intervention may indicate that progressions such as increased frequency, intensity, or interval number and length may be needed as participants become more fit to provide sufficient overload for continued improvement [39]. Larger studies using a randomized controlled design are needed to further evaluate the effects of a pre-HCT remotely monitored HIIT intervention on physical function.

A strength of this study includes the individualized HIIT program that was based on resting HR and maximum HR achieved at VO_2peak_ during the CPET to tailor the program to each individual participant. Another strength is the use of mHealth technology to motivate participants, monitor training intensity, and assess the feasibility of the intervention. Since a home-based training program is a safer and more practical option for this population, the use of technology allowed researchers to remotely monitor the participants’ exercise sessions in real time to provide feedback on HR values and assess adherence to the HIIT prescription. Therefore, findings regarding feasibility for this study can be used to inform future trials on the use of technology for assessing exercise program intensity, adherence, and fidelity of home-based interventions.

Limitations to this pilot study include the non-randomized design, small sample size, and a small number of participants who logged/reported their training sessions and wore the Apple Watch for analysis. In addition, of the 24 participants who were screened for eligibility and approached for the study, 18 were male and 6 were female, with 10 males and 3 females ultimately participating in the study. Given that the majority of patients who were approached and participated in the study were male, results may be less generalizable to female patients. We also did not obtain information regarding prior exercise experience or other activities participants completed outside of the HIIT sessions, which may have influenced exercise adherence and results. The start of the COVID-19 pandemic also limited recruitment and prevented the completion of sign-off CPETs for two participants and three participants never underwent HCT. As a result, we were limited in our approaches for data analysis and ability to interpret the data. Furthermore, data were not retrieved on 27.5% of sessions, which limited our ability to analyze data from those HIIT sessions. Due to the technical challenges in obtaining some of the exercise HR data from the Apple Watch, we also cannot be sure that data are missing at random. Given that this was the first trial where we tested the utility of the Apple Watch and the mHealth Apps to collect the HIIT intervention data, we encountered issues early on in collecting and retrieving the Apple Watch data that we were able to resolve more quickly as the trial went on, thus improving our ability to collect and retrieve the data. Thus, we anticipate a lower percentage of missing data in future trials. Since it has been previously suggested that <5% of missing data is acceptable while >20% may pose threats to validity [40], more research is needed to determine how retrieval of exercise data can be improved. Another limitation was that the duration of the intervention varied among participants. Because the exercise program was a pre-HCT intervention, the number of weeks patients participated in the training intervention was dependent on when their HCT was scheduled, and thus the time between baseline and post assessments was different for each patient; unfortunately, due to the nature of the pre-HCT period and variability in time to HCT due to clinical factors, this is something that cannot be controlled. While this is an ongoing challenge for this type of intervention, our preliminary data show that VO_2peak_ and physical function improvements could be obtained in those who participated in the intervention for a shorter length of time.

Although findings demonstrate that participants were able to perform HIIT with high adherence to the exercise prescription, it is also important to acknowledge that there is heterogeneity among the HCT population and thus a standardized remotely monitored program like HIIT may not be realistic for all patients. Although the HIIT prescription was individualized to each patient and the mode was self-selected, other types of exercise programs that begin with lower intensities as well as the use of additional behavior change strategies may be more practical options for patients who may be limited by factors such as high fatigue, obesity, and/or functional impairments. Long-term sustainability, affordability, and accessibility of this intervention should also be considered as trained staff is needed to implement the intervention and provide motivation, costs associated with the Apple Watch and iPhone, and necessity of WiFi access so that Apple Watch data can be transferred to researchers. Future research should explore options for automated tracking of HIIT intervals and feedback and cost-effective strategies for implementation.

## Conclusions

Findings from our study demonstrate that implementing a home-based remotely monitored HIIT program in patients prior to undergoing HCT is feasible. In addition, we demonstrated that mHealth technology can be used to remotely monitor exercise adherence and training intensity. Although analysis of changes in cardiopulmonary and physical function was limited due to the small sample size, our preliminary findings support the need for larger randomized control trials to determine if HIIT can be an effective approach for improving these outcomes prior to HCT. Future studies are also needed to determine barriers to participation in a remotely monitored exercise intervention and strategies for promoting retention and exercise adherence in this population. Furthermore, future development and improvements of phone applications and databases to transfer Apple Watch data to the research database are needed to ensure HIIT session data can be retrieved by the research team, which will improve interpretation of results and provide a better indication of the Apple Watch’s ability to consistently capture HR data during HIIT.

## Supporting information

S1 File Consort checklist. (PDF)

S2 File IRB protocol. (PDF)

S3 File Data set. (XLSX)
