# Supplementary material for: A pilot study to assess the feasibility of a remotely monitored high-intensity interval training program prior to allogeneic hematopoietic stem cell transplantation
Source: PLoS One. 2023 Nov 30;18(11):e0293171. doi: 10.1371/journal.pone.0293171 (PMC10688680; doi:10.1371/journal.pone.0293171)
Supplement: S2 File — (PDF) [file pone.0293171.s002.pdf]

A Research Program Targeting Pre-, Peri-, and Post-transplant Optimization Program (R-POP)

Sponsor: Anthony Sung, MD

Funding Source: Hematologic Malignancies and Cellular Therapy Department/Division

Protocol Source: Anthony Sung, MD

Duke IRB#: Pro00092963

Principal Investigator	Sub-Investigators	Statisticians
Anthony Sung, MD<br>Box 3961<br>Duke University Medical Center<br>Durham, NC 27710<br>Phone: 919-668-5710<br>Fax: 919-668-1091<br>Anthony.sung@duke.edu	Nelson Chao, MD<br>Taewoong Choi, MD<br>Cristiana A. Costa-Chase, DO<br>Cristina Gasparetto, MD<br>Mary Hamlin, MSW<br>Mitchell Horwitz, MD<br>Yubin Kang, MD<br>Sarah Kelleher, PhD<br>Michel Khouri, MD<br>Pao-Hwa Lin, PhD<br>Gwynn Long, MD<br>Richard Lopez, MD<br>Miriam Morey, PhD<br>Amy Pastva, PhD<br>Elena Perea, MD<br>Katy Peters, MD<br>Shelby Reed, PhD, RPh<br>Stefanie Sarantopolous, MD, PhD<br>Nirmish Shah, MD<br>Rebecca Shelby, PhD<br>Sophia Smith, PhD, MSW<br>Tamara Somers, PhD<br>Paul Wischmeyer, MD<br>Syed Yousuf Zafar, M.D<br>Jeroen Molinger, MS	Sin-ho Jung, PhD<br>Yi Ren, MS<br>Zhiguo Li, PhD<br>Steven Grambow<br><br>**Lead Study Coordinator**<br><br>Krista Nichols, RN, MSN, AOCNS<br>krista.rowe@duke.edu<br><br>**Regulatory Coordinator**<br><br>Lauren Hill<br>lauren.bohannon@duke.edu

Table of Contents

Table of Contents 2
1.0 OBJECTIVES 4
1.1 Primary Objective: 4
1.2 Secondary Objectives: 4
2.0 BACKGROUND, RATIONALE 4
2.1 Physical Activity and Function 5
2.2 Cognitive Function 5
2.3 Mental Health 5
2.4 Nutrition and Diet 5
2.5 Social Support 6
2.6 Caregiver Support 6
2.7 Microbiota Diversity 6
2.8 Biomarkers of Inflammation and Frailty 6
2.9 Summary 7
3.0 PATIENT AND CAREGIVER ELIGIBILITY 7
3.1 Patient Inclusion criteria 7
3.2 Patient Exclusion criteria 7
3.3 Caregiver Inclusion Criteria 8
3.4 Caregiver Exclusion Criteria 8
3.5 Subject Recruitment, Selection, and Consent 8
4.0 STUDY PROCEDURES AND ENDPOINTS 9
4.1 Physical Activity and Function 11
4.2 Cognitive Function 12
4.3 Physical and Cognitive function, Mental health, and Social support 14
4.4 Nutrition and Diet 15
4.5 Applications 17
4.6 Caregiver Support 18
4.7 Global Assessments and Symptoms 19
4.8 Safety Endpoints 22
5.0 ANTICIPATED TOXICITIES 23
6.0 STATISTICAL CONSIDERATIONS 23
6.1 Analysis of the Microbiota: 24
6.2 Power and sample size calculations: 24
7.0 DATA SAFETY MONITORING PLAN 24
7.1 Adverse Events Reporting 32

7.2 Serious Adverse Events (SAE) 33
7.3 Reporting of SAEs 33
7.4 Quality Control and Quality Assurance 33
7.5 Audits 34
8.0 DATA STORAGE AND CONFIDENTIALITY 35
8.1 Mobile Application and Duke-Loaned Device Confidentiality 35
8.2 Records Retention 37
9.0 DATA MANAGEMENT AND PROCESSING 38
9.1 Study Documentation 38
9.2 Case Report Forms (CRFs) 38
9.3 Data Management Procedures and Data Verification 39
9.4 Coding 39
9.5 Study Closure 39
10.0 ADMINISTRATIVE AND ETHICAL CONSIDERATIONS 38
10.1 Regulatory and Ethical Compliance 38
10.2 DUHS Institutional Review Board and DCI Cancer Protocol Committee 38
10.3 Informed Consent 38
10.4 Protocol Amendments 38
11.0 REFERENCES 39
12.0 APPENDICES 49
12.1 APPENDIX A: PATIENT REPORTED OUTCOME INSTRUMENTS 49
12.2 APPENDIX B: Hand Grip Testing 48
12.3 APPENDIX C: NON-ENROLLMENT SURVEY (Decline Survey) 54
12.4 APPENDIX D: Patient Reported BMT Questionnaire 55

1.0 OBJECTIVES

1.1 Primary Objective:

"To evaluate the feasibility and outcomes of a research pre-, peri-, and post-transplant optimization program (R-POP) to improve multiple domains of health including physical function, cognitive function, mental health, and diet and nutrition for patients planning to undergo or undergoing hematopoietic stem cell transplantation (HCT)"

1.2 Secondary Objectives:

1.2.1 Gather information on how the following parameters affect hematopoietic stem cell transplant patients' outcomes and wellness/quality of life:

- physical function and activity,
- cognitive function,
- mental health,
- diet and nutrition,
- social support,
- caregiver support,
- microbiota diversity, and
- biomarkers of inflammation and frailty.

1.2.2 Compare additional clinical endpoints to include:

- overall survival,
- disease-free survival,
- rates of bacterial, fungal, and viral infections as well as overall infections,
- rates of hospital and intensive care unit admission/re-admission and length of stay,
- graft-versus-host disease
- transplant length of stay,
- return to work,
- falls,
- delirium,
- quality of life

1.2.3 Compare caregiver quality of life

2.0 BACKGROUND, RATIONALE

Hematopoietic stem cell transplantation (HCT) is a complex medical treatment that causes significant symptom burden for patients and their caregivers. Patients receive chemotherapy and/or radiation therapy prior to transplant to eradicate the underlying disease and provide immunosuppression. While the myelosuppressive or myeloablative conditioning regimens used prior to transplant provide medical support to treat underlying disease and provide immunosuppression, they can cause the development of physical,[1] psychological,[2] and nutritional challenges[3-5] that can impact a patient's short- and long-term wellness. Allogenic HCT is associated with treatment related mortality (TRM) ranging from 20-30%.[6] TRM is particularly high in patients with advanced age, decreased functional status, and/or comorbid conditions such as depression or organ dysfunction. While evaluation of organ dysfunction is required part of pre-transplant workup (echocardiogram, pulmonary function testing, etc.), decreased physical function, cognitive deficits, depression, poor nutrition, and lack of

social support are less often evaluated. This investigation will assess these five domains and implement interventions to improve them.

2.1 Physical Activity and Function

The inability to perform normal physical functions and activities resulting from intensive preparatory chemotherapy for hematological malignancies is known to have an impact on quality of life.[7] Furthermore, the weight loss commonly seen in cancer patients (i.e. cancer cachexia) comes with skeletal muscle mass deterioration and declines in strength and function that are associated with morbidities like fatigue and treatment tolerance and mortality.[8] Exercise has the potential to maintain or increase muscle mass and may be an effective treatment to prevent the psychological and physiological consequences of physical inactivity. While some works have looked into how post-HCT exercise programs affect patient outcomes,[9] there is limited research on the effects of an exercise intervention in patients prior to HCT. Some studies have shown physical and emotional benefits for those who have completed such interventions;[10-13] however, many of the studies intervened at hospital admission,[10-12,14,15] (as opposed to the three month period prior to HCT start). One randomized clinical trial examined how a semi-supervised, self-administered exercise program prior to, during, and after allogenic HCT influenced physical functioning, fatigue, distress, and quality of life.[16] All of the 52 patients randomized to the exercise intervention experienced declines in distress and fatigue and improvements in physical functioning.

2.2 Cognitive Function

Cognitive impairments include declines in mental processing speed, recall deficiencies, and trouble concentrating. A recent trial found that frail, cognitively impaired adults have a lower survival rate than those without frailty and cognitive deficiencies.[17] Similarly, prostate, breast, and colorectal cancer patients with cognitive declines were observed to have a shorter life expectancy than those without any cognitive deficiencies.[18] Chemotherapy-related cognitive impairment, more recently and commonly called “chemobrain,” is a phenomenon reported by non-central nervous system cancer patients for decades.[19] While the majority of research has been in women with breast cancer,[20] studies in other types of cancers show similar cognitive disruptions[21,22] even HCT.[23-27] In one group of patients undergoing HCT, short-term memory impairment doubled during hospitalization and the deficit remained in almost 20% of participants at 8-month follow-up.[26] In another study, over one-quarter of HCT patient survivors who underwent cognitive performance evaluations were found to have at least moderate impairment, and 50% had mild impairment.[28] We propose to examine if identifying and intervening for cognitive impairment prior to HCT will improve functional status and overall outcomes of patient participants who receive a HCT at the Duke Bone Marrow Transplant Clinic.

2.3 Mental Health

In addition to cognitive impairments, pre-transplant emotional status and mental health can also influence quality of life[29] and outcomes post-transplant.[26,30,31] In a study of one hundred HCT patients the greatest emotional distress was found to occur prior to transplantation,[32] highlighting the need for pre-transplant interventions to abate negative outcomes associated with poor psychological health. More specifically, the presence of depression pre-HCT was related to increased incidence of acute graft-versus-host-disease, hospital stay, and overall survival.[33] Thus, a program not only identifying, but also treating, monitoring, and following-up with pre-HCT cognitive deficits and psychological triggers is necessary to help improve HCT survivors’ wellness.

2.4 Nutrition and Diet

The conditioning phase prior to HCT can be nutritionally taxing for individuals and those who are identified as at risk for malnutrition prior to HCT may be more likely to experience malnutrition post-transplant.[34] Moreover, hospital length of stay can be higher in those who are identified as malnourished two weeks prior to

HCT.[35] A recent study examining the nutritional status of 100 individuals undergoing HCT found that nutrition-related biochemical parameters declined significantly following transplant, getting progressively lower over time even though body mass remained normal.[36] In another trial, 30% of HCT patients reported having a nutritional impact symptom on the patient-generated subjective global assessment; although, almost 90% did not identify as having any troubles eating.[35] These results suggest that early interventions, specifically those containing diet education prior to transplant, may curb the nutritional consequences seen during and following HCT, which can improve long-term health and quality of life.

2.5 Social Support

HCT has been described as psychosocially taxing, causing core, personal vulnerabilities to surface.[37] Research shows that perceptions of adequate social support improves quality of life[29] and emotional[31,38] and physical[39] health post-HCT. More specifically, greater social support has been associated with lower PTSD symptoms and incidence of depression and higher quality of life and survival rates.[38,40-44]

2.6 Caregiver Support

Caregivers play a critical role in the HCT patient's disease management and outcomes.[45] They provide care to HCT patients regardless of time or day, carrying responsibilities like providing transportation for patients to and from daily treatments, purchasing and preparing food/meals, and managing patient medications, duties that can sometimes prevent them from engaging in their own health-promoting behaviors[46,47] and leisure activities.[48] Caregivers of HCT patients face challenges emotionally, physically, and financially and can experience sleep disturbances and distress more than individuals who do not care for persons with such a medical condition.[47] The HCT patient's emotional and physical health were identified as the most challenging aspects of the caregiver's role.[49] The authors of a recent review investigating the burden on caregivers of HCT patients concluded that the pre-transplant period is an optimal time to evaluate caregiver distress.[50] An early assessment may provide adequate time to intervene and reduce stress, anxiety, and depression, similar to what Laudenslager, et al.'s RCT found,[51] although their trial was initiated 2 weeks post-transplant.

2.7 Microbiota Diversity

The intestinal microbiome has many roles, including immune function support, digestion of food, colonization resistance, and metabolism regulation.[52-54] Disruption of microbiota stability may be associated with conditions like diabetes, allergies, inflammatory bowel disease, and autoimmune disease.[52,53,55] Evidence comparing pre- and post-transplant fecal samples shows that there is substantial intestinal bacterial translocation,[55-60] which disrupts the microbiome-created intestinal barrier, resulting in a loss of microbiota diversity, reduction in the originally predominant microorganism, and establishment of a new bacterial group. Unfortunately, this shift is thought to increase the risk of post-transplant infection because of immunosuppression.[59,61] More specifically, this loss of bacterial diversity, also known as dysbiosis, affects morbidity and mortality following HCT by increasing the risk for graft-versus-host disease and infection.[59,62,63]

2.8 Biomarkers of Inflammation and Frailty

The preparative regime for HCT results in activation of the inflammation cascade in-vivo. This inflammatory response may put the recipient's (or host's) tissues at an increased risk for an attack by the donor's immune cells. Acute graft-versus-host disease (GVHD), a condition known to affect up to 60% of HCT patients and a large contributor to treatment-related mortality and morbidity,[64] occurs when a host's tissues are harmed by the donor's immune cells, resulting in damage to the liver, gastrointestinal tract, and skin. Four biomarkers, interleukin 8 (IL-8), hepatocyte growth factor (HGF), tumor necrosis factor receptor 1 (TNFR1), and interleukin-2 receptor alpha (IL-2Rα), have been identified to accurately distinguish patients with and without acute GVHD.[65]

Oxidative stress, the disproportion between the antioxidant defenses and free radical production within the body, has been associated with multiple chronic diseases and conditions, including cancer and frailty. In a large trial (N=742) of older adults, frail persons had higher levels of protein carbonylation, a marker of oxidative stress,[66] versus those not considered frail. [67] In mice, tumor-necrosis factor-alpha (TNF-α) was found to play an integral role in cognitive decline and neuroinflammation, with its inhibition reducing such outcomes via limiting IL-1 release.[68] Chemotherapy-induced oxidative stress can impact the heart and brain[69] and may hinder the effectiveness of antineoplastic agents' ability to destroy cancerous cells.[70] HCT patients may experience high oxidative stress due to conditioning prior to transplantation. In a recent study of 32 HCT patients, oxidative stress, as measured by urinary 8-hydroxydeoxyguanosine (8-OHdG), was 5 times higher than before conditioning.[71]

Delirium may be the result of the inflammatory response and/or variations in neurotransmission and brain blood flow.[72] It is associated with poor outcomes, such as increased length of hospital stay, and mortality.[73] Approximately 26-44% of advanced cancer patients experience delirium[74,75] and the cause is thought to be multifactorial.[76] Fifty percent of HCT patients in one trial were evaluated and found to have delirium.[77] In this trial, patients were followed from 1 week before to 30 days after transplantation. Factors such as poor physical functioning and decreased cognitive performance were observed to be pretransplant risk factors for a delirium episode.[77] Identifying those at risk for delirium using biomarkers can help avoid negative outcomes. Grandi and colleagues found higher levels of neuron-specific enolase (NSE), potentially a marker of blood-brain barrier permeability, and brain-derived neurotrophic factor (BDNF), a neurotrophin that has roles in neurogenesis, pain perception, and depression,[78] in ICU patients who develop delirium.[79] Elevated levels of other cytokines, such as IL-2 and IL-6, have been identified in those with delirium as well.[80]

2.9 Summary

There is the potential to improve these areas during the 2-3 month window between new patient evaluation and HCT. Much of the literature has focused on improving health indicators during the transplant process to support better outcomes post-HCT. However, the question we want to answer is: can patients improve their health and function pre-HCT and, thus, improve outcomes post-HCT? We are aware of only one intervention taking a similar, but not identical approach.[81] Uniquely, we will target patient participants at their initial evaluation, prior to hospital admission, and follow them throughout the transplant process to discharge, providing them with consistent, evidenced-based physical activity, diet, cognitive, social, and emotional interventions. Our hope is to improve patient outcomes and quality of life by intervening prior to HCT.

3.0 PATIENT AND CAREGIVER ELIGIBILITY

3.1 Patient Inclusion criteria

1) Plan to undergo an allogeneic hematopoietic stem cell transplant for any cancer or non-cancer illness within the next 6 months
2) Age 18-80 years

3.2 Patient Exclusion criteria

1) Both patient and caregiver are unable to read and follow directions in English (ok if only patient cannot read and follow directions in English as caregiver will be able to help).
2) Any absolute contraindications to exercise:
   a) recent (< 6 months) acute cardiac event;
   b) unstable angina;
   c) uncontrolled dysrhythmias causing symptoms or hemodynamic compromise;
   d) symptomatic aortic stenosis;

e) uncontrolled symptomatic heart failure;
f) acute pulmonary embolus;
g) acute myocarditis or pericarditis;
h) suspected or known dissecting aneurism; or
i) coronary artery disease.

3) Functional impairment resulting in inability to exercise

In addition, patients who meet these inclusion and exclusion criteria and consent to participate will undergo cardiopulmonary exercise testing (CPET), with heart rate and rhythm will be monitored continuously using a 12-lead ECG. The ECG will be used to make sure the subject does not have any abnormalities before, during, and after the treadmill stress test. Results will be reviewed by a physician before the subject is cleared to participate in interval training. Therefore, a subject may meet inclusion and exclusion criteria, consent to participate, but then not meet cardiology clearance and therefore will be unable to participate in the intervention; these patients and their caregivers will then be transitioned off study.

3.3 Caregiver Inclusion Criteria

1) Identified by patient as their primary caregiver
2) Age 18-80 years

3.4 Caregiver Exclusion Criteria

1) None

3.5 Subject Recruitment, Selection, and Consent

Potential patient subjects will be recruited from the Duke Adult Bone Marrow Transplant (ABMT) Program. Under the current standard of care practice, ABMT physicians evaluate all new patients. If the patient's ABMT physician deems the patient to be an allogeneic transplant candidate and suitable for Research POP (R-POP), and the patient expresses interest in participating, they will be referred to research staff, who will review inclusion/exclusion criteria and tell the patient more about the study. Both the ABMT physician, who will be a sub-investigator, and the research staff will make clear that participation in this trial is voluntary, and patients may decline to hear more or to participate without penalty; these patients will receive standard of care.

Those interested in learning more will meet with study staff who will explain details, answer questions, and confirm inclusion/exclusion criteria. Only subjects who voluntarily provide full informed consent will be included in the study.

Subjects who are screened but do not want to participate (do not give informed consent) will not be recorded and will receive standard care. They will be asked to complete a decline survey (Appendix D) in order to inform the investigators of why participation was declined and improve study design for future interventions.

Caregiver subjects will be identified by their patient as the patient's primary caregiver. They will be approached by study staff who will explain details, answer questions, and confirm inclusion/exclusion criteria. Only those who voluntarily provide full informed consent will proceed to study enrollment. Patients may decide to enroll without their caregivers deciding to enroll; in these cases, patients will participate in the study and caregivers will not participate in any research-related events. Caregivers may not enroll if their patient does not enroll.

This is a single arm feasibility study; all subjects will be assigned to the intervention group. We anticipate 35 patients will be consented and 30 patients to pass CPET/cardiology clearance to receive interval training. Up to 35 caregivers may consent and 30 caregivers participate, though this number may be lower depending on caregiver availability and consent.

4.0 STUDY PROCEDURES AND ENDPOINTS

Those who consent to participate in R-POP will return within one month (preferably within two weeks) for a separate day of assessments (e.g. cardiopulmonary exercise testing), nutrition education (e.g. what constitutes a serving of protein), and training on how to use iOS devices (e.g. iPad, iPhone) and activity trackers (e.g. Apple Watch), which will be provided to patients for their use during the duration of study participation. In their 2011 report the World Health Organization states that, "the use of mobile and wireless technologies to support the achievement of health objectives (mHealth) has the potential to transform the face of health service delivery across the globe,"[82] and this investigation will contribute to solidifying use of a mHealth platform with all Duke HCT patients. iPad or iPhones will be pre-loaded with apps and/or PDFs which may include (1) SplendoFit (2) Technology Recordings to better Understand BMT (TRU-BMT), developed by co-Is Shah and Sung to monitor patient reported outcomes of health, quality of life, and nutrition (Duke IRB Pro00068979), (3) an electronic copy of the Duke ABMT Patient Handbook (this is an electronic version of a paper handbook that is given to all transplant patients as part of standard of care), and (4) an electronic copy of Duke ABMT Recipes for Transplant Patients (this is an electronic copy of paper recipes available to all transplant patients as part of standard of care). (5) Jabber, or a similar program, to facilitate video communication (e.g. for monitoring and support of exercise sessions); (6) recipes for meals and smoothies for HCT prepared by collaborator Lowder (registered dietitian).

Significant challenges of behavioral interventions include monitoring and adherence. By using activity trackers, we will be able to monitor whether patients are meeting their heart rate goal; because data will be available in real-time, research staff can follow up to provide positive feedback to patients who are meeting goals or coaching and assistance to those who are not as successful. Similarly, daily monitoring of dietary intake on the custom HCT app will be supported by wireless transmission and review of the data by a research dietitian, who will give feedback and education to patients on food choices. Our mHealth platform combines existing technology (e.g. Apple Watch, Microsoft Band 2) with custom solutions (HCT app), both of which have already been piloted in Duke HCT patients (IRB Pro00068979, PI Shah, co-I Sung) and which patients view favorably as both easy to use and helpful (abstract accepted for presentation at the American Society of Hematology 2017 Annual Meeting).

The following table outlines the assessments each consenting patients will be asked to complete and the interventions directed at each domain; further details are provided in subsections:

Table 1

Domain	Assessment	Time (min)	Timepoints	Intervention
**Physical Activity and Function**	Short physical performance battery	0*	6T	Interval training
	6 minute walk	0*	6T	
	30 second sit/stand	0*	6T	
	Grip strength	0*	6T	
	Fried	0*	6T	
	PROMIS Physical Function	2	6T	
	CPET	60	NPE, SO, D30****, DC, D180	
	BodPod or BIA	5	NPE, SO, DC, D180, Y1	
	Musclesound	20	6T (D30****)	

**Cognitive Function**	Montreal Cognitive Assessment<br>PROMIS Cognitive Function<br>RBANS<br>Trail Making Test<br>Brief Cope	0*<br>2<br>30<br>5<br>5	6T<br>6T<br>6T<br>6T<br>6T	Interval training
**Mental Health**	PHQ9<br>PC-PTSD/PCL-5<br>PROMIS Depression<br>PROMIS-Anxiety	0*<br>0*<br>2<br>2	6T<br>6T<br>6T<br>6T	
**Nutrition and Diet**	Peri-operative nutrition screen<br>PG-SGA<br>Vit D, Vit A, albumin, prealbumin, lipids, A1c; if PONS+ also thiamine, Zn, Cu, selenium**<br>24 food recall<br>Food Security<br>DHQ3	0*<br>2<br>0*<br>10<br>2<br>30	6T<br>6T<br>6T<br>6T<br>NPE, D180, Y1<br>6T	Daily tracking of food groups
**Social Support**	PROMIS-Emotional Support<br>PROMIS-Social Isolation<br>Lorig Self Efficacy<br>Two Factor Consideration of Future Consequences Scale (CFC-14)<br>Brief Resilience Scale (BRS)	2<br>2<br>2<br>5<br>2	6T<br>6T<br>6T<br>6T<br>6T	
**Symptoms**	NCCN Distress Thermometer	0*	6T	Per clinical care
**Comorbidities**	HCT-CI (completed by MD)	0	NPE	Per clinical care
**Global**	FACT-BMT<br>EQ-5D-5L<br>OARS IADL	0*<br>0*<br>0*	6T<br>6T<br>6T	NA
**Microbiota**	Skin Swab<br>Stool Collection	5<br>5	6T<br>6T + D0, 7, 14, 21, Y2	NA
**Biomarkers**	Blood collection	1	6T + D14	NA
**Financial**	Financial Assessment	0*	NPE, D90, D180	NA
**Activity Data**	Heart rate<br>Step count<br>Activity Intensity<br>Sleep	0***<br>0***<br>0***<br>0***	Continuous	Per discussion as part of interval training
**Applications**	TRU-BMT or SplendoFit	5	Daily thru Year 1	
**Polypharmacy and**	Case Report Form	0*	SO, D30, D90, D180,	NA

**Medication Reconciliation**			Y1	
	Total time (patients):	179		

PROMIS=Patient Reported Outcomes Measurement Information System; 6T=6 main timepoints (pre-transplant: new patient evaluation [NPE] and sign off [SO] prior to transplant; peri-transplant: day 30 [D30], discharge home [DC] (approximately D90); post-transplant: day 180 [D180], year 1 [Y1]); CPET=Cardiopulmonary Exercise Test; PC-PTSD-5=Primary Care PTSD for DSM-5; PG-SGA=scored Patient Generated Subjective Global Assessment; A1c=Hemoglobin A1c; PONS=Perioperative Nutrition Score; PONS+=positive PONS; Zn=zinc; Cu=cooper; FACT-GP=Functional Assessment of Cancer Therapy – General Population; PHQ=Patient Health Questionnaire; RBANS= Repeatable Battery for the Assessment of Neuropsychogical Status; N/A=not applicable
*Completed as part of standard clinical practice, or with cPOP (Pro00111631) or with blood draws being done as part of standard clinical practice; while this data will be analyzed as part of this study, no additional or minimal additional patient time or involvement is required to collect it.
** Per investigator/MD discretion
*** Data will be collected passively from the Apple Watch and no patient involvement is required to collect data.
**** Will only be collected if subject is medically stable.

While the total anticipated time for assessments is 179 minutes, 85 minutes is cardiopulmonary exercise testing, 11 minutes are related to sample collection, and 83 minutes are questionnaire-based; these will be split into two sessions to minimize patient burden.

Use of previously collected data: If a subject is simultaneously on another study and the same assessment or sample is required for both studies, we will only collect once and share the data between studies to avoid subjects having to duplicate efforts.

Collection Windows:
New Patient Evaluation: +30 days of New Patient Evaluation Clinical appointment
Sign Off: -/+ 7 days of Sign Off Clinical appointment
Day 0, Day 7, Day 14, Day 21, Day 30, Day 60: -/+ 7 days
Discharge: -/+ 14 days
Day 180, Year 1: -/+ 30 days

4.1 To discuss each assessment in detail:Physical activity and function

4.1.1 Cardiopulmonary Exercise Test

Exercise capacity will be assessed using a symptom-limited Cardiopulmonary Exercise Test (CPET) on a motorized treadmill with expired gas analysis to determine VO_{2peak}, according to guidelines for clinical populations.[88] Every effort will be made by study staff to ensure that all patients who take beta-blockers will have taken their medication prior to testing. CPET will be administered +/- 2 hours from when patients would be participating in exercise training (see below). All CPET data will be recorded as the highest 30-second value elicited during the CPET. During exercise, heart rate and rhythm will be monitored continuously using a 12-lead ECG. The ECG will be used to make sure the subject does not have any abnormalities before, during, and after the treadmill stress test. This test will last approximately one hour. Additionally, the Patient-Reported Outcomes Measurement Information System (described below) Physical assessment will be completed.

To discuss patient study interventions in more detail:

Prior to beginning the exercise intervention, cardiopulmonary exercise testing (CPET) will be used to both determine the patient's baseline and determine safety and suitability of patient participation.

Specifically, during exercise, heart rate and rhythm will be monitored continuously using a 12-lead ECG. The ECG will be used to make sure the subject does not have any abnormalities before, during, and after the treadmill stress test. Results will be reviewed by a physician before the subject is cleared to participate in interval training.

For the interval training intervention, patients will be asked to complete up to 3 x 30-minute structured high intensity interval training aerobic exercise sessions/week. The structured aerobic exercise will incorporate an intensity specific interval training design with intensities relative to each patient's individual fitness and thus comparable between patients. Heart rates and intensities will be determined from the maximal exercise test (CPET). The preferred mode will be walking, however if the patient prefers swimming or cycling we will adapt the plan accordingly. For the first session, patients will come to the Duke Center for Living for in person evaluation and training. Patients will be asked to wear a chest heart rate strap which will connect with the heart rate monitor. This will ensure accurate heart rate at higher intensity activities. Patients will also wear an Apple Watch and results of heart rate monitoring on the watch will be compared to the chest strap. Before and after each session patients will engage in an active 5-minute warm-up and cool-down at an intensity corresponding to 40%-50% VO_{2peak}. In our experience this is similar to a slow, leisurely walk. Following the warm-up, patients will increase speed to correspond with an intensity of approximately 80% VO_{2peak} for 1-minute before returning to warm-up speed for 1-minute (50% VO_{2peak}). In our experience this is similar to a brisk uphill walk, or climbing stairs. Patients will complete up to 10 of each vigorous intensity (80%) 1-minute interval and 10 of each low intensity (50%) 1-minute interval, or 30-minutes total exercise (including warm-up and cool-down). This type of training has been shown to be safe and feasible in breast and prostate cancer while improving fitness rapidly with little fatigue and few adverse events.[89] After the initial training session in person, a subsequent training session will take place at the patient's home via iPad/iPhone videoconference using a device provided to the patient to provide additional support and the opportunity to answer questions. Subsequent sessions will be done independently by patients, but study staff will be available via iPad/iPhone if questions arise.

4.1.2 Musclesound

Assessment of intramuscular adipose tissue (IMAT) and Intramuscular Glycogen Content (IMGC). IMGC and IMAT can be determined by ultrasound in different musclegroup throughout the body ,using MuscleSound® ultrasound technology. MuscleSound® ultrasound technology assessments of IMGC and IMAT have been validated against muscle-biopsies and CT/MRI imaging. Longitudinal and transverse ultrasound images of the m. rectus femoris, vastus lateralis, m. intercostalis / m. pectoralis, (head) m. temporalis, and/or (head) styloglossus. Complex grayscale analysis of the images was used to calculate the IMAT and IMGC; In addition, muscle thickness/Area and subcutaneous fat layer thickness (with greyscale analyses) are also assessed by ultrasound.

4.1.3 Body Composition Measures

Participants will have body composition assessed at the Duke Center for Living. To determine lean and fat mass changes participants will be assessed using air-displacement plethysmography (ADP). Briefly the BodPod machine is an ADP machine which uses whole-body densitometry to determine body composition (fat vs. lean). The machine uses very precise scales and volume displacement by sitting still inside the machine. The machine produces small volume changes inside the chamber and measures pressure responses to these changes. In total, each participant is inside the machine for no more than 5 minutes and accuracy is within 2% of that of dual-energy x-ray absorptiometry (DXA). Additionally, ADP has significantly fewer limitations than DXA, such as the weight of the participant, radiation exposure, cost and ease of use. In addition to ADP measurements, we will also measure waist and hip circumferences.

An alternative body composition measure if BodPod is unavailable is bioelectrical impedance spectroscopy (BIS) via a bioimpedance analysis (BIA) device. BIA equipment does not measure muscle mass directly, but instead derives an estimate of muscle mass based on whole-body electrical conductivity. Skeletal muscle has a large amount of water, and segmental BIS can separately assess intracellular water (ICW) and extracellular water (ECW), which are divided by the muscle cell membrane. Segmental BIS can distinguish ICW and ECW from the total water in a particular segment. Segmental BIS is advantageous for assessments of a localized (left/right) region (arm, leg and trunk) instead of only the whole-body ICW reflects muscle cell mass, whereas ECW represents the sum of interstitial fluid and blood plasma in extracellular space. The calculations of ICW and ECW are only affected by segmental volume, so the ECW/ICW ratio could indicate the ratio of non-contractile tissue to contractile tissue regardless of assessed somatotype (age, gender, disease state). InBody S10 will be the BIS device used to obtain BIA. InBody is a FDA approved device, and DUH-approved used in Standard of Care.

4.2 Cognitive function

4.2.1 The Repeatable Battery for the Assessment of Neuropsychological Status (RBANS)

RBANS is used as appropriate evaluation tools for neurocognition [90]. RBANS consists of multiple subtests and assesses attention, language, visuospatial/constructional ability, and immediate and delayed memory. RBANS is a relatively brief, and yet comprehensive, one-on-one administration tool [90]. RBANS is considered an established and validated tool by various studies that have illustrated its sensitivity in the detection of neurocognitive impairments [90-94]. RBANS evaluation produces five index scores as well as a total summary score, and total scaled score. Scores range from 40 to 160, with higher scores indicating better performance [95]. The scaled score classifies neuropsychological performance as: extremely low (69 and below), borderline (70–79), low average (80–89), average (90–109), high average (110–119), superior (120–129), and very superior (130 and above) [90,92].

4.2.2 The Trail Making Test

Trail Making Test parts A and B (TMT-A and -B) will be used for identification of cognitively impairment. TMT is an easily administered measure of visuomotor attention and executive function and is widely used in neuropsychological evaluations [96] In TMT-A assessment participants are asked to draw lines to connect consecutively numbered circles. In part B of the evaluation, participants are asked to connect consecutively numbered and lettered circles by alternating between the two sequences (1-A-2-B-3-C, etc.). Total time to completion will be used as outcome variable for both parts (ranging from 0 to 300 seconds, with higher scores indicating worse performance [95]. An adjusted mean T-score also will be calculated ranging from 0 to 100 (mean score 50, higher score indicating better performance). The T-score can be adjusted for age, sex, education and ethnicity. TMT-A and -B assessments will supplement the RBANS assessment for measures of neurocognition, assessing multiple cognitive domains (e.g., executive function, memory, attention, etc.) This combination will make our assessment more sensitive to detect milder cognitive deficits, and provides an objective measure of neurocognition, assessing multiple cognitive domains (e.g., executive function, memory, attention, etc.) [95,97].

4.2.3 Brief Cope

The items included in the Brief Cope represent an abbreviated version of the COPE Inventory. It has been used in research with breast cancer patients, with a community sample recovering from Hurricane Andrew, and with other samples as well. Assessment categories included in the Brief Cope include: Self-distraction, Active coping, Denial, Substance use, Use of emotional support, Use of instrumental support, Behavioral disengagement, Venting, Positive reframing, Planning, Humor, Acceptance, items Religion and Self-blame.

4.2.4 Delirium Observation Screening Scale (DOS)

The Delirium Observation Screening Scale is a 13-item observational scale of verbal and nonverbal behavior. The observations can be done during regular care. The DOS is used to optimize recognition of delirium.

A study team member will observe the participant during the 6T and score the participant.

4.3 Physical and Cognitive function, Mental health, and social support

4.3.1 Patient Reported Outcomes Measurement Information System (PROMIS) Instruments

The Patient-Reported Outcomes Measurement Information System (PROMIS) developed self report, efficient physical, mental, and social health measures for individuals facing a variety of health conditions and complications.[98] The physical health domain includes physical function; mental health consists of cognitive function and measures of depression and anxiety; and social health encompasses relationships and participation in common social roles and activities. Each of the PRO instruments will be scored on the T-score metric, where 50 is the mean of a reference population and 10 is the standard deviation of that population.

PROMIS Physical Function – The Patient-Reported Outcomes Measurement Information System (PROMIS) developed self-report, efficient physical, mental, and social health measures for individuals facing a variety of health conditions and complications. The physical health domain includes physical function; mental health consists of cognitive function and measures of depression and anxiety; and social health encompasses relationships and participation in common social roles and activities. Each of the PRO instruments will be scored on the T-score metric, where 50 is the mean of a reference population and 10 is the standard deviation of that population.

PROMIS-Anxiety – The Patient-Reported Outcomes Measurement Information System (PROMIS) developed self-report, efficient physical, mental, and social health measures for individuals facing a variety of health conditions and complications. The physical health domain includes physical function; mental health consists of cognitive function and measures of depression and anxiety; and social health encompasses relationships and participation in common social roles and activities. Each of the PRO instruments will be scored on the T-score metric, where 50 is the mean of a reference population and 10 is the standard deviation of that population.

PROMIS-Emotional Support – The Patient-Reported Outcomes Measurement Information System (PROMIS) developed self-report, efficient physical, mental, and social health measures for individuals facing a variety of health conditions and complications. The physical health domain includes physical function; mental health consists of cognitive function and measures of depression and anxiety; and social health encompasses relationships and participation in common social roles and activities. Each of the PRO instruments will be scored on the T-score metric, where 50 is the mean of a reference population and 10 is the standard deviation of that population.

PROMIS-Social Isolation – The Patient-Reported Outcomes Measurement Information System (PROMIS) developed self-report, efficient physical, mental, and social health measures for individuals facing a variety of health conditions and complications. The physical health domain includes physical function; mental health consists of cognitive function and measures of depression and anxiety; and social health encompasses relationships and participation in common social roles and activities. Each of

the PRO instruments will be scored on the T-score metric, where 50 is the mean of a reference population and 10 is the standard deviation of that population.

4.3.2 Patient Health Questionnaire-9 (PHQ-9)

The PHQ depression screening tool commonly used in clinical settings. While it cannot definitely diagnose depression[112] it is comparable to longer screeners.[113-115] The tool consists of questions about mood and anhedonia. The PHQ-9 consists of 9 items, each of which is scored 0 to 3, giving a 0 to 27 severity score. Thus, the instrument assesses the severity and presence of depression diagnostic criteria, making it appealing over longer instruments. A positive screen would lead to a referral to psychology or psychiatry services.

PHQ9 will be performed on all allogeneic transplant patient candidates at the indicated timepoints as part of our clinical standard of care, results will be abstracted from the patient chart and entered into our RedCap database (see Data Safety Monitoring Plan)

4.3.2 Lorig Self Efficacy

A validated measure of self-efficacy and the ability to "do for oneself."

4.3.3 Ego Resiliency

A validated measure of the capacity of individuals to adapt to constantly changing situations and demands.

4.4 Nutrition and diet

4.4.1 Patient Generated Subjective Global Assessment

The scored Patient-Generated Subjective Global Assessment (PG-SGA) is a quick and accurate nutrition assessment tool that has been used in the oncology[105] and HCT[35] settings. As a revision of the Subjective Global Assessment,[106] it evaluates an individual's physical status as it relates to nutrition and nutritional impact symptoms. The patient completes sections 1 through 4 (i.e. Weight, Food Intake, Symptoms, and Activities and Function), while the health professional evaluates Worksheet 2 - Disease and its relation to nutritional requirements (Section 5), Worksheet 3 - Metabolic Demand (Section 6), and performs Worksheet 4 - Physical Exam (Section 7). Additionally, the health professional completes Worksheet 1 to score weight loss (if applicable) and Worksheet 5 to provide a subjective global rating of the respondent's overall nutritional status (e.g. well-nourished, moderately malnourished, or severely malnourished). Scores for each section can range from 0-4 depending on severity and nutritional impact. The sum of all scores provides the Total PG-SGA score, which can be used to indicate the need for intervention.[105] For example, a score of ≥ 9 indicates a need for critical intervention.

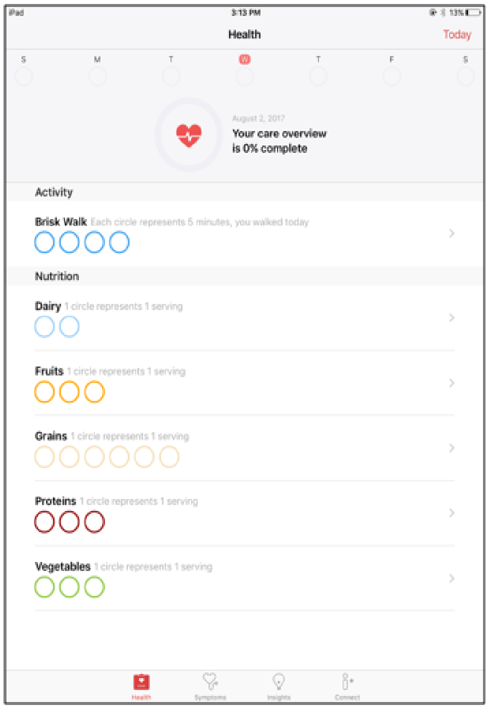

4.4.2 24- Hour food recalls RPOP participants will be called at the 6T by a study team member to review their dietary intake for the previous 24 hours. The participants will just be responsible for reporting food intake to the study team member and they will record in their study records.

4.4.3 Food groups tracking

R-POP participants will be asked to track their daily dairy, fruit, grain, protein, and vegetable intake using one of two mobile health apps developed specifically for HCT patients.

The first app is called Technology Recordings to better Understand BMT (TRU-BMT). This is just one section of the application that patients will complete. This Duke-established

app has already been piloted in Duke HCT patients (Pro00068979, PI Shah, co-I Sung) and was rated favorably as both easy to use and helpful (abstract presented at the American Society of Hematology 2017 Annual Meeting). To the right is a screenshot from the app, which shows what a user will see and complete over the course of a day. Each time a respondent ticks a circle under the food groups, indicating they consumed one serving of the minimum recommended food groups, the outline of "care overview" circle located towards the top center of the display begins to get highlighted/filled indicating the progression of attaining daily goals graphically and as a percentage of 100.

The second app is called SplendoFit and it is a Multi-modal real-time cloud-based AI platform for fast and non-invasive (sequential) point-of-care assessments profiling patient's (systemic) cardio-pulmonary metabolic phenotype. It connects to a range of high-resolution sensors for continuous monitoring of a wide variety of high-resolution digital biomarkers. Output is directly visualized for easy interpretation of results with direct communication with healthcare system (Epic) for billing and reporting (RedCap). The app will give insight of the general health- and nutritional status with the use of different validated questionnaires. To the left is a screenshot from the app, which shows what a user will see and complete over the course of a day.

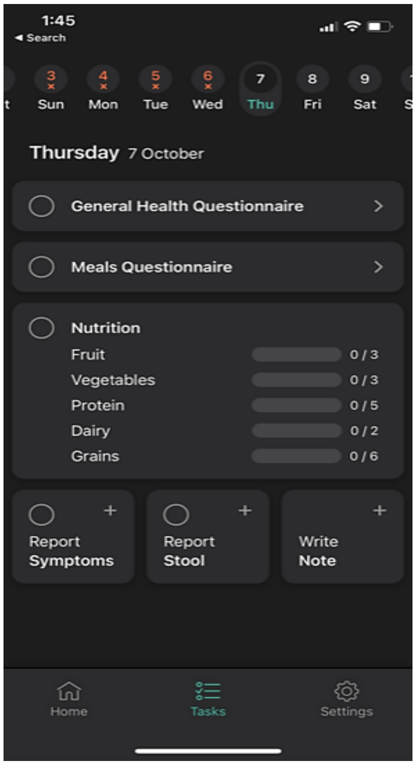

4.4.4 Vit D, Vit A, albumin, prealbumin, A1c; If PONS+ also thiamine, Zn, Cu, selenium**

Performed on all allogeneic transplant patient candidates at the indicated time points as part of our clinical standard of care, results will be abstracted from the patient chart and entered into our RedCap database (see Data Safety Monitoring Plan)

4.4.5 Food Security

RPOP participants will be given the USDA food security survey to review their food securities or insecurities. This is a 10 item survey.

4.4.6 DHQ3

RPOP participants will be asked to complete the Diet History Questionnaire III to provide their nutritional intake averages for the past month.

4.5 Applications

Developed specifically for HCT patients called Technology Recordings to better Understand BMT (TRU-BMT). This Duke-established app has already been piloted in Duke HCT patients (Pro00068979, PI Shah, co-I Sung) and was rated favorably as both easy to use and helpful (abstract presented at the American Society of Hematology 2017 Annual Meeting). Below are screenshots from the app, which shows what a user will see and complete over the course of a day.

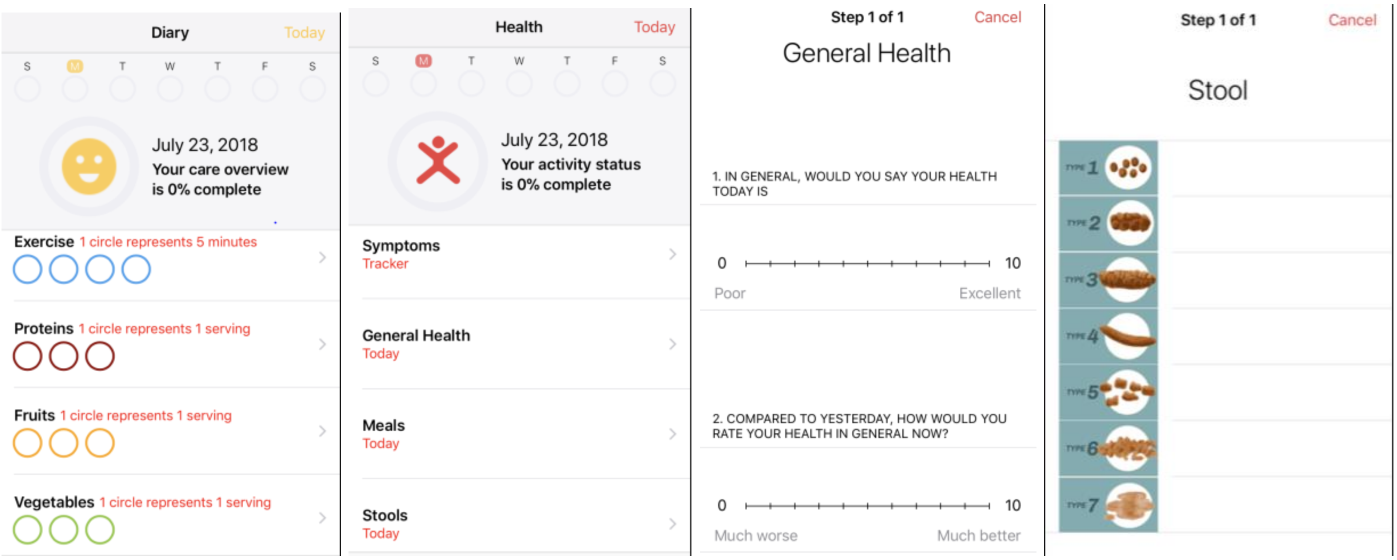

SplendoFit is a multi-modal real-time cloud-based AI platform for fast and non-invasive (sequential) point-of-care assessments profiling patient’s (systemic) cardio-pulmonary metabolic phenotype. Connects to a range of high-resolution sensors for continuous monitoring of a wide variety of high-resolution digital biomarkers. Output is directly visualized for easy interpretation of results with direct communication with healthcare system (Epic) for billing and reporting (RedCap) . The app will give insight of the general health- and nutritional status with the use of different validated questionnaires. Below are screenshots from the app, which shows what a user will see and complete over the course of a day.

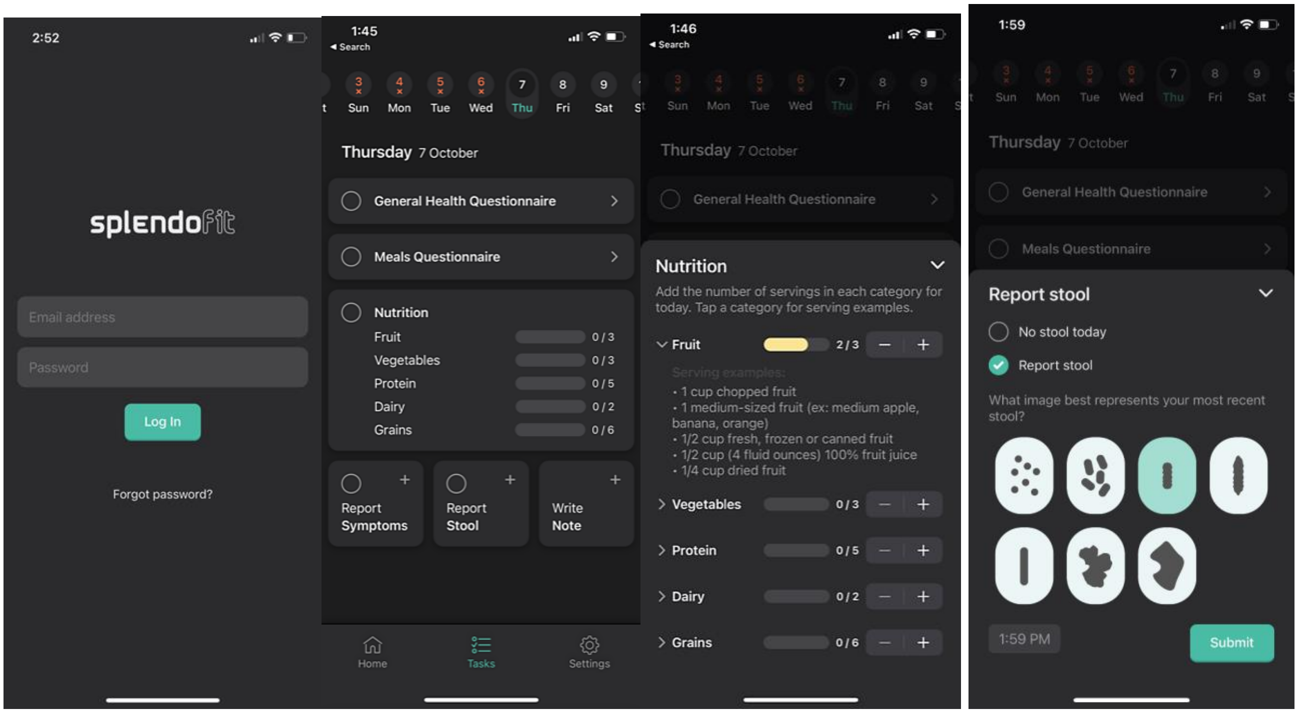

Subjects are asked to make daily entries and data will be pulled at the end of the study. Subjects who do not complete daily entries will not be recorded as a deviation.

4.6 Caregiver support

In addition to the measures described in the table below, caregivers will also complete the PROMIS Depression, Anxiety, Emotional Support, and Social Isolation instruments and the Patient Health Questionnaire (PHQ)-2 and -9. The PHQ-2 is a short depression screening tool commonly used in clinical settings. While it cannot definitely diagnose depression[112] it is comparable to longer screeners.[113-115] The tool consists of 2 questions about mood and anhedonia. The PHQ-2 has been found to have a 93% negative predictive value and 38% positive predictive value, with a 97% sensitivity and 67% specificity.[115] The PHQ-9 consists of 9 items, each of which is scored 0 to 3, giving a 0 to 27 severity score. Thus, the instrument assesses the severity and presence of depression diagnostic criteria, making it appealing over longer instruments. If the either of the PHQ-2 questions, which are the first 2 questions on the PHQ-9, are affirmed the patient participants will be asked to complete the remaining 7 questions of the PHQ-9. A positive screen would lead to a referral to psychology or psychiatry services.

Additional assessments for caregivers are outlined in the table below.

Table 2 Measures for Caregivers

Domain	Assessment	Time (min)	Timepoints	Intervention
**Caregiver Support (to be completed by caregiver)**	Caregiver Strain<br>Preparedness for Caregiving<br>FACT-GP<br>PHQ2/PHQ9<br>PCL-5<br>Ego Resiliency<br>PROMIS Depression<br>PROMIS Anxiety<br>PROMIS-Emotional Support<br>PROMIS-Social Isolation	3<br>2<br>5<br>1<br>4<br>3<br>2<br>2<br>2<br>2	6T<br>6T<br>6T<br>6T<br>6T<br>6T<br>6T<br>6T<br>6T<br>6T	if PHQ9 or PCL5 pos, referral to psychiatry **
**Microbiota**	Skin Swab<br>Stool Collection	5<br>5	6T<br>6T + D0, 7, 14, 21, Y2	NA
	Total time (caregivers):	36		

PROMIS=Patient Reported Outcomes Measurement Information System; 6T=6 main timepoints (pre-transplant: new patient evaluation [NPE] and sign off [SO] prior to transplant; peri-transplant: day 30 [D30], discharge home [DC] (approximately D90); post-transplant: day 180 [D180], year 1 [Y1])
** Per investigator/MD discretion

<u>Collection Windows:</u>
New Patient Evaluation: +30 days of New Patient Evaluation Clinical appointment
Sign Off: -/+ 7 days of Sign Off Clinical appointment
Day 0, Day 7, Day 14, Day 21, Day 30, Day 60: -/+ 7 days
Discharge: -/+ 14 days
Day 180, Year 1: -/+ 30 days

To discuss caregiver assessments in more detail:

Caregiver strain – a validated measure of caregiver stress and strain

Preparedness for Caregiving – a measure of caregiver comfort and sense of preparedness for the task of caregiving

FACT-GP – a validated general measure of functional wellbeing

PHQ2/PHQ9 – see above under patient assessments

PCL-5 – see above under patient assessments

Ego Resiliency – see above under patient assessments

PROMIS Depression – see above under patient assessments

PROMIS Anxiety – see above under patient assessments

PROMIS-Emotional Support – see above under patient assessments

PROMIS-Social Isolation – see above under patient assessments

Skin Swab – see above under patient assessments

Stool Collection – see above under patient assessments

4.7 Global Assessments and Symptoms

4.7.1 EQ-5D-5L

The EuroQol Group created the EQ-5D-5L to measure health-related quality of life and it has been validated in those with cancer.[117] The 5D represents 5 dimensions: mobility, self-care, usual activities, pain/discomfort, and anxiety/depression. Five levels of severity are measured, as indicated by the "5L," ranging from "no problems" to "extreme problems."[118] The dimensions and levels are the descriptive system of the questionnaire. The next section is a measure of self-rated health using a visual analog scale. All scores are converted into a summary index and the instrument takes approximately 2-4 minutes to complete.

EQ-5D-5L will be performed on all allogeneic transplant patient candidates at the indicated timepoints as part of our clinical standard of care, results will be abstracted from the patient chart and entered into our RedCap database (see Data Safety Monitoring Plan).

4.7.2 Hematopoietic Cell Transplantation-Comorbidity Index (HCT-CI)

From the HCT-CI website, http://www.hctci.org/:

"The HCT-CI was initially designed using clinical data from 1055 consecutive patients treated with allogeneic HCT from 1997 to 2004 at the Seattle Cancer Care Alliance (SCCA)/Fred Hutchinson Cancer Research Center (FHCRC).[119] The index was validated among patients transplanted at the SCCA/FHCRC, [120] as well as other transplant institutions world-wide. [121{Barba, 2010 #1383,122] } Multi-institutional validation of the predictive power of the HCT-CI is ongoing. The HCT-CI was shown to be an important decision-making instrument for choosing appropriate conditioning regimens for patients with acute myeloid leukemia or myelodysplastic syndromes[123] and those with lymphoma or chronic

lymphocytic leukemia.[124] The HCT-CI together with the degree of severity of blood cancer could be used to stratify outcomes of patients 60 years or older who were treated with a minimally-toxic nonmyeloablative conditioning regimen and allogeneic HCT.[125] Recently, a combined HCT-CI/age index was designed and validated that takes into account the burden of comorbidities as well as increasing age in risk-assessment. [126]"

HCT-CI is evaluated on all allogeneic transplant patient candidates as part of our clinical standard of care. HCT-CI will be reviewed as part of this study.

4.7.3 National Comprehensive Cancer Network Distress Thermometer

Distress in the form of depression or anxiety can impact adherence to cancer medical treatments and a patient's affect. The National Comprehensive Cancer Network Distress Thermometer measures distress on a scale of 0-10, with a score of 5 of more indicating the need for referral and deeper evaluation.[127] Using such a rating system has proven to be beneficial in the oncology field[128] and the NCCN hopes using such a scale would help patients to discuss the emotions they are experiencing around their diagnosis, symptoms, and treatment of cancer.[129] NCCN Distress Thermometer will be performed on all allogeneic transplant patient candidates at the indicated timepoints as part of our clinical standard of care, results will be abstracted from the patient chart and entered into our RedCap database (see Data Safety Monitoring Plan)

4.7.4 Two Factor Consideration of Future Consequences Scale:

A validated measure of individual differences in considerations of future consequences. Subscales assess concern with future consequences (CFC-Future) and concern with immediate consequences (CFC-Immediate)

4.7.5 Brief Resilience Scale (BRS):

The brief resilience scale (BRS) was created to assess the ability to bounce back or recover from stress.

4.7.6 Stool Collection and Microbiome Analysis:

20gm (plum size) stool samples will be collected from patients, designated for this study following standard clinical protocols (e.g. for Clostridium difficile collection) and may be stored at 4°C for up to 24 h before freezing at -80°C for batch sequencing and microbiome analysis. Subjects will also be offered to consent to the Duke Microbiota Repository (IRB Pro00078566) for storage of excess or unused specimens. If no consent to store the samples is obtained, the samples will be destroyed.

To analyze the gut microbiome, DNA will be extracted from fecal samples[130] and gut flora bacterial density will be quantified using real-time quantitative polymerase chain reaction (qPCR) as described.[131] We will amplify 16S ribosomal RNA (rRNA) using Illumina HiSeq platform and analyze the data using the Qiime script package with parallel processing.[132] Sequences will be de-noised and clustered at 97% identity using USEARCH and aligned to the 16S rRNA gene, using the align.seqs.py wrapper with the PyNAST algorithm and Greengenes reference alignment. Based on these results, we will calculate diversity (Shannon Diversity, primary endpoint, and Chao1) and construct phylogenetic trees using computational analysis software. Pairwise comparisons will be made between pre- and post-HCT samples and in-home vs. standard care groups. We will also make longitudinal evaluations within individual patients.

4.7.7 Skin swab and Microbiotia Assessment:

Skin samples will be obtained prior to conditioning and at day 30, 60 and 100 post HCT among 15 patients undergoing standard and 15 patients undergoing home transplant protocol.

Skin microbiotia assessment will include a sample of a 2×2-cm area of the target region collected by swabbing the skin to pick up bacteria located on the skin. Specimens may be obtained from the following sites: (1) Forearm, antecubital fossa, (2) Retroauricular region, (3) Buccal, (4) Back, (5) Abdomen, (6) If applicable, areas with active GVHD or rash. Samples will be snap frozen and stored for batch sequencing and microbiome analysis. Subjects will also be offered to consent to the Duke Data and Specimen Repository (IRB Pro00006268) for storage of excess or unused specimens; if no consent to store the samples is obtained, the excess samples will be destroyed.

Additionally, because co-habitation may affect the microbiota of the involved individuals, we will also collect stool and skin samples from caregivers to analyze in conjunction from stool and skin samples from patients as described above. Samples will be collected as per Table 2. Samples not used during this research will be stored in the Duke Data and Specimen Repository (IRB Pro00078566) unless the caregiver does not consent to the Repository. If no consent to store the samples is obtained, the excess samples will be destroyed.

4.7.8 Blood Collection - Biomarkers, Metabolomics, and Flow Cytometry:

Research blood samples will be collected at baseline, day 21, month 3 and 1 year post-transplant and will include no more than 16ml of blood per collection event (two NaH green top + one EDTA purple top) or 128ml (about 2 fluid ounces) in an 8-week period.

Subjects may be consented to multiple studies that contain the same research blood collection and analysis (specifically Pro00051024, Pro00089697, Pro00092963). If this situation occurs, duplicate research blood samples will not be collected. Instead, data derived from either Pro00051024 or Pro00089697 may be transferred to this study for analysis and reporting purposes.

Research blood samples will be processed as follows prior to biomarker, metabolomics, and flow cytometry analysis:

For Biomarker and Metabolomics analysis:

1. Draw one 4ml lavender top (K_2EDTA) tube.
2. Invert tube 10 times to mix blood.
3. Centrifuge at 4°C at 2500 x g for 15 minutes.
4. Remove plasma from tube and transfer into sterile 15ml conical tube.
5. Repeat centrifuge at 4°C at 2500 x g for 15 minutes.
6. Aliquot ≥1.0ml of plasma equally into 2.0ml cryovials, one for metabolomics, one for plasma biomarkers
7. Freeze at -80°C.

For Flow Cytometry analysis:

1. Draw two green top (Sodium Heparin) tubes.
2. Dilute sample with phosphate-buffered saline (PBS) at a ratio of 1:1.
3. Ficoll sample in accordance with laboratory SOP.
4. For every 1mL of PBMCs (peripheral blood mononuclear cell) collected add 100 µL DMSO (dimethyl sulfoxide).
5. Immediately freeze sample at -80°C.

Metabolomics

Approximately 1ml of plasma from the EDTA tube will be used for metabolomics analysis.

Targeted metabolomics: acylcarnitines and amino acids are analyzed using stable-isotope-dilution techniques. Measurements are made by flow-injection, tandem mass spectrometry, using serum or plasma sample preparation methods described previously (57, 58). Data are acquired using a triple-quadrupole detector equipped with an Acquity ultra-high-pressure liquid-chromatography system and controlled by the MassLynx 4.1 software platform, all from the Waters Corporation (Milford, MA).

Exploratory, non-targeted metabolomics: via gas chromatography/mass spectrometry (GC/MS), metabolites are extracted from blood (serum or plasma) with methanol, methoximated in dry pyridine, and then silylated with *N*-methyl-*N*-(trimethylsilyl) trifluoroacetamide. Samples are analyzed on a 7890B GC connected to a 5977B MS (Agilent Technologies, Santa Clara, CA), equipped with two wall-coated, open-tubular (WCOT) GC columns connected in series (Agilent part 122-5512, DB5MS, 15 meters in length, 0.25 mm in diameter, with an 0.25-µm luminal film), separated by a microfluidic flow splitter to enable hot back-flushing at the end of each run (59). Data are acquired by scanning from *m/z* 600 to 50 as the oven ramped from 70 to 325 °C. Data are then deconvoluted using AMDIS software (60). Metabolites are identified using our retention-time-referenced spectral library (24, 61, 62), which is based in part on that of Kind *et al.* (2009) (63). Reported data are log-base-two transforms of the areas of deconvoluted peaks.

Blood-based biomarker analysis

Approximately 1ml of plasma from the EDTA tube will be used for metabolomics analysis

Assay methodology: Our collaborators art Duke, Dr. Andrew Nixon and Dr. Virginia Krauss, have developed and optimized an innovative multiplex assays evaluating angiogenic, stromal, and inflammatory markers and markers of aging (the Pepper Panel). For these assays, all collected samples are platelet-poor EDTA plasma. Briefly, plasma samples to be used in these analyses will be identified and cross-checked for provision of informed consent and the availability of relevant clinical data. Approved samples will be aliquoted and stored in -80°C freezer, ready for analysis. Plans for optimal sample allocations as well as customized ELISA plate arrangements will be developed. Any study sample that falls outside the linear portion of the standard curve will be retested. Samples that read below the limit of detection will be retested, if possible. Samples that read above the linear portion of the standard curve will be serially diluted and retested to obtain accurate measurements. Any analytes that do not meet the aforementioned criteria will result in the sample being re-evaluated. All research personnel are blinded to clinical outcomes.

In order to develop the optimal arrays, we have the opportunity to leverage 3 multiplex protein array systems from Aushon Biosystems (Billerica, MA), ProteinSimple (San Jose, CA) and Meso Scale Diagnostic (MSD), LLC. (Rockville, MD)**.**

Regulatory Considerations: Blood-based biomarkers have many advantages compared to tissue-based markers, including lower risk to the patient, lower cost, and easy collection along the continuum of care, including at the start of treatment, in the setting of response, and at the time of progression. Therefore, any blood-based biomarker approach would provide an easier and more cost-effective method for the detection of cancer or the guidance of cancer treatment decisions. The development of blood-based biomarkers utilizing standard or multiplex ELISA techniques is readily adapted to clinical diagnostics. Importantly, many of the markers are drug targets, either in development or already approved. These features make any findings from our blood-based approach highly actionable, both diagnostically and therapeutically.

Statistical considerations for the blood based biomarker analysis: The characteristics of blood analytes will be investigated using a variety of measures. Baseline and on-treatment samples will be assessed. Coefficients of variation will be used to assess the dispersion of each marker. Pair-wise correlations among the analytes will be estimated using Kendall's Tau. For additional visualization of these analytes, plots of dendrograms and/or heat maps with clustering relationships among the analytes will be presented. Descriptive statistics will also be presented.

Overview of some key QA/QC considerations: The validation protocol described in more detail below will assess and provide data for all markers tested, including but not limited to: standard curves for each marker, sensitivity, dynamic range, precision (intra-assay and inter-assay), and cancer patient marker ranges, sample type (serum, EDTA plasma, citrated plasma), assay specificity, and sample stability evaluating key timing and temperature considerations.

Standard Curves and Dynamic range: The standard curves for each marker will be determined according to each platform manufacturer. For the plate-based systems (Aushon Biosystems (Billerica, MA) and Meso Scale Discovery (MSD) platform (Rockville, MD)), we will provide representative 8-point standard curves for each marker assessed, demonstrating the dynamic range of the assay, expected to exhibit 3-4 orders of magnitude. Protein standards are added in duplicate for each multiplex array tested. The standard curve values will be generated by averaging 2 replicates of each calibrator in each plate. We will optimize the dilution used for each array in order to maximize the linear part of the

standard curve. The microfluidic ELLA system has built-in, preset standard curves that are imported into the system during analysis. Use of these factory-calibrated standards facilitates the work flow and directly correlates with daily in-lab calibration experiments.

Sensitivity: For the markers of interest, we will establish the Limits of Detection (LOD), Lower Limits of Quantitation (LLOQ) and Upper Limits of Quantitation (ULOQ). LOD is the mean of the zero calibration standards + 2 standard deviations, LLOQ is the lowest calibration standard with back-calculated concentrations having CVs <20% and relative error <25%, and ULOQ is the highest calibration standard with back-calculated concentrations having CVs <20% and relative error <20%.

The Coefficients of Variation (CV): The average %CV will be determined for all markers. The data will be representative of intra-assay variation. In our previous analyses, we have demonstrated that most makers have %CV in the range from 2 -10%.

Assay Specificity: Specific multiplex arrangements will be evaluated for specificity by evaluating: 1) all detection Abs in the well and 2) only the specific detection Ab of interest.

Flow Cytometry

Whole blood samples, will be analyzed and monitored for outcomes such as immune cell differentiation, immune response and development of GVHD**.** Flow cytometry will be performed -b(7). Briefly, whole blood will be used for isolation of about 1×10^6 mononuclear cells. The mononuclear cells will be incubated with titrated antibodies for 15 minutes at room temperature in the dark. mononuclear cells will be lysed with FACS lysing solution (BD Biosciences, San Jose, CA). Cells will be stained with appropriate antibody cocktail and then analyzed using a FASCanto flow cytometer equipped with FACSDiva software (BD Biosciences). Absolute cells counts will be determined using a Flow-Count Fluorospheres (Beckman Coulter, Brea, CA). Examples of antibodies include: FITC-conjugated anti-CD62L (clone MEL-14), R-PE–conjugated anti-CD45.1 (clone A20), cy-chrome-conjugated anti-CD44 (clone IM7), and their isotype controls (all from BD PharMingen); and FITC-conjugated anti–H-2Db (clone CTDb), PE-conjugated anti-CD4 (clone CT-CD4), B220 (clone RA3-6B2), PE-Texas red–conjugated anti-CD4 (clone RM4-5), tricolor-conjugated anti-CD4 (clone CT-CD4), CD8α (clone CT-CD8α), and their isotype controls (Invitrogen, Carlsbad, CA).

4.8 Safety endpoints

Treatment-related mortality (TRM) and grade 3-4 adverse events as defined by the National Cancer Institute Common Terminology Criteria for Adverse Events (NCI-CTCAE) [135] will be monitored that are related to study interventions (e.g. exercising).

5.0 ANTICIPATED TOXICITIES

We do not anticipate any major toxicities occurring with participation in our investigation. The study interventions are evidenced-based and aimed at improving any health concerns the participants may have. To ensure the utmost safety of our participants going through exercise training **(as described above in section 4.1)** study staff will ensure that all patients who take beta-blockers will have taken their medication prior to training to avoid any blood pressure-related events.

6.0 STATISTICAL CONSIDERATIONS

General Considerations: All analyses will be summarized using appropriate effect estimates, confidence intervals, and p-values. All secondary and additional analyses will be evaluated at the standard 0.05 significance level. Descriptive statistics, including graphical displays, will be used to summarize all study variables. For continuous variables, means, standard deviations, percentiles, ranges, box plots and histograms will be generated. For categorical variables, frequencies and proportions will be generated. For time to event variables, event rates and survival curves will be generated. Statistical analyses will be performed using SAS for Windows (Version 9.3 or above: SAS Institute, Cary, NC) and the R statistical computing platform (R Core Team 2013; http://www.R-project.org/).

Categorical outcomes will be analyzed using chi-square techniques and logistic regression modeling. Length of stay outcomes will be analyzed using generalized linear models with appropriate link functions. Continuous, longitudinal outcomes such as the FACT-BMT and EQ-5D-5L will be analyzed using linear mixed regression modeling. Other continuous results (such as PG-SGA and numbers of days exercised and amount of exercise per day) will be analyzed using standard t-test approaches and correlated with outcomes. We will analyze TRM and CTCAE using Kaplan-Meier methods, log-rank tests, and Cox proportional hazard regression modeling.

6.1 Analysis of the Microbiota:

Shannon diversity index will be compared using unpaired two-sided Student's *t* tests with a more stringent cut-off of 0.0125 given multiple comparisons, by the Bonferroni correction for 4 time periods of independent comparisons. Comparisons of bacterial populations will be made by using the Adonis non-parametric method from within the Qiime package using UnifFrac distance matricies from the populations being compared. P values will be calculated with $\alpha = 0.05$. Metatstats will be used for further statistical analyses of population structure, membership, and diversity with metadata such as time from transplant and dietary measurements such as caloric intake [145]. Changes in specific bacterial families of interest will be compared using a two-sided Student's *t* test, with normality confirmed by D'Agostino and Pearson omnibus test with $p \leq 0.05$. All other comparisons will be done using two-sided Mann-Whitney tests. The R packages vegan [146], phyloseq [147], APE [148], randomForest [149], and arules [150] will be used for identification of associative patterns between taxa most associated with home care and hospital care recovery and other care and disease-related metadata. Comparisons will also be made between pairs of patients and caregivers to determine these effects.

6.2 Power and sample size calculations:

The primary hypothesis of interest for this safety and feasibility study is that at least 50% of enrolled patients will complete more than 50% of their high intensity interval training sessions. With a sample size of 35 patients, our study will be able to estimate a completion rate of 50% to within a 95% confidence interval of +/- 19%. For higher completion rates, such as 75%, we will be able to estimate to within a 95% confidence interval of +/- 17%[151].

As noted in the exercise intervention section, in order to participate in the exercise intervention, subjects must first meet all inclusion and exclusion criteria and consent to participate, after which they will under cardiopulmonary exercise testing (CPET). CPET will be reviewed by a physician and subjects will not be able to participate in the intervention unless cleared by a physician. Therefore, not every patient who consents will participate in the intervention. Because our goal is test the intervention in 30 patients, and assuming a 10% drop out rate (subjects who consent to participate but do not pass CPET testing), we will consent 35 subjects with the intent that 30 will participate in the intervention.

7.0 DATA SAFETY MONITORING PLAN

Throughout the study, we will collect data on treatment-related mortality (TRM) and grade 1-4 adverse events as defined by the National Cancer Institute Common Terminology Criteria for Adverse Events (NCI-CTCAE) [135] will be monitored during weekly or monthly assessments, that are related to study interventions (e.g. Exercising)..

For all the above assessments and interventions, data will be obtained using electronic surveys through RedCap or in person assessments (e.g. CPET testing) and entered into RedCap. While the medical record number will be kept by study staff to enable access to the medical record for event monitoring and data abstraction, data will be entered and stored using a coded study number; likewise biospecimens will be stored under this coded study number as opposed to the patient medical record number. Other identifiable information include treatment dates (e.g. date of new patient evaluation, date of study evaluations, transplant date, etc.).

Use of previously collected biospecimens, data, or records

As part of this study, we will obtain data from the patient's medical record (see above section). Study staff will abstract this information using the patient's medical record number and enter the data into RedCap as above.

Potential risks to subjects with study interventions, procedures, or interactions

This is a low risk intervention study that does not involve the use of a study drug or investigational device. We do not anticipate any major toxicities occurring with participation in our investigation. The study interventions are evidenced-based and aimed at improving any health concerns the participants may have. As will all studies, there is a potential risk of loss of privacy and confidentiality associated with all study procedures. These are addressed in the below section on protection against risks.

Additional risks with patient assessments and interventions:

Short physical performance battery – abstracted from records, no additional risk

6 minute walk – abstracted from records, no additional risk

30 second sit/stand – abstracted from records, no additional risk

Grip strength – abstracted from records, no additional risk

Fried - abstracted from records, no additional risk

PROMIS Physical Function – questionnaire/survey, minimal risk

CPET – While there are risks associated with any exercise activity, such as chest pain, shortness of breath, muscle cramping or discomfort, musculoskeletal injury, exacerbation of underlying coronary disease, patient with a known history of at-risk coronary conditions will be excluded, and this activity will be performed in a monitored setting by trained professionals. To ensure the utmost safety of our participants going through exercise training, study staff will ensure that all patients who take beta-blockers will have taken their medication prior to training to avoid any blood pressure-related events. This assessment would be considered low risk

Montreal Cognitive Assessment – abstracted from records, no additional risk

PROMIS Cognitive Function – questionnaire/survey, minimal risk

RBANS – questionnaire/survey, minimal risk

Trials Test - questionnaire/survey, minimal risk

Brief Cope – questionnaire/survey, minimal risk

PHQ2/PHQ9 – abstracted from records, no additional risk

PC-PTSD/PCL-5 – abstracted from records, no additional risk

PROMIS Depression – questionnaire/survey, minimal risk

PROMIS-Anxiety – questionnaire/survey, minimal risk

Peri-operative nutrition screen – abstracted from records, no additional risk

PG-SGA - questionnaire/survey, minimal risk

Vit D, Vit A, albumin, prealbumin, A1c; if PONS+ also thiamine, Zn, Cu, selenium** – abstracted from records, no additional risk

24 Hr Food Recall – questionnaire, minimal risk

Food group tracking – questionnaire/survey/survey, minimal risk

PROMIS-Emotional Support – questionnaire/survey/survey, minimal risk

PROMIS-Social Isolation – questionnaire/survey/survey, minimal risk

Lorig Self Efficacy – questionnaire/survey/survey, minimal risk

Ego Resiliency – questionnaire/survey/survey, minimal risk

Two Factor Consideration of Future Consequences Scale (CFC-14) – questionnaire/survey/survey, minimal risk

Brief Resilience Scale (BRS) – questionnaire/survey/survey, minimal risk

NCCN Distress Thermometer – abstracted from records, no additional risk

HCT-CI (completed by MD) – abstracted from records, no additional risk

FACT-BMT – abstracted from records, no additional risk

EQ-5D-5L – abstracted from records, no additional risk

Delirium Observation Screening Scale (DOS) (completed by study team member) – observational, no additional risk

Financial Assessment – abstracted from records, no additional risk

Skin Swab – risk of mild discomfort and irritation from skin swabbing, self-limited and will stop on request, minimal risk

Stool Collection – risk of mess with transferring stool sample from hat into collection container, minimal risk

Blood collection – care will be taken to prevent excess blood draw, minimal risk

Interval Training – While there are risks associated with any exercise activity, such as chest pain, shortness of breath, muscle cramping or discomfort, musculoskeletal injury, exacerbation of underlying coronary disease,

patient with a known history of at-risk coronary conditions will be excluded, as with patients with abnormal findings on CPET testing. To ensure the utmost safety of our participants going through exercise training, study staff will ensure that all patients who take beta-blockers will have taken their medication prior to training to avoid any blood pressure-related events. Furthermore, the first training session will be performed in a monitored setting by trained professionals; the second session (first session at home) will be performed over a monitored video link by trained professionals, which will also occur weekly the first month, monthly for the remainder of the study, and as needed or requested by study staff or subjects; subjects always have the option to return in person for additional sessions. Therefore, these risks will be mitigated by supervision and training and this intervention is deemed low risk.

Referral to psychology/psychiatry, if warranted – while there may be risks of patient psychological distress associated with this referral, the likelihood of benefit from seeing a psychologist/psychiatrist to someone with potential depression or PTSD is outweighed by the risks. There may be financial risks associated with psychology/psychiatry referrals depending on insurance. Low risk.

Additional referral to social work, if warranted – while there may be risks of patient psychological distress associated with this referral, the likelihood of benefit from seeing a social worker to someone with social support needs is outweighed by the risks. Low risk.

Likewise, if psychological distress arises from any of the above questionnaires, assessments, or interventions, psychology, psychiatry, and social work will be available to assist. There may be financial risks associated with psychology/psychiatry referrals depending on insurance.

Additional risks with caregiver assessments and interventions:

Caregiver strain – questionnaire/survey/survey, minimal risk

Preparedness for Caregiving – questionnaire/survey/survey, minimal risk

FACT-GP – questionnaire/survey/survey, minimal risk

PHQ2/PHQ9 – while, by inquiring into questions related to depression, this may engender psychological distress, psychology and psychiatry services will be available to manage any distress (and in fact may be recommended based on responses)

PCL-5 – while, by inquiring into questions related to depression, this may engender psychological distress, psychology and psychiatry services will be available to manage any distress (and in fact may be recommended based on responses)

Ego Resiliency – questionnaire/survey/survey, minimal risk

PROMIS Depression – see above under patient assessments/interventions

PROMIS Anxiety – see above under patient assessments/interventions

PROMIS-Emotional Support – see above under patient assessments/interventions

PROMIS-Social Isolation – see above under patient assessments/interventions

Skin Swab – see above under patient assessments/interventions

Stool Collection – see above under patient assessments/interventions

Referral to psychology/psychiatry, if warranted – see above under patient assessments/interventions

Additional referral to social work, if warranted – see above under patient assessments/interventions

As with patients, if caregiver psychological distress arises from any of the above questionnaires or assessments, or interventions, psychology, psychiatry, and social work will be available to assist. There may be financial risks associated with psychology/psychiatry referrals depending on insurance.

Alternative treatments and procedures
As an optional research study, the alternative for patients and caregivers is to not participate in the study.

Adequacy of Protection Against Risks
a. Informed Consent and Assent

A study investigator (PI/sub-I) who is a member of the patient's clinical care team will initiate the conversation with the patient or caregiver identified by the patient about the research trial. If interest is expressed, an investigator or the appropriately delegated Research Nurse/Nurse Practitioner/Physician's Assistant who is also involved in the patient's clinical care will review the consent form with the subject. Each staff member (who does not possess prior experience) who consents participants will shadow the nurse coordinator at least twice to observe the consenting process and familiarize themselves with the research consent. The staff member will then be shadowed by the coordinator at least two times to ensure they are fully capable of conducting the informed consent process.

Only the participant will provide consent or permission. The consent process will occur in a private exam room of the clinic or hospital. The prospective participant will be located behind closed doors to prevent others from potentially over-hearing the conversation. No unauthorized individuals will be present during the consenting process. The usual DUHS standards for patient privacy apply.

Time allotted to review the consent will vary based on the diagnosis and medical need to begin treatment. In general, 1-7 days is given to review consent. Occasionally, more immediate treatment is needed but in all cases, subjects will have time to decide whether or not to participate in the study and all questions will be answered.

There is no limit given on the time for patient discussions. In general, the consenting process takes a minimum of 30-minutes to 1 hour but this will be increased if the potential participant has additional questions and/or concerns about the research. Time for questions is allotted both during the initial conversations with the research physician about the study and during the consenting process. The patient is given both the physician's and research nurse's contact information and instructed that either can be contacted if they have additional questions.

To minimize the possibility of coercion, subjects are told that participating in the study is voluntary and alternative treatment options, including supportive care if appropriate, are discussed with the subject. The voluntary nature of study participation is further emphasized in the consent form and during the consenting process. In addition, the subject is informed that they can withdraw consent at any time.

If the subject cannot read or is blind, the person obtaining consent will read the consent form verbatim to the subject in front of a witness. If the subject decides to participate, they will sign the consent form, or if they are unable to sign, make another kind of mark (like an X) to indicate consent. The person obtaining consent will document at the bottom of the consent form that the consent form was read out loud to the patient by (name of person obtaining consent). If an X or mark is used instead of a signature, the person obtaining consent will note on the consent form that the subject wrote a mark or X instead of a signature. The witness will sign the consent form.

There are no plans to translate the consent form into other languages at this time. Should a non-English speaking potential subject be encountered, the DUHS IRB policy "Research Involving Non-English-Speaking Subjects" will be followed and an amendment for the short form will be submitted. A translator will be used and a witness will be present during the consenting process.

Research involving children

Due to differences in standard of care procedures, this study is restricted to adults.

Waivers

A waiver of consent and HIPAA will be requested for ascertainment purposes.

b. Protections Against Risk

Planned strategies for protecting against or minimizing all potential risks

This is a low risk intervention study that does not involve the use of a study drug or investigational device. No adverse health effects had been established from the use of the iPhone or iWatch. All data will be stored on a secure server, and study personnel will do everything possible to protect patient confidentiality. Nevertheless, there is a potential risk of loss of confidentiality specific to use of mobile applications. These risks are clearly defined within the Informed Consent Form and discussed with the patient at the outset of participation. Information collected by mobile applications or 'apps' is subject to their terms of use, which each participant should read carefully. Many mobile apps that are developed are intended to be very secure, compliant with federal privacy regulations, and used and tested by other academic centers. However, any mobile app that is downloaded carries potential security risks.

To protect privacy, every reasonable effort will be made to prevent undue access to subjects during the course of the study. Prospective participants will be consented in an exam room where it is just the research staff, the patient and his/her family, if desired. For all future visits, interactions with research staff (study doctor and study coordinators) regarding research activities will take place in a private exam room. All research related interactions with the participant will be conducted by qualified research staff who are directly involved in the conduct of the research study.

To protect confidentiality, subject files in paper format will be stored in secure cabinets under lock and key accessible only by the research staff. Only a unique study number and subject initials will identify subjects. Electronic records of subject data will be maintained using a dedicated Microsoft Access database, which is housed in an encrypted and password-protected DCI file server and completed CRFs and demographic information will be stored and updated in REDcap. Access to electronic databases will be limited to study staff and clinical staff supporting the subject's care. The DCI and/or Duke Medicine will manage the security and viability of the IT infrastructure.

Upon completion of the study, research records will be archived and handled per DUHS HRPP policy. Subject names or identifiers will not be used in reports, presentations at scientific meetings, or publications in scientific journals.

Plan for Privacy and Security Protections in the Development and Implementation of Health IT Systems
The subject's gender and age will be collected along with a unique subject ID. The information collected for the registration process, linking a study ID to participants will include first and last name, birthdate, email address and telephone number. This information will be kept on a secure data server approved by the Duke Department of Medicine. Participants may have their own personal health information on devices they own for their own personal use independent of this study. This protocol will not require devices to be used in ways that are different from their regular practice but will ask that participants use numerical or alphanumeric codes to unlock the device. The email addresses supplied by the participants for the purpose of this study will also be on the device. The daily data collected will be kept in a server password protected Citrix ShareFile with encrypted WebDAV access to allow two-way communication between participants and providers via the app. Only study personnel will have access to the data collected. Anonymized data will be shared with other institutions and personnel to allow analysis of the aggregated data. The daily data collected on loaner mobile devices will not include patients' social security number. GPS data will be used to gather contextual atmospheric information.

To reduce the likelihood of any compromise to security, each device is assigned a Universally Unique Identifier, a sequence of 128 bits that can guarantee uniqueness across space and time, defined by RFC 4122. At each study device registration, the device will generate a UUID that is uploaded to a Citrix ShareFile data server and saved on a device keychain. Along with password and user identification match, this unique identifier will have to match the UUID saved on the device. Data saved in the keychain are encrypted. This combination will be required for any access to the data via each study mobile device. Website access to the data server requires a two-step authentication. First, key personnel will enter their user information and password to a website. Once authentication is successful, they will be redirected to another page that requires the input of a six-digit number that is sent to them via SMS to their mobile phone. That number is randomly created for each login attempt and must be provided before data can be accessed. The password will be required to be changed quarterly by key personnel. All data will be encrypted at rest and in transit except when in use after successful authenticated login.

In addition, all incoming or outgoing communication on the data server is encrypted with 256-Bit SSL communication. If the iPad device is lost or stolen, we will be able to erase the device study data remotely, should the subject request this to be done. These data can be done in background mode without the user needing to intervene. Data on the server will not be erased. Patients may track their devices via GPS so that the phone can be located once on-line. Moreover, all devices and the website will be password-protected with unique logins and passwords for all personnel on the project, and the website server is protected by a firewall. Medical record login and access fall under the strict policies of the Duke institution. All study personnel will have received Collaborative Institutional Training Initiative (CITI) training to conduct studies involving human subjects. All key personnel are licensed, bondable, healthcare providers. Patients will be approached in private settings during encounters. Additionally, all IRB approved studies, must have a Research Data Security Plan. This study will continue to conform to the data security plan as described here and as approved by the Duke Institutional Review Board (IRB).

Protections against risks associated with exercise are described above. To review: while there are risks associated with any exercise activity, such as chest pain, shortness of breath, muscle cramping or discomfort, musculoskeletal injury, exacerbation of underlying coronary disease, patients with a known history of at-risk coronary conditions will be excluded. Furthermore, cardiopulmonary exercise testing will be performed as an

additional precaution prior to allowing patients to participate in the exercise intervention. While CPET testing is an exercise itself and has risks, this activity will be performed in a monitored setting by trained professionals. To ensure the utmost safety of our participants going through exercise training, study staff will ensure that all patients who take beta-blockers will have taken their medication prior to training to avoid any blood pressure-related events. Similarly, subjects undergoing interval training will also continue to take their beta-blockers and other medications.

With regard to interval training, the first training session will be performed in a monitored setting by trained professionals; the second session (first session at home) will be performed over a monitored video link by trained professionals, which will also occur weekly the first month, monthly for the remainder of the study, and as needed or requested by study staff or subjects; subjects always have the option to return in person for additional sessions. Therefore, these risks will be mitigated by supervision and training and this intervention is deemed low risk.

<u>Plans for ensuring necessary medical or professional intervention in the event of adverse effects on participants</u>

Initial assessments will take place in person with trained medical staff available to respond either immediately (e.g. CPET testing, interval training) or within 24 hours (e.g. psychological distress brought up by questionnaires/surveys). While adverse events may arise after patients return home, study staff will be available 24 hours a day, seven days a week, to respond to patient concerns and direct them to appropriate care. In fact, the TRU-BMT app and SplendoFit app both contain a tab for contact information and who to call at what time.

<u>Plans for handling incidental findings</u>

Incidental findings related to cardiopulmonary concerns (e.g. CPET testing or interval training) would lead to notification of the primary provider and referral to cardiology or other appropriate services.

Incidental findings related to nutritional status are part of the goal of this study and, the primary provider will be notified.

Incidental findings related to psychological distress, depression, lack of social support, etc. (e.g. raised by questionnaires/surveys) would lead to notification of the primary provider and referral to psychology, psychiatry, social work, or other appropriate services.

Incidental findings related to cognitive function (e.g. raised by questionnaires/surveys) would lead to notification of the primary provider and referral to neuropsychology, occupational therapy, or other appropriate services.

<u>c. Vulnerable Subjects</u>

As this study is built around the Duke Adult Bone Marrow Transplant Program, which only treats patients age 18 and older, fetuses, neonates, and children will be excluded. While subjects may be as old as 80 years, adults with cognitive impairment and unable to consent to participation will not be enrolled. Pregnant women are excluded from transplant and will therefore be excluded from this study. Prisoners and institutionalized individuals are also excluded from this study.

Potential Benefits of the Proposed Research to Research Participants and Others

<u>Potential benefits to research participants</u>

Studies have shown that patients with poor function (e.g. depression, physical frailty) prior to transplant have worse outcomes after transplant (e.g. increased treatment-related mortality). By addressing these functional domains during the pre-transplant period (median 101 days), we have the opportunity to both improve function as well as patient outcomes.

In addition, this study may identify issues pertaining to caregiver distress, leading to the development of further studies targeted at caregivers.

<u>Why the risks to subjects are reasonable in relation to the anticipated benefits</u>

Risks to subjects are reasonable in that risks are low (e.g. related to exercise or psychological distress associated with questionnaire) and the goal of this study is to address these and other risks that may be raised. In fact, it is potentially beneficial to identify and address these risks prior to transplant than during the stress of transplant when they may emerge.

Importance of the Knowledge to be Gained

<u>Importance of the knowledge to be gained</u>

The success of this feasibility study would inform the development of a randomized phase 2 trial of pre-transplant interventions to improve transplant outcomes. This could establish a new standard of care for the treatment of transplant patients.

<u>Why the risks to subjects are reasonable in relation to the importance of the knowledge that reasonably may be expected to result</u>

Treatment-related mortality with allogeneic transplant ranges from 10-30%, with more frail individuals (i.e. functional impairment) at higher risk of adverse events. This study is low risk and has the potential to lead to new interventions to reduce treatment related mortality, which would have a significant impact on health.

7.1 Adverse Events Reporting

While this is a low risk exercise intervention study that does not involve the use of a drug or medical device, the PI will be responsible for the identification and documentation of adverse events and serious adverse events, as defined below. At each study visit or assessment, the PI or designee must assess, through non-suggestive inquiries of the subject or evaluation of study assessments, whether an Adverse Event or Severe Adverse Event has occurred.

An adverse event (AE) is any untoward medical occurrence in a subject receiving study intervention and which does not necessarily have a causal relationship with this treatment. For this protocol, the definition of AE also includes worsening of any pre-existing medical condition. An AE can therefore be any unfavorable and unintended or worsening sign (including an abnormal laboratory finding), symptom, or disease temporally associated with the use of study intervention, whether or not related to use of the study intervention. Abnormal laboratory findings without clinical significance (based on the PI’s judgment) should not be recorded as AEs. But laboratory value changes that require therapy or adjustment in prior therapy are considered adverse events.

Serious adverse events (SAEs) are adverse events that result in any of the following outcomes: death, life-threatening adverse event in the view of the investigator its occurrence places the patient at immediate risk of death, inpatient hospitalization or prolongation of existing hospitalization, a persistent or significant incapacity

or substantial disruption of the ability to conduct normal life functions, or a congenital anomaly/birth defect. All SAEs will be reported to the DUHS IRB within 24 hours for life-threatening events and within 5 business days for non-life-threatening events

From the time the subject signs the informed consent form through the End of Study visit, all AEs/SAEs must be recorded in the subject adverse events case report form. AEs will be assessed according to the CTCAE version 4.0. If CTCAE grading does not exist for an AE, the severity of the AE will be graded as mild (1), moderate (2), severe (3), life-threatening (4), or fatal (5).

In addition to severity, all AEs will be graded as:
- Definite: The AE is clearly related to the study intervention
- Probably: The AE is likely related to the study intervention
- Possible: The AE may be related to the study intervention
- Unlikely: The AE is doubtfully related to the study intervention
- Unrelated: The AE is clearly NOT related to the study intervention

7.2 Serious Adverse Events (SAE)
An AE is considered "serious" if in the opinion of the investigator it is one of the following outcomes:

- Fatal
- Life-threatening
- Constitutes a congenital anomaly or birth defect
- A medically significant condition (defined as an event that compromises subject safety or may require medical or surgical intervention to prevent one of the three outcomes above).
- Requires inpatient hospitalization or prolongation of existing hospitalization
- Results in persistent or significant incapacity or substantial disruption to conduct normal life functions.
-

7.3 Reporting of SAEs
SAEs will be reported to the DUHS IRB within 24 hours, for life-threatening events, and within 5 business days for non-life-threatening events

7.4 Quality Control and Quality Assurance
This is a low risk intervention study that does not involve an agent or device. The study still involves some level of risk due to the subject population and therefore might be subject to safety and monitoring by the DCI SOC and the DCI monitoring team.

7.4.1 Monitoring
This clinical research study will be monitored both internally by the PI and institutionally by the Duke Cancer Institute (DCI). In terms of internal review, the PI will continuously monitor and tabulate adverse events. Appropriate reporting to the Duke University Medical Center IRB will be made. If an unexpected frequency of Grade III or IV events occur, depending on their nature, action appropriate to the nature and frequency of these adverse events will be taken. This may require a protocol amendment, dose de-escalation, or potentially closure of the study. The PI of this study will also continuously monitor the conduct, data, and safety of this study to ensure that:

Risk/benefit ratio is not altered to the detriment of the subjects;
Appropriate internal monitoring of AEs and outcomes is done;
Over-accrual does not occur;
Under-accrual is addressed with appropriate amendments or actions;
Data are being appropriately collected in a reasonably timely manner.

DCI review and monitoring of this protocol occurs in accordance with the NCI-approved Data and Safety Monitoring Plan. Briefly, protocol review begins with an initial review by the Cancer Protocol Committee (CPC), which assesses the ethics and safety of the protocol. Documentation of these assessments will be maintained. Formal, independent monitoring will be conducted by the DCI Monitoring Team after the first 3 subjects are enrolled, followed by annual monitoring of 1-3 subjects until the study is closed to enrollment and subjects are no longer receiving study interventions that are more than minimal risk. DCI Monitoring Team reports and additional data/safety/toxicity reports submitted by the PI will be reviewed by the Safety Oversight Committee (SOC) on an annual basis. Additional monitoring may be prompted by findings from monitoring visits, unexpected frequency of serious and/or unexpected toxicities, or other concerns. Monitoring visits may also be initiated upon request by DUHS and DCI Leadership, CPC, SOC, a sponsor, an investigator, or the IRB.

7.4.2 The Duke Cancer Institute Safety Oversight Committee (SOC)

SOC is responsible for annual data and safety monitoring of DUHS sponsor-investigator phase I and II, therapeutic interventional studies that do not have an independent Data Safety Monitoring Board (DSMB). The primary focus of the SOC is review of safety data, toxicities and new information that may affect subject safety or efficacy. Annual safety reviews includes but may not be limited to review of safety data, enrollment status, stopping rules if applicable, accrual, toxicities, reference literature, and interim analyses as provided by the sponsor-investigator. The SOC in concert with the DCI Monitoring Team oversees the conduct of DUHS cancer-related, sponsor-investigator therapeutic intervention and prevention intervention studies that do not have an external monitoring plan, ensuring subject safety and that the protocol is conducted, recorded and reported in accordance with the protocol, standing operating procedures (SOPs), Good Clinical Practice (GCP), and applicable regulatory requirements.

7.5 Audits

The Duke School of Medicine Clinical Trials Quality Assurance (CTQA) office may conduct confidential audits to evaluate compliance with the protocol and the principles of GCP. The PI agrees to allow the CTQA auditor(s) direct access to all relevant documents and to allocate his/her time and the time of the study team to the CTQA auditor(s) in order to discuss findings and any relevant issues.

CTQA audits are designed to protect the rights and well-being of human research subjects. CTQA audits may be routine or directed (for cause). Routine audits are selected based upon risk metrics generally geared towards high subject enrollment, studies with limited oversight or monitoring, Investigator initiated Investigational Drugs or Devices, federally-funded studies, high degree of risk (based upon adverse events, type of study, or vulnerable populations), Phase I studies, or studies that involve Medicare populations. Directed audits occur at the directive of the IRB or an authorized Institutional Official.

CTQA audits examine research studies/clinical trials methodology, processes and systems to assess whether the research is conducted according to the protocol approved by the DUHS IRB. The primary purpose of the audit/review is to verify that the standards for safety of human subjects in clinical trials and the quality of data produced by the clinical trial research are met. The audit/review will serve as a quality assurance measure, internal to the institution. Additional goals of such audits are to detect both random and systemic errors occurring during the conduct of clinical research and to emphasize “best practices” in the research/clinical trials environment.

The Duke University Office of Audit, Risk, and Compliance – Human Subjects Research Compliance (OARC HSRC) office may conduct confidential audits to evaluate compliance with the protocol and the principles of GCP. The PI agrees to allow the OARC HSRC auditor(s) direct access to all relevant documents and to allocate his/her time and the time of the study team to the OARC HSRC auditor(s) in order to discuss findings and any relevant issues.

8.0 DATA STORAGE AND CONFIDENTIALITY

The Principal Investigator will ensure that subject privacy and confidentiality of the subject's data will be maintained. Research Data Security Plans (RDSPs) will be approved by the appropriate institutional Site Based Research group.

To protect privacy, every reasonable effort will be made to prevent undue access to subjects during the course of the study. Prospective participants will be consented in an exam room where it is just the research staff, the patient and his/her family, if desired. For all future visits, interactions with research staff (study doctor and study coordinators) regarding research activities will take place in a private exam room. All research related interactions with the participant will be conducted by qualified research staff who are directly involved in the conduct of the research study.

To protect confidentiality, subject files in paper format will be stored in secure cabinets under lock and key accessible only by the research staff. Only a unique study number and subject initials will identify subjects. Electronic records of subject data will be maintained using a dedicated Microsoft Access database, which is housed in an encrypted and password-protected DCI file server and completed CRFs and demographic information will be stored and updated in REDcap and Duke Box. Access to electronic databases will be limited to study staff and clinical staff supporting the subject's care. The DCI and/or Duke Medicine will manage the security and viability of the IT infrastructure.

Upon completion of the study, research records will be archived and handled per DUHS HRPP policy.

Subject names or identifiers will not be used in reports, presentations at scientific meetings, or publications in scientific journals.

8.1 Mobile Application and Duke-Loaned Device Confidentiality

8.1.1 Risk/Benefit Assessment

There are no known risks associated with this study. No adverse health effects had been established from the use of the iPhone or iWatch. All data will be stored on a secure server, and study personnel will do everything possible to protect patient confidentiality. Nevertheless, there is a potential risk of loss of confidentiality specific to use of mobile applications. These risks are clearly defined within the Informed Consent Form and discussed with the patient at the outset of participation, as described below.

8.1.2 Risks Specific to Mobile Applications

Information collected by mobile applications or 'apps' is subject to their terms of use, which each participant should read carefully. Many mobile apps that are developed are intended to be very secure, compliant with federal privacy regulations, and used and tested by other academic centers. However, any mobile app that is downloaded carries potential security risks. The mobile apps for the current study were developed in conjunction with other Duke investigators (i.e. Dr. Nirmish Shah and Sophia Smith, PhD), and efforts to limit potential security risks associated with mobile applications have been made; however, Duke cannot guarantee that the mobile apps are free of risk.

The TRU-BMT application have been programmed to access only certain features of the participant's device (camera, microphone) that will enable study activities. Other applications on the device will not have access to data entered into the study app. Information that is entered into the mobile apps that is stored outside of Duke is stored on a server, with authorized access by only the study team. Sicklesoft may store or use data stored on cloud servers managed or hosted by Microsoft, Google and its affiliates, Apple, AWS, and/or Sharefile as appropriate. Each participant will be asked to use an email address to set up his or her account within the mobile app; if preferred, the study team will provide an email for the purpose of the study. Other identifying information entered into the app will be stored confidentially as described below.

The SplendoFit app used industry standard SSL encryption. All Google Cloud components used by the platform store data with encryption-at-rest principles (AES256 based). The monitor app runs on a remotely managed iPad which enforces strong user credentials and encrypts the entire file system (using AES256) based on those. Identity platform stores passwords using the scrypt algorithm. Encryption keys and secrets can be used on the platform to communicate with 3rd parties or create additional layers of encryption within the platform. To protect the content of these keys and secrets, access to them is guarded by only providing certain (service) accounts access to them, using the unified IAM model from Google Cloud. Additional information about security and complicant for the SplendoFit app can be found here: https://splendo.health/privacy/ .

Patients enrolled in the study will be provided a device (e.g., iPad mini, iPhone) with the study software. However, patients and their families may elect to use their own devices. Patients will be instructed to run a current operating system (OS) on their device, review the privacy/security settings often, and restrict any unnecessary access. The application may run in the background of their device. Mobile apps may have unanticipated impact on the operations of the device (e.g., battery drainage). If the patient does not have an unlimited data/text plan, s/he may incur additional charges. At the conclusion of the study, the study team will provide participants with instructions on how to remove the mobile apps from their personal device.

While this app is intended to help monitor symptoms, track physical activity and food group intake, provide encouragement to maintain or improve adherence and self-care, and inform provider decision-making, the apps are not intended to supplant healthcare decisions discussed with the patient's healthcare provider.

As with all technology, we will ask patients and their families to wait until they are in a safe environment, use good judgment, and follow prevailing laws. While the devices are intended to only be used in the participant homes, patients and their families should not perform study-related activities while they are driving.

8.1.3 Use of Duke-loaned Devices

If patients are loaned a Duke device for use during this study and it is used for non-study related reasons, this could add additional personal information onto the device and potentially result in it being sent to unauthorized persons. The Duke-loaned device will be preset with security settings and patients will be asked not alter these during the course of the study. When the device is returned at the end of the study, the device will be cleaned to remove any personal information. If the device is lost or stolen during the course of the study, patients will be instructed to contact the study team immediately.

8.1.4 Potential Problems with Mobile Applications

In order to ensure that patient-reported variables are known to the health care team and research staff, all updates made by the study apps (and other information on the app interface) will be able to be viewed by providers (nurses and physician staff) on a data dashboard.

We do not expect that there will be technical difficulty with the use of TRU-BMT app or SplendoFit app. The TRU-BMT app has already been piloted in Duke HCT patients (Pro00068979, PI Shah, co-I Sung) and was rated favorably as both easy to use and helpful (abstract presentation at the American Society of Hematology 2017 Annual Meeting).

8.1.5 Plan for Privacy and Security Protections in the Development and Implementation of Health IT Systems

The subject's gender and age will be collected along with a unique subject ID. The information collected for the registration process, linking a study ID to participants will include first and last name, birthdate, email address and telephone number. This information will be kept on a secure data server approved by the Duke Department of Medicine. Participants may have their own personal health information on devices they own for their own personal use independent of this study. This protocol will not require devices to be used in ways that are different from their regular practice but will ask that participants use numerical or alphanumeric codes to unlock the device. The email addresses supplied by the participants for the purpose of this study will also be on the device. The daily data collected will be kept in a server password protected Citrix ShareFile with encrypted WebDAV access to allow two-way communication between participants and providers via the app. Only study personnel will have access to the data collected. Anonymized data will be shared with other institutions and personnel to allow analysis of the aggregated data. The daily data collected on loaner mobile devices will not include patients' social security number. GPS data will be used to gather contextual atmospheric information.

To reduce the likelihood of any compromise to security, each device is assigned a Universally Unique Identifier, a sequence of 128 bits that can guarantee uniqueness across space and time, defined by RFC 4122. At each study device registration, the device will generate a UUID that is uploaded to a Citrix ShareFile data server and saved on a device keychain. Along with password and user identification match, this unique identifier will have to match the UUID saved on the device. Data saved in the keychain are encrypted. This combination will be required for any access to the data via each study mobile device. Website access to the data server requires a two-step authentication. First, key personnel will enter their user information and password to a website. Once authentication is successful, they will be redirected to another page that requires the input of a six-digit number that is sent to them via SMS to their mobile phone. That number is randomly created for each login attempt and must be provided before data can be accessed. The password will be required to be changed quarterly by key personnel. All data will be encrypted at rest and in transit except when in use after successful authenticated login.

In addition, all incoming or outgoing communication on the data server is encrypted with 256-Bit SSL communication. If the iPad device is lost or stolen, we will be able to erase the device study data remotely, should the subject request this to be done. These data can be done in background mode without the user needing to intervene. Data on the server will not be erased. Patients may track their devices via GPS so that the phone can be located once on-line. Moreover, all devices and the website will be password-protected with unique logins and passwords for all personnel on the project, and the website server is protected by a firewall. Medical record login and access fall under the strict policies of the Duke institution. All study personnel will have received Collaborative Institutional Training Initiative (CITI) training to conduct studies involving human subjects. All key personnel are licensed, bondable, healthcare providers. Patients will be approached in private settings during encounters. Additionally, all IRB approved studies, must have a Research Data Security Plan. This study will continue to conform to the data security plan as described here and as approved by the Duke Institutional Review Board (IRB).

8.2 Records Retention

The Principal Investigator will maintain study-related records for the longer of a period of:

- at least two years after the date on which a New Drug Application is approved by the FDA (if an IND is involved)
- at least two years after formal withdrawal of the IND associated with this protocol (if an IND is involved)
- at least six years after study completion (Duke policy)

9.0 DATA MANAGEMENT AND PROCESSING

9.1 Study Documentation

Study documentation includes but is not limited to source documents, case report forms, monitoring logs, appointment schedules, study team correspondence with sponsors or regulatory bodies/committees, and regulatory documents that can be found in the DCI-mandated “Regulatory Binder”, which includes but is not limited to signed protocol and amendments, approved and signed informed consent forms, CAP and CLIA laboratory certifications, and clinical supplies receipts and distribution records.

Source documents are original records that contain source data, which is all information in original records of clinical findings, observations, or other activities in a clinical trial necessary for the reconstruction and evaluation of the trial. Source documents include but are not limited to hospital records, clinical and office charts, laboratory notes, memoranda, subjects’ diaries or evaluation checklists, pharmacy dispensing records, recorded data from automated instruments, x-rays, subject files, billing records (for cost impact and economic analysis) and records kept at the laboratories and at medico-technical departments involved in the clinical trial. When possible, the original record should be retained as the source document. However, a photocopy is acceptable provided that it is a clear, legible, and an exact duplication of the original document.

9.2 Case Report Forms (CRFs)

This study will utilize electronic Case Report Forms eCRFs in a REDCap database for data reporting. REDCap is a software tool that does not require client local software and can be accessed from anywhere on the Internet and is secured on a Duke Health Technology Services (DHTS) server. This database will be developed and maintenance performed with support of the School of Medicine (SOM) Duke Office of Clinical Research (DOCR). SOM’s DOCR has partnered with the School of Medicine (SOM) to implement REDCap (developed by Vanderbilt’s CTSA and currently used and supported by more than 1600 consortium partners. REDCap provides: 1) a stream-lined process for rapidly building a database; 2) an intuitive interface for collecting data (with data validation and audit trail); 3) automated export procedures for seamless data downloads to common statistical packages (SAS, SPSS, etc.); 4) branching logic, file uploading, and calculated fields; and 5) a quick and easy protocol set-up.

REDCap accounts are stored within the DTMI LDAP server hosted by the Duke Office of Information Technology (OIT). Authentication occurs via the OIT implementation of Kerberos. All connections to the system, both external and internal, occur over encrypted channels. Access to components of the system is role-based and can only be granted by administrators of the system. All collected information is stored on a database server hosted by Duke Health Technology Services (DHTS). The database server resides behind the DHTS internal firewall and access to the server is controlled via firewall rules. All collected data are backed up daily, both on the local server and by the DHTS enterprise backup system. The Office of Research Informatics-App Engineering-DHTS (ORI) via ServiceNow (919 668-7286) is responsible for managing the server for REDCap. Duke Office of Clinical Research (Ceci Chamorro 919-668-9262) is responsible for managing the database platform for REDCap. At the time of this submission, REDCap is on version 6.5.15.19

The eCRFs will be the primary data collection documents for this study. eCRFs will be updated in a timely manner following acquisition of new source data.

9.3 Data Management Procedures and Data Verification

Data collected for the study and entered into the RedCap database will be verified against original source documents. Source documentation for study data points may be redacted, labeled with the subjects' study ID, and sent to the coordinating site via email or fax. This source documentation will be filed in individual subject study binders for verification at monitoring visits and study audits.

Alternatively, source documentation may be verified remotely through direct access to the electronic medical record via secure methods approved by Duke and the PI of the study.

9.4 Coding

All medical terms will be coded with MedDRA (Medical Dictionary for Regulatory Activities). Medication will be coded according to the World Health Organization Drug Dictionary.

9.5 Study Closure

Following completion of the study, the PI will be responsible for ensuring the following activities:

- **a.** Data clarification and/or resolution
- **b.** Accounting, reconciliation, and destruction/return of used and unused study drugs
- **c.** Review of site study records for completeness
- **d.** Shipment of all remaining laboratory samples to the designated laboratories

10.0 ADMINISTRATIVE AND ETHICAL CONSIDERATIONS

10.1 Regulatory and Ethical Compliance

This protocol was designed and will be conducted and reported in accordance with the International Conference on Harmonization (ICH) Harmonized Tripartite Guidelines for Good Clinical Practice, the Declaration of Helsinki, and applicable federal, state, and local regulations.

10.2 DUHS Institutional Review Board and DCI Cancer Protocol Committee

The protocol, informed consent form, advertising material, and additional protocol-related documents must be submitted to the DUHS Institutional Review Board (IRB) and DCI Cancer Protocol Committee (CPC) for review. The study may be initiated only after the Principal Investigator has received written and dated approval from the CPC and IRB.

The Principal Investigator must submit and obtain approval from the IRB for all subsequent protocol amendments and changes to the informed consent form. The CPC should be informed about any protocol amendments that potentially affect research design or data analysis (i.e. amendments affecting subject population, inclusion/exclusion criteria, agent administration, statistical analysis, etc.).

The Principal Investigator must obtain protocol re-approval from the IRB within 1 year of the most recent IRB approval. The Principal Investigator must also obtain protocol re-approval from the CPC within 1 year of the most recent IRB approval, for as long as the protocol remains open to subject enrollment.

10.3 Informed Consent

The informed consent form must be written in a manner that is understandable to the subject population. Prior to its use, the informed consent form must be approved by the IRB.

The Principal Investigator or authorized key personnel will discuss with the potential subject the purpose of the research, methods, potential risks and benefits, subject concerns, and other study-related matters. This discussion will occur in a location that ensures subject privacy and in a manner that minimizes the possibility of coercion. Appropriate accommodations will be made available for potential subjects who cannot read or understand English or are visually impaired. Potential subjects will have the opportunity to contact the Principal investigator or authorized key personnel with questions, and will be given as much time as needed to make an informed decision about participation in the study.

Before conducting any study-specific procedures, the Principal Investigator must obtain written informed consent from the subject. The original informed consent form will be stored with the subject’s study records, and a copy of the informed consent form will be provided to the subject. The Principal Investigator is responsible for asking the subject whether the subject wishes to notify his/her primary care physician about participation in the study. If the subject agrees to such notification, the Principal Investigator will inform the subject’s primary care physician about the subject’s participation in the clinical study.

10.4 Protocol Amendments

All protocol amendments must be initiated by the Principal Investigator and approved by the IRB prior to implementation. IRB approval is not required for protocol changes that occur to protect the safety of a subject from an immediate hazard. However, the Principal Investigator must inform the IRB and all other applicable regulatory agencies of such action immediately.

Though not yet required, the CPC should be informed about any protocol amendments that potentially affect research design or data analysis (i.e. amendments affecting subject population, inclusion/exclusion criteria, agent administration, etc.).

11.0 REFERENCES

1. Sherman AC, Simonton S, Latif U, Plante TG, Anaissie EJ. Changes in quality-of-life and psychosocial adjustment among multiple myeloma patients treated with high-dose melphalan and autologous stem cell transplantation. Biology of blood and marrow transplantation : journal of the American Society for Blood and Marrow Transplantation 2009;15:12-20.
2. Mosher CE, Redd WH, Rini CM, Burkhalter JE, DuHamel KN. Physical, psychological, and social sequelae following hematopoietic stem cell transplantation: a review of the literature. Psychooncology 2009;18:113-27.
3. Danaher EH, Ferrans C, Verlen E, et al. Fatigue and physical activity in patients undergoing hematopoietic stem cell transplant. Oncology nursing forum 2006;33:614-24.
4. Andersson I, Ahlberg K, Stockelberg D, Brune M, Persson LO. Health-related quality of life in patients undergoing allogeneic stem cell transplantation after reduced intensity conditioning versus myeloablative conditioning. Cancer nursing 2009;32:325-34.
5. Anderson KO, Giralt SA, Mendoza TR, et al. Symptom burden in patients undergoing autologous stem-cell transplantation. Bone Marrow Transplant 2007;39:759-66.
6. Current Uses and Outcomes of Hematopoietic Cell Transplantation (HCT): CIBMTR Summary Slides, 2016. 2016. (Accessed October 24, 2017, at Available at: http://www.cibmtr.org.)
7. Broers S, Kaptein AA, Le Cessie S, Fibbe W, Hengeveld MW. Psychological functioning and quality of life following bone marrow transplantation: a 3-year follow-up study. J Psychosom Res 2000;48:11-21.
8. Fearon K, Strasser F, Anker SD, et al. Definition and classification of cancer cachexia: an international consensus. The Lancet Oncology 2011;12:489-95.
9. Dimeo F, Bertz H, Finke J, Fetscher S, Mertelsmann R, Keul J. An aerobic exercise program for patients with haematological malignancies after bone marrow transplantation. Bone Marrow Transplant 1996;18:1157-60.
10. DeFor TE, Burns LJ, Gold E-MA, Weisdorf DJ. A Randomized Trial of the Effect of a Walking Regimen on the Functional Status of 100 Adult Allogeneic Donor Hematopoietic Cell Transplant Patients. Biology of Blood and Marrow Transplantation 2007;13:948-55.
11. Jarden M, Baadsgaard MT, Hovgaard DJ, Boesen E, Adamsen L. A randomized trial on the effect of a multimodal intervention on physical capacity, functional performance and quality of life in adult patients undergoing allogeneic SCT. Bone Marrow Transplant 2009;43:725-37.
12. Jarden M, Hovgaard D, Boesen E, Quist M, Adamsen L. Pilot study of a multimodal intervention: mixed-type exercise and psychoeducation in patients undergoing allogeneic stem cell transplantation. Bone Marrow Transplant 2007;40:793-800.
13. Wiskemann J, Huber G. Physical exercise as adjuvant therapy for patients undergoing hematopoietic stem cell transplantation. Bone Marrow Transplant 2007;41:321-9.
14. Baumann FT, Kraut L, Schule K, Bloch W, Fauser AA. A controlled randomized study examining the effects of exercise therapy on patients undergoing haematopoietic stem cell transplantation. Bone Marrow Transplant 2010;45:355-62.
15. Baumann FT, Zopf EM, Nykamp E, et al. Physical activity for patients undergoing an allogeneic hematopoietic stem cell transplantation: benefits of a moderate exercise intervention. European Journal of Haematology 2011;87:148-56.
16. Wiskemann J, Dreger P, Schwerdtfeger R, et al. Effects of a partly self-administered exercise program before, during, and after allogeneic stem cell transplantation. Blood 2011;117:2604-13.
17. Lee Y, Kim J, Chon D, et al. The effects of frailty and cognitive impairment on 3-year mortality in older adults. Maturitas;107:50-5.

18. Libert Y, Dubruille S, Borghgraef C, et al. Vulnerabilities in Older Patients when Cancer Treatment is Initiated: Does a Cognitive Impairment Impact the Two-Year Survival? PLOS ONE 2016;11:e0159734.
19. Silberfarb PM. Chemotherapy and cognitive defects in cancer patients. Annual review of medicine 1983;34:35-46.
20. Janelsins MC, Kohli S, Mohile SG, Usuki K, Ahles TA, Morrow GR. An update on cancer- and chemotherapy-related cognitive dysfunction: current status. Seminars in oncology 2011;38:431-8.
21. Pedersen AD, Rossen P, Mehlsen MY, Pedersen CG, Zachariae R, von der Maase H. Long-term cognitive function following chemotherapy in patients with testicular cancer. Journal of the International Neuropsychological Society : JINS 2009;15:296-301.
22. Vardy J, Tannock I. Cognitive function after chemotherapy in adults with solid tumours. Critical reviews in oncology/hematology 2007;63:183-202.
23. Andrykowski M, Altmaier E, Barnett R, Burish T, Gingrich R, Henslee-Downey P. Cognitive dysfunction in adult survivors of allogeneic marrow transplantation: relationship to dose of total body irradiation. Bone marrow transplantation 1990;6:269-76.
24. Harder H, Cornelissen JJ, Van Gool AR, Duivenvoorden HJ, Eijkenboom WM, van den Bent MJ. Cognitive functioning and quality of life in long-term adult survivors of bone marrow transplantation. Cancer 2002;95:183-92.
25. Jones D, Vichaya EG, Wang XS, Sailors MH, Cleeland CS, Wefel JS. Acute cognitive impairment in patients with multiple myeloma undergoing autologous hematopoietic stem cell transplant. Cancer 2013;119:4188-95.
26. Meyers CA, Weitzner M, Byrne K, Valentine A, Champlin RE, Przepiorka D. Evaluation of the neurobehavioral functioning of patients before, during, and after bone marrow transplantation. Journal of clinical oncology : official journal of the American Society of Clinical Oncology 1994;12:820-6.
27. Syrjala KL, Dikmen S, Langer SL, Roth-Roemer S, Abrams JR. Neuropsychologic changes from before transplantation to 1 year in patients receiving myeloablative allogeneic hematopoietic cell transplant. Blood 2004;104:3386-92.
28. Booth-Jones M, Jacobsen PB, Ransom S, Soety E. Characteristics and correlates of cognitive functioning following bone marrow transplantation. Bone Marrow Transplantation 2005;36:695-702.
29. Braamse AMJ, Gerrits MMJG, van Meijel B, et al. Predictors of health-related quality of life in patients treated with auto- and allo-SCT for hematological malignancies. Bone Marrow Transplant 2012;47:757-69.
30. Andorsky DJ, Loberiza FR, Lee SJ. Pre-transplantation physical and mental functioning is strongly associated with self-reported recovery from stem cell transplantation. Bone Marrow Transplantation 2006;37:889-95.
31. Ehrlich KB, Miller GE, Scheide T, et al. Pre-transplant emotional support is associated with longer survival after allogeneic hematopoietic stem cell transplantation. Bone Marrow Transplant 2016;51:1594-8.
32. Fife BL, Huster GA, Cornetta KG, Kennedy VN, Akard LP, Broun ER. Longitudinal study of adaptation to the stress of bone marrow transplantation. Journal of clinical oncology : official journal of the American Society of Clinical Oncology 2000;18:1539-49.
33. El-Jawahri A, Chen Y-B, Brazauskas R, et al. Impact of pre-transplant depression on outcomes of allogeneic and autologous hematopoietic stem cell transplantation. Cancer 2017;123:1828-38.
34. Brotelle T, Lemal R, Cabrespine A, et al. Prevalence of malnutrition in adult patients previously treated with allogeneic hematopoietic stem-cell transplantation. Clinical nutrition (Edinburgh, Scotland) 2017.
35. Horsley P, Bauer J, Gallagher B. Poor nutritional status prior to peripheral blood stem cell transplantation is associated with increased length of hospital stay. Bone Marrow Transplantation 2005;35:1113-6.
36. Krawczyk J, Kraj L, Korta T, Wiktor-Jedrzejczak W. Nutritional Status of Hematological Patients before Hematopoietic Stem Cell Transplantation and in Early Posttransplantation Period. Nutrition and cancer 2017:1-6.

37. Greenfield RM. Reflections on leukemia and adult stem-cell transplantation: some personal psychological factors. Palliative & supportive care 2007;5:415-8.
38. Kettmann JDJ, Altmaier EM. Social Support and Depression among Bone Marrow Transplant Patients. Journal of Health Psychology 2008;13:39-46.
39. Hochhausen N, Altmaier EM, McQuellon R, et al. Social Support, Optimism, and Self-Efficacy Predict Physical and Emotional Well-Being After Bone Marrow Transplantation. Journal of Psychosocial Oncology 2007;25:87-101.
40. Frick E, Ramm G, Bumeder I, et al. Social support and quality of life of patients prior to stem cell or bone marrow transplantation. British journal of health psychology 2006;11:451-62.
41. Jacobsen PB, Sadler IJ, Booth-Jones M, Soety E, Weitzner MA, Fields KK. Predictors of posttraumatic stress disorder symptomatology following bone marrow transplantation for cancer. Journal of consulting and clinical psychology 2002;70:235-40.
42. Rodrigue JR, Pearman TP, Moreb J. Morbidity and mortality following bone marrow transplantation: Predictive utility of pre-BMT affective functioning, compliance, and social support stability. International Journal of Behavioral Medicine 1999;6:241-54.
43. Wells KJ, Booth-Jones M, Jacobsen PB. Do Coping and Social Support Predict Depression and Anxiety in Patients Undergoing Hematopoietic Stem Cell Transplantation? Journal of Psychosocial Oncology 2009;27:297-315.
44. Widows MR, Jacobsen PB, Booth-Jones M, Fields KK. Predictors of posttraumatic growth following bone marrow transplantation for cancer. Health psychology : official journal of the Division of Health Psychology, American Psychological Association 2005;24:266-73.
45. Foster LW, McLellan L, Rybicki L, Dabney J, Copelan E, Bolwell B. Validating the positive impact of in-hospital lay care-partner support on patient survival in allogeneic BMT: a prospective study. Bone Marrow Transplant 2013;48:671-7.
46. Grimm PM, Zawacki KL, Mock V, Krumm S, Frink BB. Caregiver responses and needs. An ambulatory bone marrow transplant model. Cancer practice 2000;8:120-8.
47. Simoneau TL, Mikulich-Gilbertson SK, Natvig C, et al. Elevated peri-transplant distress in caregivers of allogeneic blood or marrow transplant patients. Psychooncology 2013;22:2064-70.
48. Aslan O, Kav S, Meral C, et al. Needs of lay caregivers of bone marrow transplant patients in Turkey: a multicenter study. Cancer nursing 2006;29:E1-7.
49. Eldredge DH, Nail LM, Maziarz RT, Hansen LK, Ewing D, Archbold PG. Explaining family caregiver role strain following autologous blood and marrow transplantation. J Psychosoc Oncol 2006;24:53-74.
50. Applebaum AJ, Bevans M, Son T, et al. A scoping review of caregiver burden during allogeneic HSCT: lessons learned and future directions. Bone Marrow Transplant 2016;51:1416-22.
51. Laudenslager ML, Simoneau TL, Kilbourn K, et al. A randomized control trial of a psychosocial intervention for caregivers of allogeneic hematopoietic stem cell transplant patients: effects on distress. Bone Marrow Transplant 2015;50:1110-8.
52. Karczewski J, Poniedzialek B, Adamski Z, Rzymski P. The effects of the microbiota on the host immune system. Autoimmunity 2014;47:494-504.
53. Neish AS. Microbes in gastrointestinal health and disease. Gastroenterology 2009;136:65-80.
54. Lawley TD, Walker AW. Intestinal colonization resistance. Immunology 2013;138:1-11.
55. Morgan XC, Tickle TL, Sokol H, et al. Dysfunction of the intestinal microbiome in inflammatory bowel disease and treatment. Genome biology 2012;13:R79.
56. de Castro CG, Jr., Ganc AJ, Ganc RL, Petrolli MS, Hamerschlack N. Fecal microbiota transplant after hematopoietic SCT: report of a successful case. Bone Marrow Transplant 2015;50:145.
57. Lee JR, Muthukumar T, Dadhania D, et al. Gut microbial community structure and complications after kidney transplantation: a pilot study. Transplantation 2014;98:697-705.
58. Oh PL, Martinez I, Sun Y, Walter J, Peterson DA, Mercer DF. Characterization of the ileal microbiota in rejecting and nonrejecting recipients of small bowel transplants. American journal of transplantation : official

journal of the American Society of Transplantation and the American Society of Transplant Surgeons 2012;12:753-62.
59. Taur Y, Jenq RR, Perales MA, et al. The effects of intestinal tract bacterial diversity on mortality following allogeneic hematopoietic stem cell transplantation. Blood 2014;124:1174-82.
60. Wu ZW, Ling ZX, Lu HF, et al. Changes of gut bacteria and immune parameters in liver transplant recipients. Hepatobiliary & pancreatic diseases international : HBPD INT 2012;11:40-50.
61. Lu H, He J, Wu Z, et al. Assessment of microbiome variation during the perioperative period in liver transplant patients: a retrospective analysis. Microbial ecology 2013;65:781-91.
62. Holler E, Butzhammer P, Schmid K, et al. Metagenomic analysis of the stool microbiome in patients receiving allogeneic stem cell transplantation: loss of diversity is associated with use of systemic antibiotics and more pronounced in gastrointestinal graft-versus-host disease. Biology of blood and marrow transplantation : journal of the American Society for Blood and Marrow Transplantation 2014;20:640-5.
63. Taur Y, Xavier JB, Lipuma L, et al. Intestinal domination and the risk of bacteremia in patients undergoing allogeneic hematopoietic stem cell transplantation. Clinical infectious diseases : an official publication of the Infectious Diseases Society of America 2012;55:905-14.
64. Jagasia M, Arora M, Flowers ME, et al. Risk factors for acute GVHD and survival after hematopoietic cell transplantation. Blood 2012;119:296-307.
65. Paczesny S, Krijanovski OI, Braun TM, et al. A biomarker panel for acute graft-versus-host disease. Blood 2009;113:273.
66. Dalle-Donne I, Rossi R, Giustarini D, Milzani A, Colombo R. Protein carbonyl groups as biomarkers of oxidative stress. Clinica chimica acta; international journal of clinical chemistry 2003;329:23-38.
67. Inglés M, Gambini J, Carnicero JA, et al. Oxidative Stress Is Related to Frailty, Not to Age or Sex, in a Geriatric Population: Lipid and Protein Oxidation as Biomarkers of Frailty. Journal of the American Geriatrics Society 2014;62:1324-8.
68. Terrando N, Monaco C, Ma D, Foxwell BMJ, Feldmann M, Maze M. Tumor necrosis factor-α triggers a cytokine cascade yielding postoperative cognitive decline. Proceedings of the National Academy of Sciences 2010;107:20518-22.
69. Chen Y, Jungsuwadee P, Vore M, Butterfield DA, St Clair DK. Collateral damage in cancer chemotherapy: oxidative stress in nontargeted tissues. Molecular interventions 2007;7:147-56.
70. Conklin KA. Chemotherapy-associated oxidative stress: impact on chemotherapeutic effectiveness. Integrative cancer therapies 2004;3:294-300.
71. Nakazato T, Ito C, Shimazaki K, Arakaki H, Aisa Y. Evaluation of Oxidative Stress Markers in Hematopoietic Stem Cell Transplantation Patients. Am Soc Hematology; 2015.
72. Gunther ML, Morandi A, Ely EW. Pathophysiology of Delirium in the Intensive Care Unit. Critical Care Clinics 2008;24:45-65.
73. van Zyl LT, Seitz DP. Delirium concisely: condition is associated with increased morbidity, mortality, and length of hospitalization. Geriatrics 2006;61:18-21.
74. Centeno C, Sanz A, Bruera E. Delirium in advanced cancer patients. Palliative medicine 2004;18:184-94.
75. Pereira J, Hanson J, Bruera E. The frequency and clinical course of cognitive impairment in patients with terminal cancer. Cancer 1997;79:835-42.
76. Massie MJ, Holland J, Glass E. Delirium in terminally ill cancer patients. The American journal of psychiatry 1983;140:1048-50.
77. Fann JR, Roth-Roemer S, Burington BE, Katon WJ, Syrjala KL. Delirium in patients undergoing hematopoietic stem cell transplantation. Cancer 2002;95:1971-81.
78. Binder DK, Scharfman HE. Brain-derived Neurotrophic Factor. Growth factors (Chur, Switzerland) 2004;22:123-31.

79. Grandi C, Tomasi CD, Fernandes K, et al. Brain-derived neurotrophic factor and neuron-specific enolase, but not S100β, levels are associated to the occurrence of delirium in intensive care unit patients. Journal of Critical Care 2011;26:133-7.
80. Vasunilashorn SM, Ngo L, Inouye SK, et al. Cytokines and Postoperative Delirium in Older Patients Undergoing Major Elective Surgery. The Journals of Gerontology Series A: Biological Sciences and Medical Sciences 2015;70:1289-95.
81. Wiskemann J, Kuehl R, Dreger P, et al. Physical Exercise Training versus Relaxation in Allogeneic stem cell transplantation (PETRA Study) – Rationale and design of a randomized trial to evaluate a yearlong exercise intervention on overall survival and side-effects after allogeneic stem cell transplantation. BMC Cancer 2015;15:619.
82. mHealth: New horizons for health through mobile technologies: second global survey on eHealth. 2011. (Accessed November 1, 2017, at http://www.who.int/goe/publications/goe_mhealth_web.pdf.)
83. Reddon JR, Stefanyk WO, Gill DM, Renney C. Hand dynamometer: effects of trials and sessions. Perceptual and motor skills 1985;61:1195-8.
84. Balogun JA, Akomolafe CT, Amusa LO. Grip strength: effects of testing posture and elbow position. Archives of physical medicine and rehabilitation 1991;72:280-3.
85. Kallman DA, Plato CC, Tobin JD. The role of muscle loss in the age-related decline of grip strength: cross-sectional and longitudinal perspectives. Journal of gerontology 1990;45:M82-8.
86. Mathiowetz V, Rennells C, Donahoe L. Effect of elbow position on grip and key pinch strength. The Journal of hand surgery 1985;10:694-7.
87. Trossman PB, Suleski KB, Li P-W. Test-Retest Reliability and Day-to-Day Variability of an Isometric Grip Strength Test Using the Work Simulator. The Occupational Therapy Journal of Research 1990;10:266-79.
88. Jones LW, Courneya KS, Mackey JR, et al. Cardiopulmonary function and age-related decline across the breast cancer survivorship continuum. Journal of clinical oncology : official journal of the American Society of Clinical Oncology 2012;30:2530-7.
89. Martin EA, Battaglini CL, Hands B, Naumann F. Higher-Intensity Exercise Results in More Sustainable Improvements for VO2peak for Breast and Prostate Cancer Survivors. Oncology nursing forum 2015;42:241-9.
90. Randolph C, Tierney MC, Mohr E, Chase TN. The Repeatable Battery for the Assessment of Neuropsychological Status (RBANS): preliminary clinical validity. J Clin Exp Neuropsychol 1998;20:310-9.
91. Terachi S, Yamada T, Pu S, Yokoyama K, Matsumura H, Kaneko K. Comparison of neurocognitive function in major depressive disorder, bipolar disorder, and schizophrenia in later life: A cross-sectional study of euthymic or remitted, non-demented patients using the Japanese version of the Brief Assessment of Cognition in Schizophrenia (BACS-J). Psychiatry Res 2017;254:205-10.
92. Batty RA, Francis A, Thomas N, Hopwood M, Ponsford J, Rossell SL. A brief neurocognitive assessment of patients with psychosis following traumatic brain injury (PFTBI): Use of the Repeatable battery for the Assessment of Neuropsychological Status (RBANS). Psychiatry Res 2016;237:27-36.
93. Taichman DB, Christie J, Biester R, et al. Validation of a brief telephone battery for neurocognitive assessment of patients with pulmonary arterial hypertension. Respir Res 2005;6:39.
94. Gogos A, Joshua N, Rossell SL. Use of the Repeatable Battery for the Assessment of Neuropsychological Status (RBANS) to investigate group and gender differences in schizophrenia and bipolar disorder. Aust N Z J Psychiatry 2010;44:220-9.
95. Smith PJ, Rivelli S, Waters A, et al. Neurocognitive changes after lung transplantation. Annals of the American Thoracic Society 2014;11:1520-7.
96. Misdraji EL, Gass CS. The Trail Making Test and its neurobehavioral components. J Clin Exp Neuropsychol 2010;32:159-63.
97. Duff K, Hobson VL, Beglinger LJ, O'Bryant SE. Diagnostic Accuracy of the RBANS in Mild Cognitive Impairment: Limitations on Assessing Milder Impairments. Archives of Clinical Neuropsychology 2010;25:429-41.

98. Cella D, Riley W, Stone A, et al. The Patient-Reported Outcomes Measurement Information System (PROMIS) developed and tested its first wave of adult self-reported health outcome item banks: 2005-2008. Journal of clinical epidemiology 2010;63:1179-94.
99. Sherman RS, Cooke E, Grant M. Dialogue among survivors of hematopoietic cell transplantation support-group themes. J Psychosoc Oncol 2005;23:1-24.
100. Lounsberry JJ, Macrae H, Angen M, Hoeber M, Carlson LE. Feasibility study of a telehealth delivered, psychoeducational support group for allogeneic hematopoietic stem cell transplant patients. Psychooncology 2010;19:777-81.
101. Widows MR, Jacobsen PB, Fields KK. Relation of psychological vulnerability factors to posttraumatic stress disorder symptomatology in bone marrow transplant recipients. Psychosomatic medicine 2000;62:873-82.
102. Esser P, Kuba K, Scherwath A, et al. Posttraumatic stress disorder symptomatology in the course of allogeneic HSCT: a prospective study. Journal of Cancer Survivorship 2017;11:203-10.
103. PTSD: National Center for PTSD. (Accessed December 20, 2017, at https://www.ptsd.va.gov/professional/assessment/adult-sr/ptsd-checklist.asp.)
104. Blevins CA, Weathers FW, Davis MT, Witte TK, Domino JL. The Posttraumatic Stress Disorder Checklist for DSM-5 (PCL-5): Development and Initial Psychometric Evaluation. Journal of Traumatic Stress 2015;28:489-98.
105. Bauer J, Capra S, Ferguson M. Use of the scored Patient-Generated Subjective Global Assessment (PG-SGA) as a nutrition assessment tool in patients with cancer. European journal of clinical nutrition 2002;56:779-85.
106. Detsky AS, McLaughlin JR, Baker JP, et al. What is subjective global assessment of nutritional status? JPEN Journal of parenteral and enteral nutrition 1987;11:8-13.
112. Mitchell AJ, Coyne JC. Do ultra-short screening instruments accurately detect depression in primary care?: A pooled analysis and meta-analysis of 22 studies. The British Journal of General Practice 2007;57:144-51.
113. Arroll B, Khin N, Kerse N. Screening for depression in primary care with two verbally asked questions: cross sectional study. BMJ (Clinical research ed) 2003;327:1144-6.
114. Gilbody S, House AO, Sheldon TA. Screening and case finding instruments for depression. The Cochrane database of systematic reviews 2005:Cd002792.
115. Whooley MA, Avins AL, Miranda J, Browner WS. Case-finding instruments for depression. Two questions are as good as many. J Gen Intern Med 1997;12:439-45.
116. Janda M, DiSipio T, Hurst C, Cella D, Newman B. The Queensland Cancer Risk Study: general population norms for the Functional Assessment of Cancer Therapy-General (FACT-G). Psychooncology 2009;18:606-14.
117. Pickard AS, De Leon MC, Kohlmann T, Cella D, Rosenbloom S. Psychometric comparison of the standard EQ-5D to a 5 level version in cancer patients. Med Care 2007;45:259-63.
118. Herdman M, Gudex C, Lloyd A, et al. Development and preliminary testing of the new five-level version of EQ-5D (EQ-5D-5L). Quality of life research : an international journal of quality of life aspects of treatment, care and rehabilitation 2011;20:1727-36.
119. Sorror ML, Maris MB, Storb R, et al. Hematopoietic cell transplantation (HCT)-specific comorbidity index: a new tool for risk assessment before allogeneic HCT. Blood 2005;106:2912-9.
120. Sorror ML, Giralt S, Sandmaier BM, et al. Hematopoietic cell transplantation-specific comorbidity index as an outcome predictor for patients with acute myeloid leukemia in first remission: combined FHCRC and MDACC experiences. Blood 2007;110:4606-13.
121. Maruyama D, Fukuda T, Kato R, et al. Comparable antileukemia/lymphoma effects in nonremission patients undergoing allogeneic hematopoietic cell transplantation with a conventional cytoreductive or reduced-intensity regimen. Biology of blood and marrow transplantation : journal of the American Society for Blood and Marrow Transplantation 2007;13:932-41.

122. Farina L, Bruno B, Patriarca F, et al. The hematopoietic cell transplantation comorbidity index (HCT-CI) predicts clinical outcomes in lymphoma and myeloma patients after reduced-intensity or non-myeloablative allogeneic stem cell transplantation. Leukemia : official journal of the Leukemia Society of America, Leukemia Research Fund, UK 2009;23:1131-8.
123. Sorror ML, Sandmaier BM, Storer BE, et al. Comorbidity and disease status-based risk stratification of outcomes among patients with acute myeloid leukemia or myelodysplasia receiving allogeneic hematopoietic cell transplantation. Journal of Clinical Oncology 2007;25:4246-54.
124. Sorror ML, Storer BE, Maloney DG, Sandmaier BM, Martin PJ, Storb R. Outcomes after allogenic hematopoietic cell transplantation with nonmyeloablative or myeloablative conditioning regimens for treatment of lymphoma and chronic lymphocytic leukemia. Blood 2008;111:446-52.
125. Sorror ML, Sandmaier BM, Storer BE, et al. Long-term Outcomes Among Older Patients Following Nonmyeloablative Conditioning and Allogeneic Hematopoietic Cell Transplantation for Advanced Hematologic Malignancies. Jama-J Am Med Assoc 2011;306:1874-83.
126. Sorror ML, Storb RF, Sandmaier BM, et al. Comorbidity-Age Index: A Clinical Measure of Biologic Age Before Allogeneic Hematopoietic Cell Transplantation. Journal of Clinical Oncology 2014;32:3249-56.
127. Distress management. Clinical practice guidelines. Journal of the National Comprehensive Cancer Network : JNCCN 2003;1:344-74.
128. O'Donnell E. The distress thermometer: a rapid and effective tool for the oncology social worker. International journal of health care quality assurance 2013;26:353-9.
129. NCCN Distress Thermometer and Problem List. 2017. (Accessed October 31, 2017, at https://www.nccn.org/about/permissions/thermometer.aspx.)
130. Bateman SL, Seed PC. Epigenetic regulation of the nitrosative stress response and intracellular macrophage survival by extraintestinal pathogenic Escherichia coli. Molecular microbiology 2012;83:908-25.
131. Ubeda C, Taur Y, Jenq RR, et al. Vancomycin-resistant Enterococcus domination of intestinal microbiota is enabled by antibiotic treatment in mice and precedes bloodstream invasion in humans. The Journal of clinical investigation 2010;120:4332-41.
132. Caporaso JG, Kuczynski J, Stombaugh J, et al. QIIME allows analysis of high-throughput community sequencing data. Nature methods 2010;7:335-6.
133. Johnston-Wilson NL, Sims CD, Hofmann JP, et al. Disease-specific alterations in frontal cortex brain proteins in schizophrenia, bipolar disorder, and major depressive disorder. The Stanley Neuropathology Consortium. Molecular psychiatry 2000;5:142-9.
134. Lamers KJB, Vos P, Verbeek MM, Rosmalen F, van Geel WJA, van Engelen BGM. Protein S-100B, neuron-specific enolase (NSE), myelin basic protein (MBP) and glial fibrillary acidic protein (GFAP) in cerebrospinal fluid (CSF) and blood of neurological patients. Brain Research Bulletin 2003;61:261-4.
135. Common Terminology Criteria for Adverse Events (CTCAE) Version 4.03. In: U.S. Department of Health and Human Services NIoH, National Cancer Institute, ed.2010.
136. Gold MR. Cost-effectiveness in health and medicine. New York: Oxford University Press; 1996.
137. Reed SD, Friedman JY, Gnanasakthy A, Schulman KA. Comparison of hospital costing methods in an economic evaluation of a multinational clinical trial. International journal of technology assessment in health care 2003;19:396-406.
138. A comprehensive guidebook to the DRG classification system. 29 ed: Ingenix; 2013.
139. PFS National Payment Amount file. Centers for Medicare & Medicaid Services. (Accessed September 23, 2013, at http://www.cms.gov/PhysicianFeeSched/01_overview.asp.)
140. Pharmacy Benefits Management Services. United States Department of Veterans Affairs. (Accessed September 23, 2013, at http://www.pbm.va.gov/drugpharmaceuticalprices.asp.)
141. Blazar BR, Murphy WJ, Abedi M. Advances in graft-versus-host disease biology and therapy. Nature reviews Immunology 2012;12:443-58.

142. Reed SD, Li Y, Kamble S, et al. Introduction of the Tools for Economic Analysis of Patient Management Interventions in Heart Failure Costing Tool: a user-friendly spreadsheet program to estimate costs of providing patient-centered interventions. Circulation Cardiovascular quality and outcomes 2012;5:113-9.
143. Robinson J. Hospitals respond to Medicare payment shortfalls by both shifting costs and cutting them, based on market concentration. Health affairs 2011;30:1265-71.
144. Reinhardt UE. Spending more through 'cost control:' our obsessive quest to gut the hospital. Health affairs 1996;15:145-54.
145. White JR, Nagarajan N, Pop M. Statistical methods for detecting differentially abundant features in clinical metagenomic samples. PLoS computational biology 2009;5:e1000352.
146. Oksanen J G-BF, Kindt R, Legendre P, Minchin PR, O'Hara RB, Simpson GL, Solymos P, Henry M, Stevens H, Wagner H. vegan: Community Ecology Package. R package version 2.0-9. 2013.
147. McMurdie PJ, Holmes S. phyloseq: an R package for reproducible interactive analysis and graphics of microbiome census data. PloS one 2013;8:e61217.
148. Paradis E, Claude J, Strimmer K. APE: Analyses of Phylogenetics and Evolution in R language. Bioinformatics 2004;20:289-90.
149. Liaw A WM. Classification and Regression by randomForest. R News 2002;2:18-22.
150. Hahsler M GB, Hornik K (2005) arules -- A Computational Environment for Mining Association Rules and Frequent Item Sets. Journal of Statistical Software 2005;14.
151. Sim J, Lewis M. The size of a pilot study for a clinical trial should be calculated in relation to considerations of precision and efficiency. J Clin Epidemiol 2012;65:301-8.

12.0 **Appendices**

12.1 <u>APPENDIX A</u>: PATIENT REPORTED OUTCOME INSTRUMENTS

1) **Physical Activity and Function**
    a. PROMIS Physical Function—see attached
2) **Cognitive Function**
    a. PROMIS Cognitive—see attached
    b. RBANS—see attached
    c. Trail Making Test--see attached
    d. Brief Cope—see attached
    e. Montreal Cognitive Assessment
3) **Mental Health**
    a. National Comprehensive Cancer Network Distress Thermometer
    b. PHQ2/PHQ9
    c. PCL-5
    d. PROMIS Depression—see attached
    e. PROMIS Anxiety—see attached
4) **Nutrition and Diet**
    a. PG-SGA—see attached
5) **Social Support**
    a. PROMIS Emotional Support—see attached
    b. PROMIS Social Isolation—see attached
    c. Ego Resiliency—see attached
    d. Two Factor Consideration of Future Consequences Scale (CFC-14)—see attached
    e. Brief Resilience Scale—see attached
6) **Global Assessments**
    a. FACT-BMT
    b. EQ-5D-5L
    c. OARSf IADL
    d. TRU-BMT/SplendoFit
7) **Caregiver Assessments**
    a. Caregiver Strain—see attached
    b. Preparedness for Caregiving—see attached
    c. FACT-GP – see attached
    d. PHQ2/ PHQ9 –see attached
    e. PROMIS Depression—as above
    f. PROMIS Anxiety – as above
    g. PROMIS Emotional Support – as above
    h. PROMIS Social Isolation—as above
8) **Patient Reported BMT Questionnaire**
    a. See Appendix E

12.2 APPENDIX B: Hand Grip Testing

**I. Background and Purpose**	Grip strength, as measured by a handheld dynamometer, has been reported to be a good predictor of future functional limitations and disabilities. Patients in the lower percentiles of grip strength (along with Karnofsky Performance Status) generally had more limitations as they aged (1). Moreover, handgrip strength was a powerful predictor of morality related to all-cause mortality and cardiovascular disease for both men and women after accounting for several factors (2,3).<br><br>Protocols for using hand-held dynamometers to measure grip strength have been the subject of much research due to the potential variability of the data. The positioning of the patient (e.g. supine vs. standing or arm straight out vs. elbow bent), the variability in the administration of the test (e.g. one long test vs. multiple subsequent tests or long vs. short rests), and the analysis of the results (avg. of several tests vs. maximum output) have affected results.<br><br>The present protocol will standardize the way in which grip strength is measured and analyzed within DCCRP. The American Society of Hand Therapists recommends recording the average of three measurements, whereas the American Society for Surgery of the Hand (ASSH) recommends using the maximum output of three measurements (4,5). Haider et al (2004) examined these protocols and found that both produce similar and consistent results with a 95% confidence interval, and found that 25% of maximum outputs were recorded on the second and third try. Haider recommended the use of the ASSH protocol (5) because of the practical benefit of not using additional math in the clinical setting (6). Hence, DCCRP will follow the ASSH protocol.<br><br>Additionally, research has shown that waiting at least one minute between grip strength measurements improves the consistency of the output (7). Ideally, grip strength would be measured with the patient sitting and resting elbows at 90° on a table (7, 8); however, because not all clinics have available tables, DCCRP will use other methods, such as using a chair with arms, when necessary.
**II. Scope**	This procedure applies to all studies using grip strength measurement as a variable over time.
**III. Definitions**	<u>*Grip Strength*</u>—A measure of how hard someone can squeeze that is a good indicator of upper body strength (8).<br><br><u>*Dynamometer*</u>—An instrument used to measure grip strength.<br><br><u>*Maximum Output*</u> –The greatest measure (kg) obtained from multiple trials of a grip strength test.
**IV. Responsibilities**	Clinical Research Coordinators and Clinical Research Nurses will need to use this

	protocol in relevant DCCRP studies.
**V. Procedure**	**Equipment**<br>**a.** Jamar Handheld Dynamometer (Promedics Ltd., Blackburn, Lancashire, UK)<br>**b.** Table and straight back chair (if available)<br>**c.** Data collection form<br>**d.** Watch with a second hand or digital second counter<br><br>**Nurses or Clinical Research Coordinators (CRNs or CRCs)**<br><br>**a.** Sitting across the table from the participant, demonstrate how to hold the dynamometer. Then explain to them the process that will take place.<br>**b.** Tell the patient the following:<br><br>***"I am going to demonstrate for you how this test is going to be done. First, I will place this instrument in your right hand while your arm is resting on the table. Please be careful when I place it in your hand as it may be heavier than you are expecting."***<br>***"I will then ask you to start to squeeze the instrument while I say:***<br><br>***"Squeeze as hard as you can…harder!...harder!...relax."***<br><br>**Demonstrate the process for the participant with script.**<br><br>***"I am going to repeat this exact process 3 times for each hand. You will be given the opportunity to rest for 1 full minute between each attempt. Do you understand?"***<br>**c.** If the participant understands and agrees to participate, begin the procedure. If he or she does not understand, repeat the instructions detailed in (**b**.). **DO NOT ALLOW THE PARTICIPANT TO PRACTICE!**<br>**d.** Set-up:<br>**i.** This test should be done with the participant in a seated position with feet flat on the floor.<br>**ii.** Record date and time of the administration of the test on the data collection form.<br>**iii.** Determine whether the participant is right- or left- handed and record hand dominance on data collection form.<br>**e.** Positioning the patient:<br>**i.** It is ideal to place the participant's right arm on a table.<br>**1.** If a table is not available, a chair with arms is the next best option (available in the waiting room of most clinics)<br>**2.** If a chair with arms is not available, kneel down and support the patient's wrist with your hand<br>**ii.** The patient's shoulder should be adducted and neutrally rotated<br>**iii.** Elbow should be flexed at 90 degrees.<br>**iv.** Forearm should be in a neutral position

	**v.** Wrist should be between 0 and 30 degrees extended<br>**f.** Ensure that the arrow within the gauge is set to 0.<br>**g.** Verify the participant is in the correct position<br>**h.** Place the dynamometer in the RIGHT hand of the participant.<br>**i.** Recite the script as below:<br><br>***"Squeeze as hard as you can...harder!...harder!...relax."***<br><br>**j.** Remove the dynamometer from the participant's hand.<br>**i.** Record if the participant was able to complete the test. Please record the reason in the section marked *additional notes* if unable to complete.<br>**ii.** Record the value (to the nearest 2 kilograms) on the data collection form.<br>**k.** Wait for one full minute (60 seconds) from the time you removed the instrument from the participant's hand.<br>**l.** Repeat (**f**.) through (**k**.) for two additional times with the participant's right hand.<br>**m.** After completion of the third test in the participant's right hand, repeat (**f**.) through (**k**.) with the participant's his/her *LEFT* hand.<br>**n.** Maintenance and Calibration<br>**i.** The calibration and documentation of the Jamar Dynamomenter is the responsibility of the Clinical Research Coordinator within the Duke Cancer Care Research Program.<br>**ii.** The dynamometer should be re-calibrated yearly. The CRC/CRN must mail-in the dynamometer to its respective company for re-calibration. The day, month, and year in which the instrument was sent for re-calibration should be recorded. In the event that the instrument was dropped or mishandled, it should be immediately mailed to the company for repairs. Record the date.
**VI. References**	1. Rantanen T, Guralnik JM, Foley D, et al. **Midlife hand grip strength as a predictor of old age disability.** *JAMA*. 1999;281;558-560.<br><br>2. Rantanen T, Volpato S, Ferrucci L, Heikkinen, Fried LP, Guralnik JM. **Handgrip Strength and Cause-Specific and Total Mortality in Older Disables Women: Exploring the Mechanism.** *JAGS,* 2003 (51): 636-641.<br><br>3. Gale CR, Martyn CN, Cooper C, Sayer AA. **Grip Strength, body composition, and mortality**. *International Journal of Epidemiology, 2007 (36): 228-235.*<br><br>4. Bohannon RW. **Hand grip dynamometers: issues relevant to application.** *Journal of Human Muscle Performance*, 1991; 1: 16–36.<br><br>5. Smith RO, Benge MW. **Pinch and grasp strength: standardization of**

	**terminology and protocol.** *American Journal of Occupational Therapy*, 1985, 19: 531–535.<br><br>6. Haider SG, Kumar D, Bassi S, Deshmukh SC. **Average versus maximum grip strength: which is more consistent?** *J Hand Surg: J Brit Soc Surg Hand*, Feb 2004; **29**:1; 82-84<br><br>7. Watanabe T, Owashi K, Kanauchi Y, Mura N, Takahara M, Ogino T. **The Short-Term Reliability of Grip Strength Measurement and the Effects of Posture and Grip Span.** *J Hand Surg*, 2005, **30**: 3; 603-609.<br><br>8. Innes E. **Handgrip strength testing: A review of the literature**. *Australian Occupational Therapy Journal.* 1999. 46. 120-140.<br><br>9. Hillman T.E., Nunes Q.M., Hornby S.T., Stanga Z., Neal K.R., Rowlands B.J., Allison S.P., Lobo D.N. **A practical posture for hand grip dynamometry in clinical setting.** *Clinical Nutrition.* 2005. 24, 224-228.

12.3 APPENDIX C: NON-ENROLLMENT SURVEY (Decline Survey)

I am generally uneasy with the research process.	Agree	Disagree
The clinical study is too complex.	Agree	Disagree
The clinical study is inconvenient to everyday life.	Agree	Disagree
I am worried about side effects of exercise.	Agree	Disagree
Exercise is not appropriate for my disease.	Agree	Disagree
Exercise is not the best option for my disease.	Agree	Disagree
Exercise has no benefits for me.	Agree	Disagree
I prefer alternatives to exercise, to maintain my health.	Agree	Disagree
I was not provided with enough information about this clinical study.	Agree	Disagree
I am worried about the cost to my health insurance.	Agree	Disagree
I am worried about transportation or travel distance.	Agree	Disagree
I do not have the family support to participate in this study.	Agree	Disagree
I am uncomfortable with experimentation.	Agree	Disagree
I feel anxious participating in this study.	Agree	Disagree
I feel uncertain about participating in this study.	Agree	Disagree
I feel my quality of life might be reduced.	Agree	Disagree
I do not trust the research process or researchers involved.	Agree	Disagree
I am concerned about the effect of the doctor-patient relationship with this study.	Agree	Disagree
I believe the doctor should make the decision about participation.	Agree	Disagree
My doctor did not talk to me about this study.	Agree	Disagree

12.4 APPENDIX D: Patient Reported BMT Questionnaire

1. NPE: Have you identified one consistent caregiver for the duration of transplant? (yes/no)
   a. If no: Are you looking for one primary caregiver or are you thinking of a team of caregivers or unsure? (one, team, unsure)
